# Differential cognitive and behavioral development from 6 to 24 months in autism and fragile X syndrome

**DOI:** 10.1186/s11689-024-09519-y

**Published:** 2024-03-20

**Authors:** Lindsay J. Mullin, Joshua Rutsohn, Julia L. Gross, Kelly E. Caravella, Rebecca L. Grzadzinski, Leigh Anne Weisenfeld, Lisa Flake, Kelly N. Botteron, Stephen R. Dager, Annette M. Estes, Juhi Pandey, Robert T. Schultz, Tanya St. John, Jason J. Wolff, Mark D. Shen, Joseph Piven, Heather C. Hazlett, Jessica B. Girault

**Affiliations:** 1 Carolina Institute for Developmental Disabilities, the University of North Carolina at Chapel Hill, Chapel Hill, USA; 2 Department of Biostatistics, the University of North Carolina at Chapel Hill, Chapel Hill, USA; 3 Department of Psychiatry, University of North Carolina at Chapel Hill, Chapel Hill, USA; 4 Department of Psychiatry, Washington University School of Medicine, St. Louis, USA; 5 Department of Radiology, University of Washington, Seattle, USA; 6 Center On Human Development and Disability, University of Washington, Seattle, USA; 7 Department of Speech and Hearing Sciences, University of Washington, Seattle, USA; 8 The Children’s Hospital of Philadelphia and University of Pennsylvania, Center for Autism Research, Philadelphia, USA; 9 Department of Educational Psychology, University of Minnesota, Minneapolis, USA; 10 Neuroscience Center, University of North Carolina at Chapel Hill, Chapel Hill, USA

**Keywords:** Autism, Fragile X syndrome, Infancy, Development, Behavioral, Cognitive

## Abstract

**Background:**

Specifying early developmental differences among neurodevelopmental disorders with distinct etiologies is critical to improving early identification and tailored intervention during the first years of life. Recent studies have uncovered important differences between infants with fragile X syndrome (FXS) and infants with familial history of autism spectrum disorder who go on to develop autism themselves (FH-ASD), including differences in brain development and behavior. Thus far, there have been no studies longitudinally investigating differential developmental skill profiles in FXS and FH-ASD infants.

**Methods:**

The current study contrasted longitudinal trajectories of verbal (expressive and receptive language) and nonverbal (gross and fine motor, visual reception) skills in FXS and FH-ASD infants, compared to FH infants who did not develop ASD (FH-nonASD) and typically developing controls.

**Results:**

Infants with FXS showed delays on a nonverbal composite compared to FH-ASD (as well as FH-nonASD and control) infants as early as 6 months of age. By 12 months an ordinal pattern of scores was established between groups on all domains tested, such that controls > FH-nonASD > FH-ASD > FXS. This pattern persisted through 24 months. Cognitive level differentially influenced developmental trajectories for FXS and FH-ASD.

**Conclusions:**

Our results demonstrate detectable group differences by 6 months between FXS and FH-ASD as well as differential trajectories on each domain throughout infancy. This work further highlights an earlier onset of global cognitive delays in FXS and, conversely, a protracted period of more slowly emerging delays in FH-ASD. Divergent neural and cognitive development in infancy between FXS and FH-ASD contributes to our understanding of important distinctions in the development and behavioral phenotype of these two groups.

**Supplementary Information:**

The online version contains supplementary material available at 10.1186/s11689-024-09519-y.

## Background

Younger biological siblings of children with autism spectrum disorder (ASD) have higher-than-typical likelihood of receiving an ASD diagnosis, with approximately 20% of younger siblings meeting diagnostic criteria in toddlerhood [1], compared to the population prevalence of 2.8% [2]. The increased likelihood for ASD in infants with a family history (FH) reflects the high heritability of ASD in families, which arises primarily from common polygenic variation [3, 4]. Longitudinal studies of FH infants create an opportunity to prospectively study cohorts of children who will later go on to develop ASD (FH-ASD). This paradigm allows researchers to observe emerging differences in behavior and neurobiology that precede diagnostic indicators, identifying the earliest predictors of ASD development and potential targets for intervention [5]. To date, infant sibling studies have reported specific brain differences from typical development in FH-ASD infants as early as 6 months of age [5–7] and diagnosable traits of ASD such as language and social deficits and stereotyped and repetitive behaviors starting in the second year of life [6]. FH-ASD infants also display lower cognitive and receptive language scores at 12 months of age when compared to FH infants who do not develop ASD (FH-nonASD) and control infants [8, 9]. Cognitive trajectories have also been shown to vary among FH-ASD infants; approximately one-third exhibit declines across the first two years of life [10] with implications for adaptive behavior in toddlerhood [11].

Fragile X syndrome (FXS) is a neurodevelopmental condition occurring in 1 out of 5,000–10,000 individuals [12, 13], resulting from a hereditary mutation on the X chromosome which reduces production of fragile X messenger ribonucleoprotein (FMRP) [14]. The reduction of FMRP in FXS leads to a set of brain [15–19] and behavioral [20–28] differences from typical development, some of which are detectable as early as 6 months of age. FXS can present differently in males and females, due to the second, typically unaffected, X chromosome in females with FXS, resulting in preservation of some FMRP. This sex-based effect leads to a more homogenous phenotype in males (i.e., 96% with intellectual disability [29], greater autistic features) compared to greater heterogeneity in females (i.e., a full range of cognitive abilities [30]). Early developmental delays in infants with FXS reflect the global developmental delay that is characteristic of this population [26] and spans motor skills [20, 21, 25, 26], visual reception [20, 25, 26], language development [20, 22, 25–28], social communication [24], and adaptive behaviors [23]. FXS also shares behavioral features with ASD [31], with 20% to 75% of individuals with FXS meeting diagnostic criteria for ASD [23, 32, 33].

Prospective studies comparing FH infants and FXS infants provide a unique opportunity to examine the etiologically unique developmental trajectories of two groups with overlapping behavioral phenotypes. Previous studies comparing FXS and FH infants (pooling FH-ASD and FH-nonASD infants into a single group) have reported that FXS infants have lower skill levels compared to combined FH samples in the domains of adaptive behaviors by 9 months of age [23], social communication in infants ages 7.5 to 14.5 months [24], and developmental composite, language, and motor abilities by 6 months of age [25]. The pooling of FH infants with and without an ASD diagnosis may inflate these group differences with FXS, as a large portion of FH infants will develop typically [8, 34]. Comparing FH-ASD (as a separate group from FH-nonASD) and FXS during infancy provides a novel contrast which may have important implications for pharmacological [35] and/or behavioral interventions that focus on improving autistic and cognitive symptoms in these groups.

Our group recently compared brain and behavioral development in FXS and FH-ASD infants from 6 to 24 months, reporting lower overall cognitive ability in FXS infants compared to FH-ASD infants starting at 6 months of age and continuing through 24 months of age [19]. Enlarged caudate volume distinguished infants with FXS from FH-ASD infants and was associated with greater repetitive behaviors in FXS infants but not in FH-ASD infants. The differential neurobiology may underly the observed differences in cognitive and ASD-related traits between FXS and FH-ASD infants. These findings further highlight the presence of a presymptomatic period in FH-ASD [36], whereby differences in neurodevelopment emerge slowly over the course of the first years of life. This suggests an age- and disorder-specific pattern of cascading brain changes leading to ASD that is distinct from that observed in FXS [19]. While this report demonstrates global developmental differences by 6 months of age between FXS and FH-ASD infants, it remains unknown whether specific domains of early skills (e.g., expressive language, fine motor) show detectable group differences in the first two years of life.

Given the gaps in knowledge about specific, early, and longitudinal developmental differences between infants with FXS and FH-ASD infants, we compared neurocognitive trajectories from 6 to 24 months of age on several skill domains including gross and fine motor, expressive and receptive language, and visual reception, as well as verbal and nonverbal cognitive composite scores. This study is the first of its kind to examine similarities and differences in developmental trajectories between FH-ASD and FXS groups during infancy. This work was guided by the following research goals and hypotheses:

1. Compare developmental trajectories throughout infancy between FH-ASD and FXS infants, examining the timing and extent of these groups’ divergence from typical development as well as their divergence from each other, on specific skill domains and verbal and nonverbal composites. We hypothesize that characteristic patterns of cognitive abilities in FH-ASD (i.e., milder delays) and FXS (i.e., pronounced global delays) observed later in development will emerge across infancy. We expect that FXS infants will diverge from control infants earlier and to a greater extent than FH-ASD, demonstrative of a presymptomatic period in FH-ASD that is not present in FXS.
2. Further specify FH-ASD and FXS group differences by delineating the influence of cognitive level on skill trajectories for FH-ASD, given the wide range of cognitive ability in this group. We hypothesize that FH-ASD infants with lower cognitive levels will score lower on MSEL domains than the FH-ASD group with higher cognitive level, following trajectories more similar to the FXS group throughout infancy.

## Method

### Participants

Infants included in the current investigation participated in two multisite longitudinal studies of brain and behavioral development encompassing three groups: 1) infants with FXS, 2) infants with at least one older full-biological sibling with autism (family history; FH), and 3) infants with a typically developing older sibling and no siblings diagnosed with autism or FXS (control). Infants with FXS were recruited via postings in list serves, family advocacy conferences, research registries, and through a FXS specialty clinic. By design, the intended enrollment age was 6 months, though FXS and FH infants were allowed to enroll at 12 months, and in rare cases 24 months, to increase recruitment in these groups. FXS diagnosis was confirmed by medical records or genetic testing, and full-mutation FXS was confirmed via genetic testing record for all but three participants, two of whom we could not obtain records for, and one participant with mosaicism. We re-ran all analyses excluding the participant with confirmed mosaicism, and effects were nearly identical, so we maintained inclusion of this infant in analyses. Diagnosis of ASD in the older sibling of the infants in the FH group was confirmed through records from a clinical provider and supported by administration of the Autism Diagnostic Interview-Revised (ADI-R) [37] by research staff. Exclusionary criteria included genetic, medical, neurological, and sensory conditions known to affect development in the infant (other than FXS in the FXS group), premature birth or low birth weight (< 2,000 grams), in utero exposure to exogenous compounds likely to adversely affect brain development, contraindication for magnetic resonance imaging (MRI), infant adoption, non-English-speaking family, and any history of intellectual disability, psychosis, schizophrenia, or bipolar disorder in a first degree relative[18, 38]. Research protocols were approved by the Institutional Review Boards at the data collection sites: the University of North Carolina at Chapel Hill, the University of Washington, Children’s Hospital of Philadelphia, and Washington University in St. Louis. Parents provided informed consent for their infants to participate.

### Clinical evaluation

Participants in the FH group were given a clinical best estimate judgment for autistic disorder or pervasive developmental disorder not otherwise specified at the 24-month visit using DSM-IV-TR [39] criteria, the Autism Diagnostic Observation Schedule–Generic (ADOS) [40], and the ADI-R, by the clinician who conducted the behavioral assessments and confirmed via video review by a senior psychologist or psychiatrist who was blind to FH status. Participants meeting criteria for either disorder were classified as having an ASD outcome in the study, consistent with DSM-5 [41] criteria which became available after the original study concluded.

ASD classification based on either the DSM, ADOS, or both, at 24 months was only available for 52% of participants in the FXS cohort (*n* = 16). Missing or unreliable data on the remaining FXS infants (*n* = 15) was due to a range of factors (e.g., fatigue/fussiness, low mental age, and inconsistent ability to capture reliable information from the prompts in the assessments) or loss to follow up at the 24-month timepoint. Thus, ASD outcome within the group of FXS infants was not analyzed in this study. This is consistent with prior work from our team involving these infant groups [19]. See Table S12 in the Supplement for 24-month ASD data availability for each FXS participant. The final sample included: 77 FH-ASD infants (63 male, 14 female); 280 infants with family history who were not diagnosed with autism (FH-nonASD; 156 male, 124 female); 31 infants with FXS (25 male, 6 female); and 154 control infants (91 male, 63 female).

### Behavioral assessments

Participants were administered a comprehensive battery of early developmental behavioral assessments at 6, 12, and 24 months of age, including parent-report and direct assessment measures of adaptive behavior, repetitive behaviors, social communication, and cognitive development. The primary measure of interest in this investigation is the Mullen Scales of Early Learning (MSEL) [42]. The MSEL is a comprehensive developmental assessment that covers the domains of language, motor, perceptual abilities, and cognition in infants and young children. Subtests of the MSEL include receptive language (RL), expressive language (EL), fine motor (FM), gross motor (GM), and visual reception (VR). One control infant displayed floor effects (e.g., T-scores of 20) at one or more timepoints on each domain of the MSEL, and was therefore excluded from the current investigation for suspicion of global developmental delay, not representing typical development.

### MSEL measures assessed

Developmental trajectories for FXS, FH-ASD, FH-nonASD, and control infants were examined for each domain of the MSEL (RL, EL, FM, GM, and VR) to identify whether differential skill development in specific behavioral domains at specific ages emerged among FXS and FH-ASD groups in infancy.

The primary analyses utilized raw scores from the MSEL. Raw scores reflect the number of items successfully completed on each subscale, with higher scores indicating more advanced skill development. Raw scores represent a constant rate of development within and across subscales which allow for best group comparisons. Raw scores are better suited for studying the group of FXS infants in the current study than standard scores given their greater range and ability to overcome floor effects often observed using standard scores (e.g., T-scores of 20) on the MSEL [20]. Raw scores additionally allow for interpretation of developmental trajectories in the context of growth, where most infants’ scores will improve over time as their skills develop, in contrast to standard scores which must be interpreted in the context of typical development represented by a mostly flat profile over time (i.e., an average person having an average standard score throughout development). Though age equivalent scores have psychometric limitations [43], supplemental analyses utilizing age equivalent scores from the MSEL are included as part of the current study, to provide a direct comparison to other published reports of infants with FXS that utilized age equivalent scores (e.g., Wheeler et al., 2021 [20]).

To expand upon our previous report of group differences on the MSEL Early Learning Composite (ELC) measure [19], and for comparison to other work [20], verbal developmental quotient (VDQ) and nonverbal developmental quotient (NVDQ) scores were generated and utilized in a subset of analyses focused on overall cognitive domains. The VDQ is a language-based composite measure, calculated by averaging the age equivalent scores of RL and EL domains, dividing by chronological age, and multiplying by 100. The NVDQ is calculated by averaging the FM and VR age equivalent scores, dividing by chronological age, and multiplying by 100. The NVDQ has been found to be more predictive of school-aged executive functioning than language-reliant developmental quotients [44] and has been found to be associated with specific deficits in white matter development (i.e., lower fractional anisotropy in the left and right uncinate fasciculus), characteristic of FXS at 12 months of age [18].

The analysis of longitudinal group differences using the VDQ and NVDQ allows for a parsing of previous findings of FXS and FH-ASD divergence on the ELC [19], which comprises visual reception, fine motor, and both language scales, into more specific skill composites where verbal and nonverbal abilities are analyzed separately while maintaining the use of composites which include information from multiple domains. Additionally, longitudinal analysis of NVDQ throughout infancy between the groups included in the current study may eliciduate group differences very early in development on this measure of cognitive ability which has been previously shown to be related to early brain differences between FXS and control infants [18].

### Statistical analysis

Separate mixed-effects models estimated the changes in MSEL RL, EL, FM, GM, VR, VDQ, and NVDQ scores from 6 to 24 months of age. These models included random intercepts for each participant. Independent variables included group membership, timepoint, sex, and study site. These latter two variables were included to control for effects related to differences in sex ratio among groups and any potential unaccounted variance related to assessment site. Models also included the interaction of group by age. Trajectories of development for FXS and FH groups were examined in reference to the control group, to understand differences between FXS and FH infants in comparison to typical development. Missing data were assumed missing at random with patterns of missingness not varying by group or other covariates. Approximately 72% of the participants had no missing MSEL data at any of the visits, and 13% of the sample had missing data at the 6-month visit. The rest of the sample had some missingness at either the 12-month visit, 24-month visit, or a combination of two visits among the 6-, 12-, and 24-month visits. Missingness was handled via multiple imputations (*m* = 5) obtained through multivariate imputation by chained equations. This method fits a sequence of regression models for the missing value and imputes the missing data from its predictive distribution [45]. These chained equations incorporated every covariate in the mixed-effects model to predict the missing value. Predicted values were produced using predictive mean matching with fully conditional specification [46]. Fixed-effects estimates were pooled according to Rubin’s rules [47]. Imputation of missing scores provided more power and mitigated the possibility of bias introduced from listwise deletion of data [48–51] (see Table 1 for MSEL availability by group and timepoint before imputation). Sensitivity analyses were performed to determine comparability of imputed versus unimputed analyses. The two sets of estimates were deemed comparable (see Table S2 in the Supplement for unimputed results). Least-squares means were estimated to compare groups at different visits with Cohen’s *d* effect sizes reported. Cohen’s *d* was calculated using the lme.dscore() function from the EMAtools package [52]. Multiple imputations were estimated using the mice package in R [53], mixed-effects models were estimated using the nlme package [54], and least-squares means were estimated using the emmeans package [55].

**Table 1:** Group demographics and data availability before imputation

	**Group**			
	FXS	FH-ASD	FH-nonASD	Control
**Age, Mean (SD)**				
**6-month timepoint**	6.89 (1.02)	6.53 (0.65)	6.60 (0.68)	6.70 (0.73)
**12-month timepoint**	12.5 (0.75)	12.7 (0.73)	12.6 (0.58)	12.7 (0.73)
**24-month timepoint**	24.4 (0.82)	24.9 (1.47)	24.8 (1.03)	24.7 (1.06)
**Sex, n (%)**				
**Female**	6 (19%)	14 (18%)	124 (44%)	63 (41%)
**Male**	25 (81%)	63 (82%)	156 (56%)	91 (59%)
**Race, n (%)**				
**White**	24 (77%)	65 (84%)	241 (86%)	126 (82%)
**African American, Black**	0 (0%)	2 (3%)	5 (2%)	9 (6%)
**Asian**	0 (0%)	0 (0%)	4 (1%)	2 (1%)
**American Indian, Alaskan Native**	0 (0%)	0 (0%)	0 (0%)	0 (0%)
**More than one race**	4 (13%)	9 (12%)	27 (10%)	15 (10%)
**Not available**	3 (10%)	1 (1%)	3 (1%)	2 (1%)
**Ethnicity, n (%)**				
**Hispanic**	1 (3%)	4 (5%)	21 (8%)	9 (6%)
**Non-Hispanic**	26 (84%)	71 (92%)	255 (91%)	143 (93%)
**Not available**	4 (13%)	2 (3%)	4 (1%)	2 (1%)
**MSEL Availability, n (%)**				
**6-month timepoint**	20 (65%)	61 (79%)	230 (82%)	142 (92%)
**12-month timepoint**	24 (77%)	68 (88%)	263 (94%)	139 (90%)
**24-month timepoint**	22 (71%)	75 (97%)	278 (99%)	153 (99%)

### Supplemental analyses

Because cognitive level varies greatly within FH-ASD infants and is related to ASD severity [8] and behavioral trajectories [10], we further divided the FH-ASD group to assess the effect of low cognitive level within FH-ASD on MSEL developmental trajectories in comparison to the FXS group, which is characterized by low cognitive level. We parsed FH-ASD infants by whether they had a MSEL ELC score of 70 or lower, which equates to 2 standard deviations below the mean (FH-ASD-Low) at the latest available timepoint in infancy. Group trajectories between FH-ASD-Low (*n* = 24), FH-ASD-Avg/High (*n* = 58) and FXS were compared (most of the FXS infants met criteria for low cognitive level, characteristic of this group, see Tables S8, S9, S10 and S11 in the Supplement).

As described above, to provide alternative interpretations to our group comparisons and to investigate whether group trajectories differed dependent on score type used, all analyses were run with age equivalent scores and are presented in Tables S3 and S4 and Figs. S1, S2, S3, S4 and S5 of the Supplement.

Due to the inherent variability in behavioral phenotypes between males and females with FXS, supplementary analyses were conducted to investigate whether patterns of group differences (FXS vs. FH-ASD) in male infants were similar in nature to the combined sample of males and females utilizing both raw and age equivalent scores. Females were not investigated separately due to low sample size for FXS (*n* = 6); this approach is consistent with other studies of infants with FXS [24].

## Results

### Differential development throughout infancy for FXS and FH-ASD

Participant characteristics by group before imputation are summarized in Table 1. Given the potential role of socioeconomic status on child cognitive development, we tested for group differences in maternal education levels in the FXS and FH-ASD groups (the primary contrast of interest) and found no differences (see Table S7 in the Supplement). Table 2 provides the results of the regression with Cohen’s *d* values, and Table 3 provides the least-squares means contrasts of VDQ, NVDQ and each MSEL domain, by group and timepoint, derived from the regression. For all MSEL composite scores and domains tested, we found group-by-timepoint interactions for FXS and FH-ASD suggesting different longitudinal trajectories of development from control infants, as expected based on prior literature. Results will focus on the novel contrast presented herein: differences between FXS and FH-ASD throughout infancy. See Table 2 for interactions and Table 3 for interpretation of the size of the effects between groups at each timepoint.

**Table 2:** Regression model estimates of group on MSEL measures

	Estimate	SE	t-statistic	*df*	*p*-value	Cohen’s *d*
NVDQ						
FXS	-16.976	3.446	-4.927	52.642	< 0.001	-0.499
FH-ASD	-3.711	2.299	-1.614	106.451	0.11	-0.1548
FH-nonASD	-3.291	1.525	-2.158	836.362	0.031	-0.1912
Visit x FXS	-7.204	2.357	-3.056	120.012	0.003	-0.2912
Visit x FH-ASD	-7.862	1.612	-4.878	260.386	< 0.001	-0.4484
Visit x FH-nonASD	-1.117	1.11	-1.006	1084.541	0.315	-0.0623
VDQ						
FXS	-10.672	3.37	-3.167	498.394	0.002	-0.2848
FH-ASD	-2.97	2.396	-1.239	364.619	0.216	-0.1125
FH-nonASD	-3.175	1.689	-1.88	677.655	0.061	-0.1676
Visit x FXS	-21.113	2.482	-8.508	155.22	< 0.001	-0.7993
Visit x FH-ASD	-14.976	1.725	-8.682	242.928	< 0.001	-0.7996
Visit x FH-nonASD	-2.292	1.185	-1.935	1105.263	0.053	-0.1198
RL						
FXS	0.494	0.642	0.769	219	0.443	0.071
FH-ASD	0.563	0.433	1.301	925	0.193	0.115
FH-nonASD	-0.040	0.309	-0.130	1,131	0.896	-0.012
Visit x FXS	-5.657	0.565	-10.004	34	< 0.001	-1.063
Visit x FH-ASD	-3.852	0.336	-11.460	688	< 0.001	-1.021
Visit x FH-nonASD	-0.713	0.237	-3.007	1,370	0.003	-0.185
EL						
FXS	-0.191	0.625	-0.305	623	0.760	-0.027
FH-ASD	0.189	0.432	0.437	1,275	0.662	0.038
FH-nonASD	-0.120	0.312	-0.385	1,017	0.700	-0.034
Visit x FXS	-4.684	0.533	-8.786	64	< 0.001	-0.874
Visit x FH-ASD	-2.639	0.337	-7.842	871	< 0.001	-0.694
Visit x FH-nonASD	-0.618	0.239	-2.581	1,251	0.010	-0.159
FM						
FXS	-1.082	0.513	-2.110	25	0.045	-0.235
FH-ASD	-0.131	0.296	-0.443	315	0.658	-0.041
FH-nonASD	-0.111	0.211	-0.524	365	0.600	-0.048
Visit x FXS	-2.671	0.291	-9.184	1,071	< 0.001	-0.809
Visit x FH-ASD	-1.406	0.216	-6.509	232	< 0.001	-0.601
Visit x FH-nonASD	-0.532	0.149	-3.581	964	< 0.001	-0.222
GM						
FXS	-1.422	0.520	-2.736	114	0.007	-0.261
FH-ASD	-0.328	0.334	-0.983	1,439	0.326	-0.086
FH-nonASD	-0.262	0.248	-1.058	475	0.291	-0.095
Visit x FXS	-1.995	0.370	-5.394	118	-1.995	-0.515
Visit x FH-ASD	-1.040	0.242	-4.295	1,097	-1.040	-0.378
Visit x FH-nonASD	-0.225	0.175	-1.287	940	-0.225	-0.080
VR						
FXS	-0.560	0.563	-0.996	307	0.320	-0.091
FH-ASD	-0.042	0.388	-0.109	620	0.913	-0.010
FH-nonASD	-0.221	0.278	-0.796	652	0.426	-0.071
Visit x FXS	-3.920	0.462	-8.490	56	< 0.001	-0.856
Visit x FH-ASD	-2.225	0.287	-7.741	913	< 0.001	-0.685
Visit x FH-nonASD	-0.471	0.206	-2.293	1,111	0.022	-0.142

**Table 3:** Estimated marginal means contrasts for MSEL composite and raw scores

Group Contrast Estimates (SE)						
	FXS –FH-ASD	FXS – FH-nonASD	FXS – Control	FH-ASD – FH-nonASD	FH-ASD – Control	FH-nonASD – Control
**6-month Timepoint**						
NVDQ	-13.27 (3.91)	-13.69 (3.36)	-16.98 (3.45)	-0.42 (2.07)	-3.71 (2.30)	-3.29 (1.53)
VDQ	-7.70 (3.76)	-7.50 (3.29)	-10.67 (3.37)	0.21 (2.24)	-2.97 (2.40)	-3.18 (1.69)
RL	-0.06 (0.66)	0.55 (0.59)	0.46 (0.62)	0.61 (0.40)	0.51 (0.43)	-0.09 (0.31)
EL	-0.31 (0.71)	0.01 (0.64)	-0.17 (0.64)	0.31 (0.40)	0.14 (0.43)	-0.17 (0.31)
FM	-1.00 (0.48)	-1.02 (0.45)	-1.18 (0.45)	-0.02 (0.27)	-0.19 (0.29)	-0.16 (0.22)
GM	-1.12 (0.63)	-1.27 (0.58)	-1.58 (0.59)	-0.15 (0.32)	-0.45 (0.34)	-0.31 (0.24)
VR	-0.51 (0.63)	-0.36 (0.57)	-0.59 (0.58)	0.15 (0.37)	-0.08 (0.38)	-0.23 (0.28)
**12-month Timepoint**						
NVDQ	-12.61 (2.56)	-19.77 (2.33)	-24.18 (2.37)	-7.16 (1.37)	-11.57 (1.49)	-4.41 (1.02)
VDQ	-13.84 (2.53)	-26.32 (2.29)	-31.79 (2.33)	-12.48 (1.52)	-17.95 (1.63)	-5.47 (1.17)
RL	-1.88 (0.44)	-4.46 (0.40)	-5.24 (0.42)	-2.57 (0.25)	-3.36 (0.27)	-0.79 (0.19)
EL	-2.38 (0.46)	-4.11 (0.41)	-4.86 (0.43)	-1.73 (0.25	-2.49 (0.27)	-0.76 (0.20)
FM	-2.17 (0.31)	-3.08 (0.29)	-3.74 (0.29)	-0.91 (0.19)	-1.57 (0.20)	-0.66 (0.15)
GM	-2.17 (0.41)	-3.09 (0.38)	-3.61 (0.38)	-0.92 (0.21)	-1.44 (0.23)	-0.52 (0.17)
VR	-2.14 (0.41)	-3.74 (0.39)	-4.42 (0.39)	-1.59 (0.24)	-2.28 (0.26)	-0.69 (0.19)
**24-month Timepoint**						
NVDQ	-11.95 (3.37)	-25.86 (3.18)	-31.38 (3.23)	-13.91 (1.94)	-19.43 (2.09)	-5.53 (1.49)
VDQ	-19.98 (3.84)	-45.14 (3.33)	-52.90 (3.44)	-25.16 (2.17)	-32.92 (2.35)	-7.76 (1.65)
RL	-3.71 (0.72)	-9.47 (0.65)	-10.95 (0.68)	-5.75 (0.39)	-7.24 (0.43)	-1.48 (0.30)
EL	-4.45 (0.70)	-8.22 (0.63)	-9.56 (0.65)	-3.77 (0.40)	-5.12 (0.43)	-1.35 (0.31)
FM	-3.35 (0.48)	-5.14 (0.43)	-6.30 (0.44)	-1.80 (0.26)	-2.95 (0.28)	-1.16 (0.20)
GM	-3.21 (0.61)	-4.91 (0.59)	-5.64 (0.59)	-1.70 (0.32)	-2.43 (0.34)	-0.72 (0.24)
VR	-3.77 (0.60)	-7.11 (0.54)	-8.25 (0.56)	-3.34 (0.35)	-4.48 (0.38)	-1.14 (0.27)

### Trajectories of nonverbal developmental quotient

**Fig. 1:**
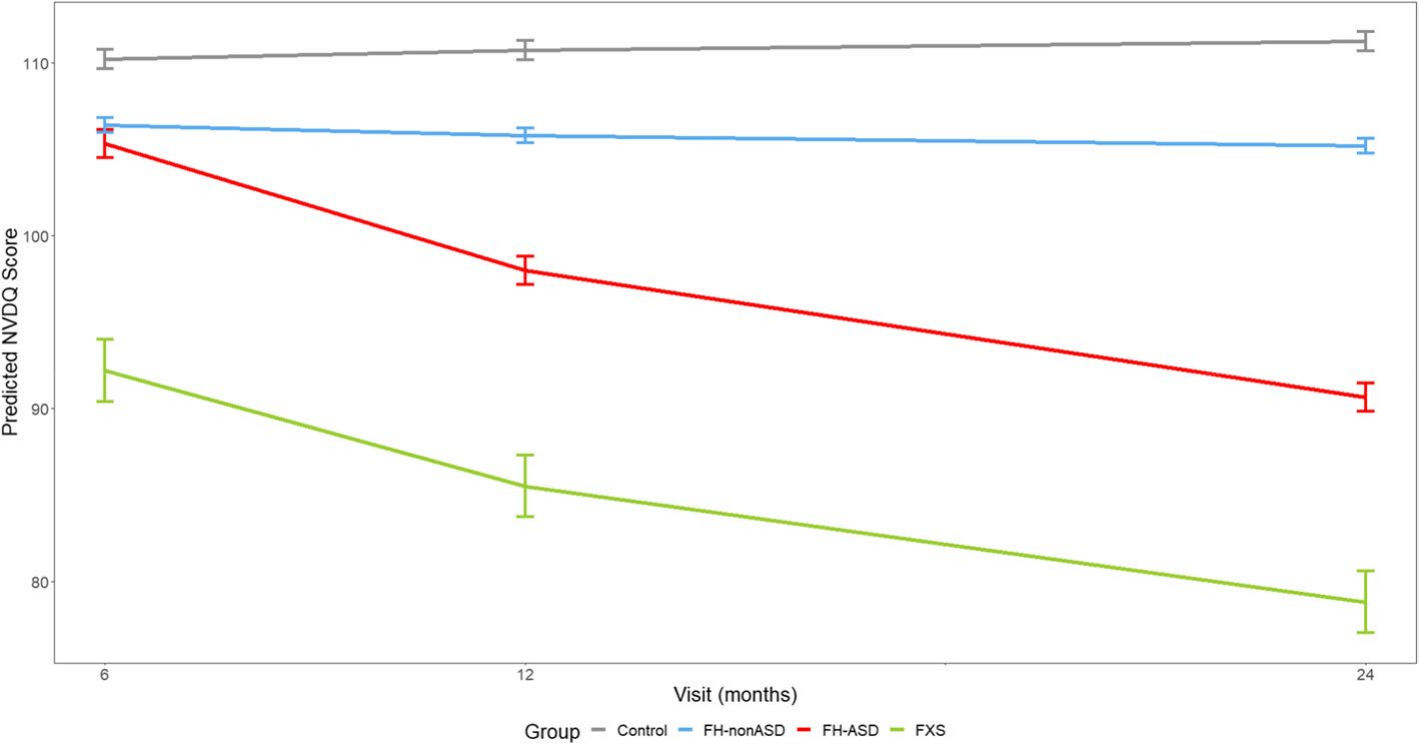
MSEL nonverbal developmental quotient score trajectory by group and timepoint. Model-generated differential trajectories of derived Nonverbal Developmental Quotient (NVDQ) from the MSEL by group and timepoint (Visit). The FXS group exhibited significantly delayed trajectories of NVDQ from all other groups from 6–24 months. By 12 months of age a pattern emerged between all groups (Table 3) that persisted through 24 months, such that control > FH-nonASD > FH-ASD > FXS

The FXS infants had significantly lower scores than FH-ASD infants throughout infancy on the NVDQ, aligning with findings with the ELC reported by Shen and colleagues [19]. FXS infants were delayed in comparison to control infants at 6 months and gaps in scores widened over time. The FH-ASD group’s NVDQ scores, on the other hand, did not differ from control infants until 12 and 24 months, and to a lesser extent than FXS. These findings corroborate the earlier onset of substantial cognitive differences from typical development for FXS infants, whereas FH-ASD infants’ delays emerge later in infancy and are less pronounced. Scores of FH-nonASD infants were intermediate between those of FH-ASD and control infants at 12 and 24 months on NVDQ (see Tables 2 and 3 and Fig. 1).

### Trajectories of verbal developmental quotient

**Fig. 2:**
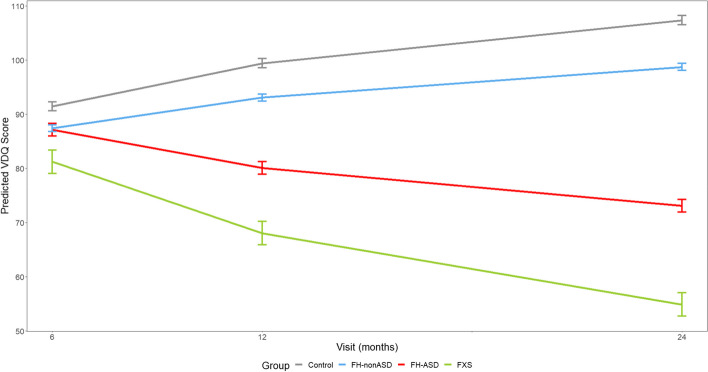
MSEL Verbal Developmental Quotient Score Trajectory by Group and Timepoint. Model-generated differential trajectories of derived Verbal Developmental Quotient (VDQ) from the MSEL by group and timepoint (Visit). The FXS group exhibited significantly delayed trajectories of VDQ from 6–24 months compared to controls. By 12 months of age a pattern emerged between groups (Table 3) that persisted through 24 months, such that control > FH-nonASD > FH-ASD > FXS

As with the NVDQ, FXS infants scored significantly lower than control infants starting at 6 months on the VDQ with gaps in performance widening at 12 and 24 months, whereas FH-ASD infants did not score lower than control infants until 12 and 24 months. FXS infants did not diverge from FH-ASD infants on VDQ as early as they did for NVDQ, with significant differences detectable at 12 and 24 months. The lack of divergence between FXS and FH-ASD at 6 months on VDQ may be at least in part attributable to similar age equivalent scores on RL in these groups, as described below. FH-nonASD VDQ scores were again intermediate between the FH-ASD and control groups at 12 and 24 months (see Tables 2 and 3 and Fig. 2).

### Domain-level skill trajectories

**Fig. 3:**
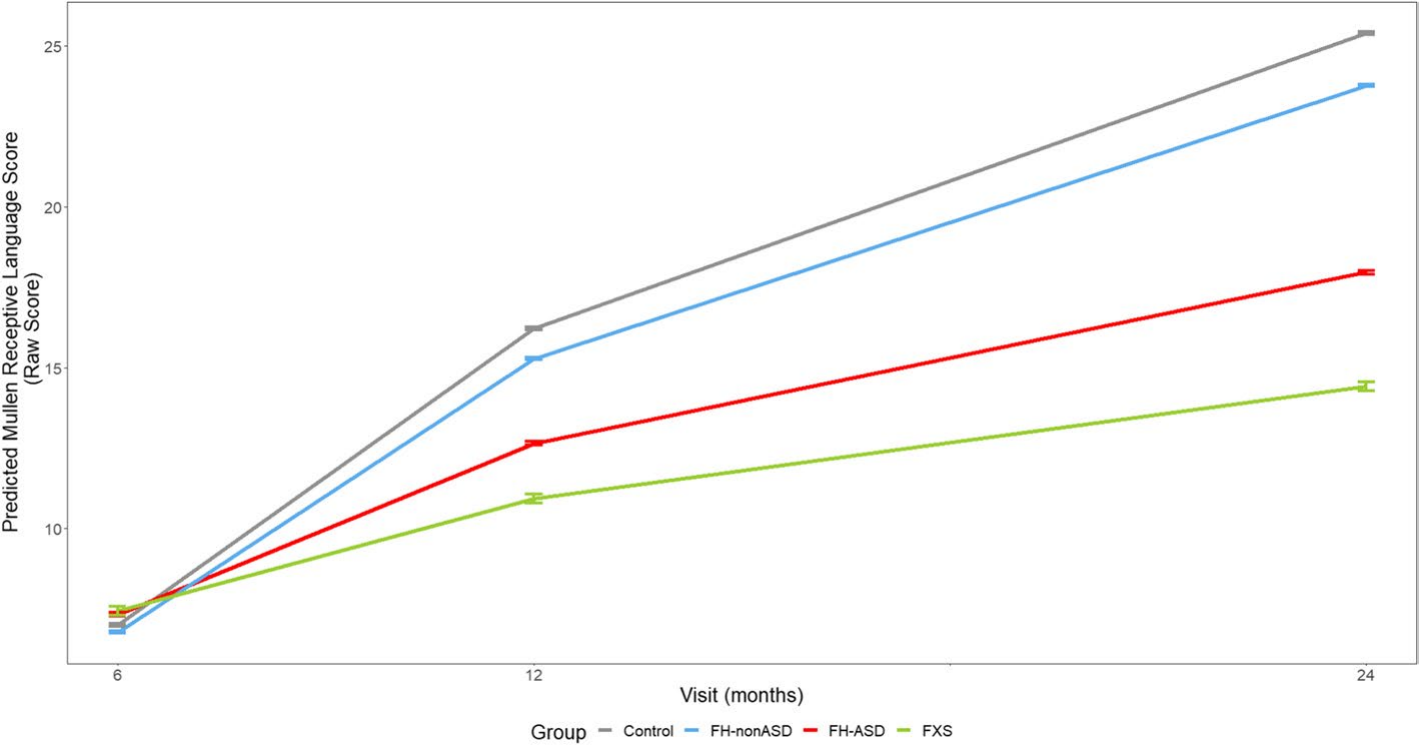
MSEL Receptive Language Score Trajectory by Group and Timepoint. Model-generated differential trajectories of Receptive Language from the MSEL by group and timepoint (Visit). At 6 months, no group trajectories significantly differed from one another. By 12 months of age a pattern emerged between groups (Table 3) that persisted through 24 months, such that control > FH-nonASD > FH-ASD > FXS

**Fig. 4:**
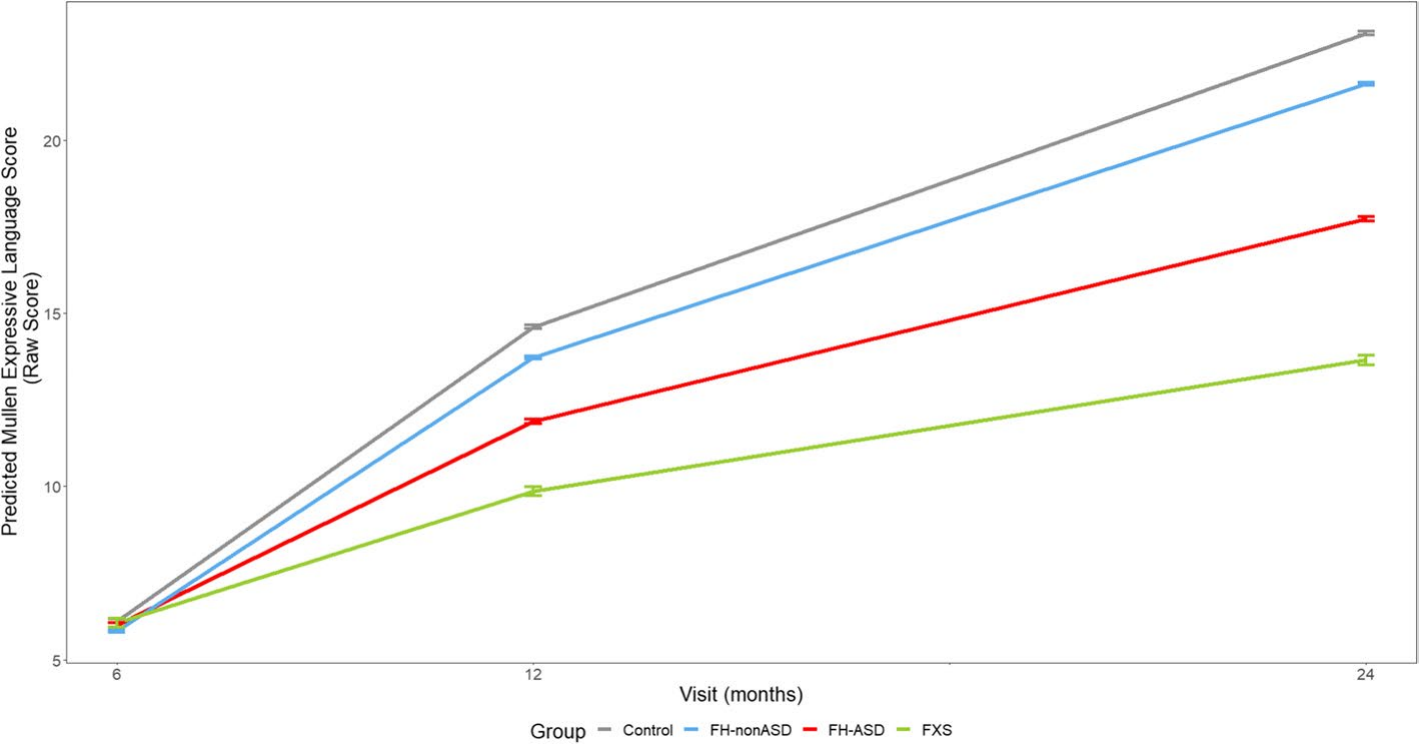
MSEL Expressive Language Score Trajectory by Group and Timepoint. Model-generated differential trajectories of Expressive Language from the MSEL by group and timepoint (Visit). At 6 months, no group trajectories significantly differed from one another. By 12 months of age a pattern emerged between groups (Table 3) that persisted through 24 months, such that control > FH-nonASD > FH-ASD > FXS

**Fig. 5:**
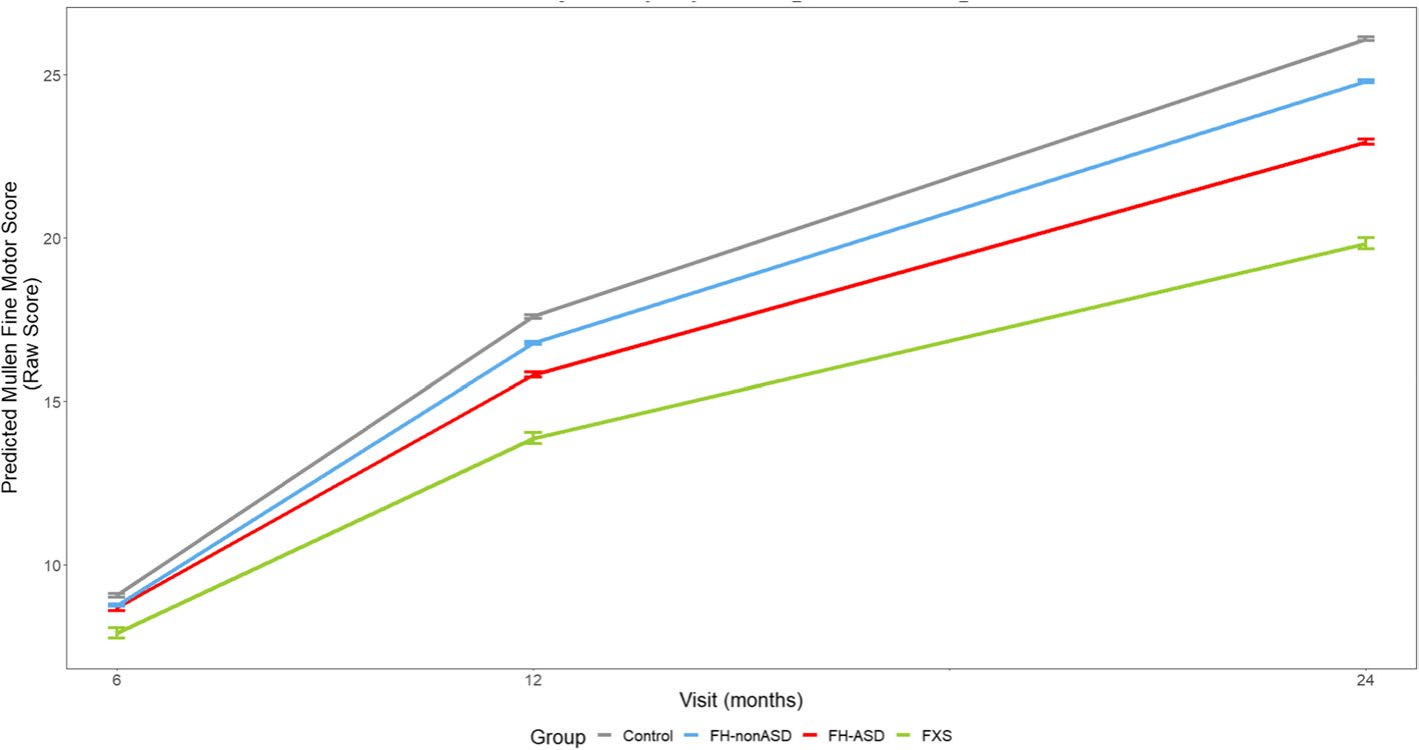
MSEL Fine Motor Score Trajectory by Group and Timepoint. Model-generated differential trajectories of Fine Motor from the MSEL by group and timepoint (Visit). At 6 months, no group trajectories significantly differed from one another. By 12 months of age a pattern emerged between groups (Table 3) that persisted through 24 months, such that control > FH-nonASD > FH-ASD > FXS

**Fig. 6:**
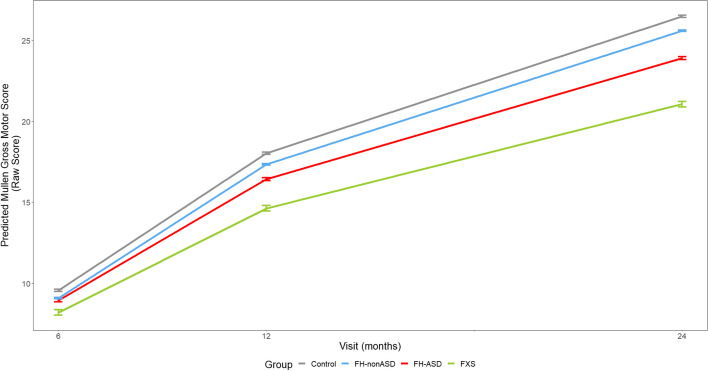
MSEL Gross Motor Score Trajectory by Group and Timepoint. Model-generated differential trajectories of Gross Motor from the MSEL by group and timepoint (Visit). At 6 months, no group trajectories significantly differed from one another. By 12 months of age a pattern emerged between groups (Table 3) that persisted through 24 months, such that control > FH-nonASD > FH-ASD > FXS

**Fig. 7:**
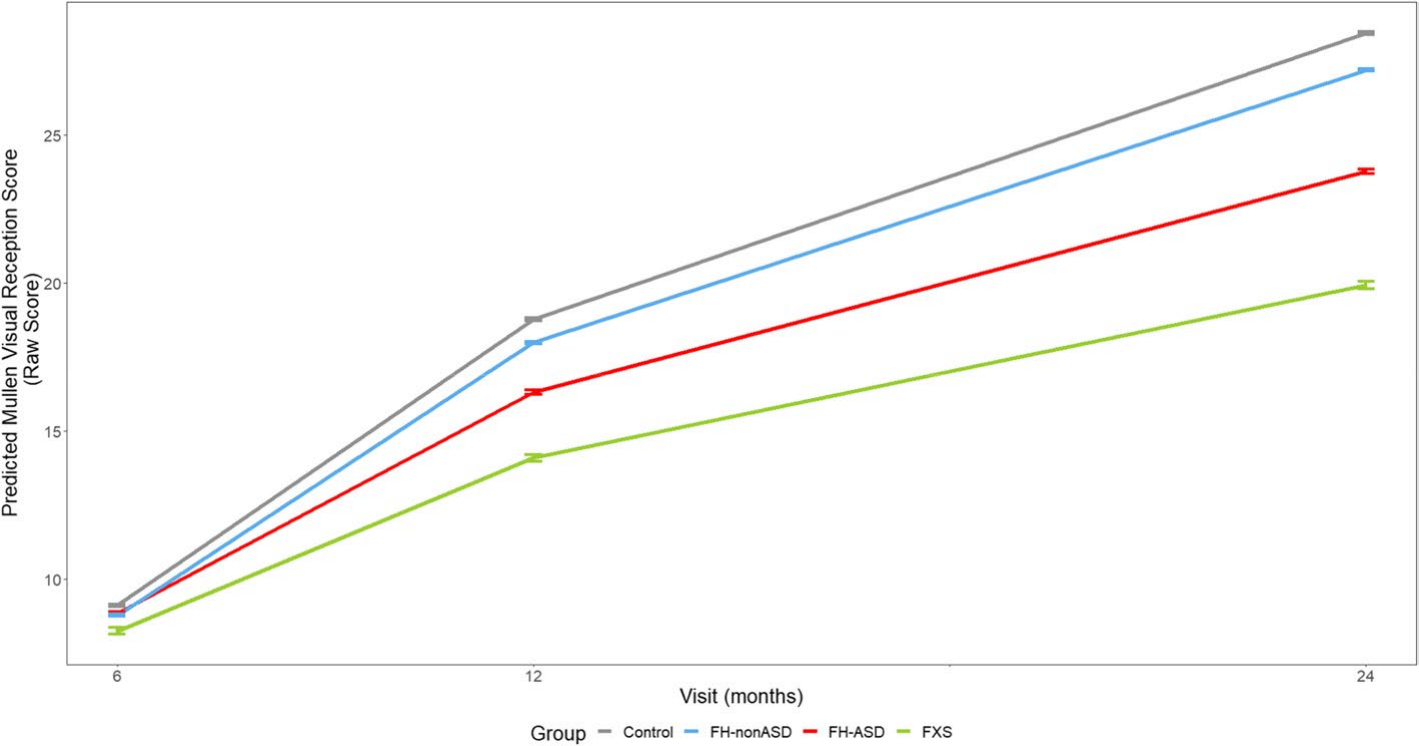
MSEL Visual Reception Score Trajectory by Group and Timepoint. Model-generated differential trajectories of Visual Reception from the MSEL by group and timepoint (Visit). At 6 months, no group trajectories significantly differed from one another. By 12 months of age a pattern emerged between groups (Table 3) that persisted through 24 months, such that control > FH-nonASD > FH-ASD > FXS

At 6 months, no groups scored significantly differently from one another on any individual MSEL domain. On all MSEL domains at 12 and 24 months, the infant groups demonstrated scores that resembled a successive pattern in which estimated means of FXS < FH-ASD < FH-nonASD < control (see Tables 2 and 3 and Figs. 3, 4, 5, 6 and 7).

### Supplemental results: influence of cognitive level on trajectories in ASD

FH-ASD infants with low cognitive level (FH-ASD-Low; 29% of the FH-ASD group) exhibited less developmental gains throughout infancy compared to FH-ASD infants with average or high cognitive level on all domains of the MSEL, such that they performed similarly to FXS infants on each MSEL measure at one or more timepoints. FH-ASD-Low infants scored similarly to FXS infants throughout infancy on both language domains and VR, and scored higher than FXS infants at 12 and 24 months on motor domains only. Composite scores between these groups showed that FH-ASD-Low infants have higher scores than FXS infants at 6 months, but over time make less gains and perform more similar to FXS infants, at 24 months for NVDQ and at 12 and 24 months for VDQ (see Tables S8 and S10 for regression results of raw and age equivalent scores, Tables S9 and S11 for group contrasts of raw and age equivalent scores, and Figures S18, S19, S20, S21, S22, S23 and S24 in the Supplement).

### Supplemental results: MSEL age equivalent scores

The analysis of MSEL age equivalent scores mirrored the divergence seen in raw scores on individual MSEL skill domains such that FXS < FH-ASD < FH-nonASD < control, except for RL, for which FXS and FH-ASD did not significantly differ from each other in infancy. This difference between age equivalent and raw score results can be attributed to the age equivalent scores having larger standard deviations in each group than the raw scores, limiting the ability to detect significant differences between the groups when both groups display very low scores on RL (see Tables S3 and S4 and Figs. S1, S2, S3, S4 and S5 in the Supplement for age equivalent score results).

### Supplemental results: males

The male-only infant analyses of both raw and age equivalent MSEL scores were consistent with the whole group (males and females combined) results (see Table S5 and S6 and Figs. S6, S7, S8, S9, S10, S11, S12, S13, S14, S15, S16 and S17 in the Supplement).

## Discussion

In the current study, we charted differential skill development in infancy on multiple domains between FXS, FH-ASD, FH-nonASD, and control infants, focusing on novel comparisons between FXS and FH-ASD. We report that FH-ASD infants’ performance was indistinguishable from typical development at 6 months of age, while FXS infants scored significantly below control infants at the 6-month timepoint on NVDQ and VDQ, reflecting the early global developmental delay characteristic of this group [26], and the slower onset of deficits in FH-ASD [8]. By 12 months of age, and continuing through 24 months of age, both FXS and FH-ASD infants scored significantly lower than controls, indicative of emerging deficits in FH-ASD and persisting deficits in FXS. Our results also demonstrate that the developmental divergence *between* FXS and FH-ASD is detectable within the first two years of life, with differences in NVDQ scores apparent between FXS and FH-ASD groups by 6 months of age, and differences in VDQ and all MSEL domains detectable by 12 months of age. These findings provide evidence for an early emergence of differential developmental time courses in FXS and FH-ASD infants, reflective of their distinct etiology.

At the level of individual domains – gross and fine motor, expressive and receptive language, and visual reception – all four infant groups are indistinguishable at the 6-month timepoint, with differences emerging by 12 months and persisting through 24 months. While all groups increased in scores over time, both FXS and FH-ASD infants made significantly fewer gains than control and FH-nonASD infants throughout infancy. By the 12-month timepoint, infants with FXS had MSEL scores significantly lower than those of FH-ASD infants, who were also scoring significantly lower than control and FH-nonASD infants. This graded effect persisted, and became more pronounced at two years of age. These patterns of results are consistent with reports of delayed trajectories in FH-ASD compared to controls across the second year of life [6, 8], and delayed trajectories in FXS that generally emerge sometime in the first year [20–27], though the exact timing of which has varied across studies.

The earliest group differences in our study were found on composite, and not domain-level, scores. Previous studies of FXS infants using the MSEL have found a similar pattern overall, in which composite measures show the earliest divergence for FXS infants versus controls, and domain-level differences showed variable patterns of timing of emergence within and across studies. For example, a study with a very large sample (*n* = 439) of FXS infants detected delays on the ELC at 6 months in both males and females, while only males exhibited delays compared to controls on specific MSEL domains at 6 months [20]. It appears that the ELC has an ability to detect very early differences in development in FXS, which individual domains may not (e.g., for females with FXS who have less pronounced deficits). Similarly, we found trending differences in our males-only analysis in which FXS infants scored lower than controls on the motor domains at 6 months (see online Supplement), though the effect did not reach statistical significance. Another study of FXS and control infants found differences across the ELC and all domains of the MSEL at the 6-month timepoint, with the largest effect size in ELC divergence, but the samples were very small at 6 months (*n* = 5 for FXS and *n* = 9 for controls) [25]. The lack of consensus for significant group differences for FXS from controls among 6-month findings could be a matter of differences in sample size and related statistical power, other implicit factors specific to the cohorts of FXS infants, or the score type used (e.g., raw, AE, standard scores). Composite measures of cognitive skill which incorporate scores from multiple individual domains, such as the NVDQ and the ELC, may be better able to detect more subtle FH-ASD and FXS group differences very early in infancy than individual domains which allow for less variability in scores due to small numbers of items for administration at 6 months. This may explain why FH-ASD and FXS differences were only detectable at 6 months on NVDQ in the current study and on ELC in Shen and colleagues’ study [19], but not on individual domains. Of all domains tested, FH-ASD exhibited similar levels of delay to FXS in language domains, and in particular RL. Low performance of FH-ASD infants on RL is consistent with prior findings from our group, where RL was the only MSEL domain to differentiate FH-ASD from FH-nonASD infants at 12 months [8]. This suggests that atypical RL development may be a common, early-emerging feature shared across FH-ASD and FXS. Taken together, findings from our study, and others comparing FXS and FH-ASD, demonstrate the potential utility of comparing these groups in infancy for illuminating the early biological and behavioral origins of different manifestations of cognitive delay, autistic features, and psychiatric behaviors [56], with potential implications for informing interventions.

To assess the generalizability of our findings and provide even more specific group contrasts than FH-ASD and FXS, we conducted analyses accounting for cognitive level in the FH-ASD group, which varies greatly in idiopathic autism, but is generally higher in familial ASD samples [57]. When accounting for cognitive level of the FH-ASD group, our observation of the presymptomatic period in FH-ASD remains evident; even infants who would later go on to exhibit cognitive delays by 24 months were indistinguishable from typical development on all MSEL domains and derived composites at 6 months. We also found that for the portion of FH-ASD infants who have lower cognitive level, language and visual reception skills are relatively more affected (to the point of similarity in scores to FXS) whereas motor skills are more preserved but still lower when compared to FH-ASD infants with typical cognitive levels.

### Limitations

FXS infant scores on specific MSEL domains were indistinguishable from those of FH-ASD infants at 6 months but differed at 12 months in the current study. Though 12 months is the earliest timepoint that we were able to detect differences, it is possible that these differences emerge earlier, sometime between 6 and 12 months, and potentially at different timepoints for each skill domain. Comparing these groups at more timepoints throughout infancy may offer additional insight beyond what the current study was able to report. Additionally, the lack of item availability on the MSEL for very young infants may limit the opportunity to detect significant group differences in the first 6 months of life. There are newer assessments used in ongoing longitudinal studies of infants (e.g., Bayley Scales of Infant and Toddler Development [58]) that may better capture a wider range of cognitive and developmental skills within the first few months of life. If these new assessments can capture more variability in younger infants, they may be better suited for analyses using standard scores, which we were unable to do in the current analysis due to floor effects on T-scores for the FXS group. Unveiling an even more granular level of longitudinal developmental differences between FXS and FH-ASD infants within the first year of life would further inform etiologically specific interventions.

Given the relationship between autistic behaviors and FXS, it is possible that many of our FXS participants developed social and repetitive behaviors indicative of ASD. However, given the limited data available on ASD outcomes at 24 months in our FXS group, we were unable to assess with confidence whether ASD traits influenced the reported developmental trajectories for the FXS participants. It should also be noted that the FH-ASD infants may constitute a distinct subset of idiopathic autism, and may differ from community samples in several ways [57]. Therefore, findings of differences between FXS infants and the FH-ASD group in this study cannot be extrapolated to the broader group of all infants who go on to develop ASD.

We do not know whether the observed differential skill trajectories between FXS and FH-ASD is applicable to females, due to low statistical power to conduct sex specific analyses. Larger samples of female FXS and FH-ASD infants within the same longitudinal study design would allow for examination of timing and extent of behavioral divergence throughout infancy, specific to female members of these groups who are known to develop differently than males [59, 60].

There is an underrepresentation of racial and ethnic diversity within our study sample, with most participants identifying as non-Hispanic White. Therefore, it is possible that our findings are not generalizable to all racial and ethnic groups and should be considered with this caveat. Current studies in our research group with similar designs have increased efforts to recruit participant samples that better represent a wide range of racial and ethnic diversity, as well as to include more females with these diagnoses. Future research that is more representative of all racial and ethnic categories will allow for more generalizable findings.

## Conclusions and future directions

We utilized longitudinal developmental assessments from infancy through toddlerhood to compare skill trajectories in FXS and FH-ASD. Our results demonstrate detectable group differences between FXS and FH-ASD by 6 months that persist through age 2. This highlights distinct trajectories in which FH-ASD exhibits a presymptomatic postnatal period that cannot be discerned from typical development, while FXS presents with the early onset of global developmental delay. This work builds upon evidence of divergent neural and cognitive development in infancy between FXS and FH-ASD and contributes to our understanding of important distinctions in the development and behavioral phenotype of these two groups. Our work revealing distinct trajectories of individual skills spanning language, motor, and visual reception throughout infancy in FXS and FH-ASD compared to typical development lends support to a growing interest in developing and evaluating cognitive interventions in the first years of life for infants with FXS and infants at high likelihood for ASD.

To expand our understanding of longitudinal development of individuals with FXS from infancy through school age, our group is currently collecting follow-up data on the FXS participants including their autistic traits, adaptive behavior, indicators of anxiety, and more. This will allow us to address questions in future work that we were unable to answer in the current study, related to whether cognitive trajectories in infancy for the FXS group differed dependent on variability in behaviors, including autistic traits. Additionally, describing the relationship between trajectories in infancy and school-age skills and developmental outcomes between FXS and FH-ASD is of interest and is a future goal of this research.

### Supplementary Information

**Supplementary Material 1.**

## Data Availability

The datasets analyzed in the current study are available in the National Database for Autism Research (NDAR) repository in collection #19 titled “Longitudinal MRI Study of Infants at Risk for Autism”.

## Abbreviations

ADI-R: Autism Diagnostic Interview-Revised
ADOS: Autism Diagnostic Observation Schedule–Generic
ASD: Autism spectrum disorder
DSM-IV-TR: Diagnostic and Statistical Manual of Mental Disorders, Edition four, Text Revision
DSM-5: Diagnostic and Statistical Manual of Mental Disorders, Edition five
EL: Expressive language
ELC: Early Learning Composite
FH: Familial history/older sibling with autism spectrum disorder
FH-ASD: Infants with an older sibling with autism spectrum disorder, who go on to develop autism themselves
FH-nonASD: Infants with an older sibling with autism spectrum disorder, who do not go on to develop autism themselves
FM: Fine motor
FMRP: Fragile X messenger ribonucleoprotein
FXS: Fragile X syndrome
GM: Gross motor
MRI: Magnetic resonance imaging
MSEL: Mullen Scales of Early Learning
NVDQ: Nonverbal developmental quotient
RL: Receptive language
VDQ: Verbal developmental quotient
VR: Visual reception
