# Supplementary material for: Differential cognitive and behavioral development from 6 to 24 months in autism and fragile X syndrome
Source: J Neurodev Disord. 2024 Mar 20;16:12. doi: 10.1186/s11689-024-09519-y (PMC10953146; doi:10.1186/s11689-024-09519-y)
Supplement: Supplementary file 1 — Supplementary Material 1. [file 11689_2024_9519_MOESM1_ESM.docx]

**TABLE OF CONTENTS**

1. Tables
   1. Extended Regression Table, MSEL Composite and Raw Scores, Table S1
   2. Unimputed Regression Results, MSEL Composite and Raw Scores, Table S2
   3. MSEL Age Equivalent Score Regression Model Estimates, Table S3
   4. MSEL Age Equivalent Score Estimated Marginal Means Contrasts, Table S4
   5. MSEL Raw Score Estimated Marginal Means Contrasts, Males Only, Table S5
   6. MSEL Age Equivalent Score Estimated Marginal Means Contrasts, Males Only, Table S6
   7. Maternal Education Comparison, Table S7
   8. FH-ASD Cognitive Level Group MSEL Raw Score Regression Results, Table S8
   9. FH-ASD Cognitive Level Group MSEL Raw Score Estimated Marginal Means Contrasts, Table S9
   10. FH-ASD Cognitive Level Group MSEL Composite and Age Equivalent Score Regression Results, Table S10
   11. FH-ASD Cognitive Level Group MSEL Composite and Age Equivalent Score Estimated Marginal Means Contrasts, Table S11
   12. 24-month ASD Data Availability for FXS Infants, Table S12
2. Figures
   1. MSEL Age Equivalent Score Trajectories, Figures S1-S5
   2. MSEL Composite and Age Equivalent Score Trajectories, Males Only, Figures S6-S12
   3. MSEL Raw Score Trajectories, Males Only, Figures S13-S17
   4. FH-ASD Cognitive Level Group Age Equivalent Score Trajectories, Figures S18-S24

**SUPPLEMENTAL RESULTS - TABLES**

Table S1. Regression Model Estimates of Group on MSEL Composite and Raw Scores, Extended

|  | Beta | SE | t-statistic | *df* | *p*-value | Cohen's *d* |
| --- | --- | --- | --- | --- | --- | --- |
| **NVDQ** | | | | | | |
| Intercept | 113.012 | 1.606 | 70.37 | 869.1 | < 0.001 | -- |
| Visit (Ordinal) | 0.514 | 0.887 | 0.579 | 1296.361 | 0.563 | 0.0356 |
| FXS | -16.976 | 3.446 | -4.927 | 52.642 | < 0.001 | -0.499 |
| FH-ASD | -3.711 | 2.299 | -1.614 | 106.451 | 0.11 | -0.1548 |
| FH-nonASD | -3.291 | 1.525 | -2.158 | 836.362 | 0.031 | -0.1912 |
| Male | -3.706 | 0.928 | -3.992 | 776.727 | < 0.001 | -0.2494 |
| UW Site | -0.886 | 1.319 | -0.672 | 1027.887 | 0.502 | -0.0417 |
| WU Site | -4.751 | 1.329 | -3.576 | 269.06 | < 0.001 | -0.2309 |
| UNC Site | 1.584 | 1.337 | 1.185 | 190.873 | 0.238 | 0.11 |
| Visit x FXS | -7.204 | 2.357 | -3.056 | 120.012 | 0.003 | -0.2912 |
| Visit x FH-ASD | -7.862 | 1.612 | -4.878 | 260.386 | < 0.001 | -0.4484 |
| Visit x FH-nonASD | -1.117 | 1.11 | -1.006 | 1084.541 | 0.315 | -0.0623 |
| **VDQ** | | | | | | |
| Intercept | 92.905 | 1.792 | 51.844 | 907.389 | < 0.001 | -- |
| Visit (Ordinal) | 7.955 | 0.951 | 8.369 | 1145.744 | < 0.001 | 0.5174 |
| FXS | -10.672 | 3.37 | -3.167 | 498.394 | 0.002 | -0.2848 |
| FH-ASD | -2.97 | 2.396 | -1.239 | 364.619 | 0.216 | -0.1125 |
| FH-nonASD | -3.175 | 1.689 | -1.88 | 677.655 | 0.061 | -0.1676 |
| Male | -3.254 | 1.06 | -3.07 | 711.215 | 0.002 | -0.1922 |
| UW Site | -2.103 | 1.504 | -1.398 | 998.421 | 0.162 | -0.0868 |
| WU Site | -4.487 | 1.477 | -3.039 | 581.387 | 0.002 | -0.1913 |
| UNC Site | 5.407 | 1.443 | 3.748 | 1036.312 | < 0.001 | 0.3303 |
| Visit x FXS | -21.113 | 2.482 | -8.508 | 155.22 | < 0.001 | -0.7993 |
| Visit x FH-ASD | -14.976 | 1.725 | -8.682 | 242.928 | < 0.001 | -0.7996 |
| Visit x FH-nonASD | -2.292 | 1.185 | -1.935 | 1105.263 | 0.053 | -0.1198 |
| **RL** | | | | | | |
| Intercept | 7.169 | 0.315 | 22.781 | 1,418 | < 0.001 | -- |
| Visit (Ordinal) | 9.173 | 0.190 | 48.216 | 1,398 | < 0.001 | 2.962 |
| FXS | 0.494 | 0.642 | 0.769 | 219 | 0.443 | 0.071 |
| FH-ASD | 0.563 | 0.433 | 1.301 | 925 | 0.193 | 0.115 |
| FH-nonASD | -0.040 | 0.309 | -0.130 | 1,131 | 0.896 | -0.012 |
| Male | -0.441 | 0.175 | -2.528 | 1,346 | 0.012 | -0.155 |
| UW Site | -0.024 | 0.249 | -0.095 | 1,422 | 0.924 | -0.006 |
| WU Site | -0.672 | 0.246 | -2.727 | 700 | 0.007 | -0.171 |
| UNC Site | 0.741 | 0.244 | 3.039 | 754 | 0.002 | 0.270 |
| Visit x FXS | -5.657 | 0.565 | -10.004 | 34 | < 0.001 | -1.063 |
| Visit x FH-ASD | -3.852 | 0.336 | -11.460 | 688 | < 0.001 | -1.021 |
| Visit x FH-nonASD | -0.713 | 0.237 | -3.007 | 1,370 | 0.003 | -0.185 |
| **EL** | | | | | | |
| Intercept | 6.278 | 0.317 | 19.810 | 1,432 | < 0.001 | -- |
| Visit (Ordinal) | 8.497 | 0.192 | 44.371 | 1,426 | < 0.001 | 2.724 |
| FXS | -0.191 | 0.625 | -0.305 | 623 | 0.760 | -0.027 |
| FH-ASD | 0.189 | 0.432 | 0.437 | 1,275 | 0.662 | 0.038 |
| FH-nonASD | -0.120 | 0.312 | -0.385 | 1,017 | 0.700 | -0.034 |
| Male | -0.595 | 0.177 | -3.372 | 1,192 | < 0.001 | -0.208 |
| UW Site | -0.310 | 0.252 | -1.231 | 1,312 | 0.219 | -0.076 |
| WU Site | -0.411 | 0.246 | -1.668 | 937 | 0.096 | -0.104 |
| UNC Site | 0.971 | 0.252 | 3.854 | 322 | < 0.001 | 0.351 |
| Visit x FXS | -4.684 | 0.533 | -8.786 | 64 | < 0.001 | -0.874 |
| Visit x FH-ASD | -2.639 | 0.337 | -7.842 | 871 | < 0.001 | -0.694 |
| Visit x FH-nonASD | -0.618 | 0.239 | -2.581 | 1,251 | 0.010 | -0.159 |
| **FM** | | | | | | |
| Intercept | 9.003 | 0.220 | 40.845 | 654 | < 0.001 | -- |
| Visit (Ordinal) | 8.519 | 0.118 | 72.425 | 1,528 | < 0.001 | 4.433 |
| FXS | -1.082 | 0.513 | -2.110 | 25 | 0.045 | -0.235 |
| FH-ASD | -0.131 | 0.296 | -0.443 | 315 | 0.658 | -0.041 |
| FH-nonASD | -0.111 | 0.211 | -0.524 | 365 | 0.600 | -0.048 |
| Male | -0.444 | 0.125 | -3.543 | 1,449 | < 0.001 | -0.217 |
| UW Site | 0.241 | 0.179 | 1.341 | 1,458 | 0.180 | 0.082 |
| WU Site | -0.362 | 0.182 | -1.993 | 310 | 0.047 | -0.128 |
| UNC Site | 0.995 | 0.177 | 5.634 | 599 | < 0.001 | 0.504 |
| Visit x FXS | -2.671 | 0.291 | -9.184 | 1,071 | < 0.001 | -0.809 |
| Visit x FH-ASD | -1.406 | 0.216 | -6.509 | 232 | < 0.001 | -0.601 |
| Visit x FH-nonASD | -0.532 | 0.149 | -3.581 | 964 | < 0.001 | -0.222 |
| **GM** | | | | | | |
| Intercept | 9.135 | 0.260 | 35.102 | 725 | < 0.001 | -- |
| Visit (Ordinal) | 8.459 | 0.139 | 60.704 | 1,215 | < 0.001 | 3.746 |
| FXS | -1.422 | 0.520 | -2.736 | 114 | 0.007 | -0.261 |
| FH-ASD | -0.328 | 0.334 | -0.983 | 1,439 | 0.326 | -0.086 |
| FH-nonASD | -0.262 | 0.248 | -1.058 | 475 | 0.291 | -0.095 |
| Male | -0.141 | 0.149 | -0.951 | 1,532 | 0.342 | -0.058 |
| UW Site | 0.315 | 0.230 | 1.372 | 150 | 0.172 | 0.091 |
| WU Site | -0.138 | 0.220 | -0.624 | 185 | 0.533 | -0.041 |
| UNC Site | 1.330 | 0.230 | 5.773 | 76 | < 0.001 | 0.567 |
| Visit x FXS | -1.995 | 0.370 | -5.394 | 118 | < 0.001 | -0.515 |
| Visit x FH-ASD | -1.040 | 0.242 | -4.295 | 1,097 | < 0.001 | -0.378 |
| Visit x FH-nonASD | -0.225 | 0.175 | -1.287 | 940 | 0.199 | -0.080 |
| **VR** | | | | | | |
| Intercept | 9.531 | 0.284 | 33.544 | 1,346 | < 0.001 | -- |
| Visit (Ordinal) | 9.662 | 0.165 | 58.652 | 1,202 | < 0.001 | 3.623 |
| FXS | -0.560 | 0.563 | -0.996 | 307 | 0.320 | -0.091 |
| FH-ASD | -0.042 | 0.388 | -0.109 | 620 | 0.913 | -0.010 |
| FH-nonASD | -0.221 | 0.278 | -0.796 | 652 | 0.426 | -0.071 |
| Male | -0.558 | 0.161 | -3.473 | 1,492 | < 0.001 | -0.213 |
| UW Site | 0.053 | 0.232 | 0.230 | 1,072 | 0.819 | 0.014 |
| WU Site | -0.873 | 0.226 | -3.865 | 910 | < 0.001 | -0.241 |
| UNC Site | 0.228 | 0.232 | 0.980 | 274 | 0.328 | 0.090 |
| Visit x FXS | -3.920 | 0.462 | -8.490 | 56 | < 0.001 | -0.856 |
| Visit x FH-ASD | -2.225 | 0.287 | -7.741 | 913 | < 0.001 | -0.685 |
| Visit x FH-nonASD | -0.471 | 0.206 | -2.293 | 1,111 | 0.022 | -0.142 |

Table S2. Regression Model Estimates of Group on MSEL Composite and Raw Scores, Unimputed Results

|  | Beta | SE | t-statistic | *df* | *p*-value | Cohen's *d* |
| --- | --- | --- | --- | --- | --- | --- |
| **NVDQ** | | | | | | |
| Intercept | 113.627 | 1.600 | 71.007 | 928 | < 0.001 | -- |
| Visit (Ordinal) | 0.766 | 0.853 | 0.898 | 928 | 0.369 | 0.059 |
| FXS | -25.096 | 3.431 | -7.315 | 534 | < 0.001 | -0.633 |
| FH-ASD | -5.463 | 2.194 | -2.490 | 534 | 0.013 | -0.216 |
| FH-nonASD | -4.429 | 1.534 | -2.887 | 534 | 0.004 | -0.250 |
| Male | -4.200 | 0.941 | -4.465 | 534 | < 0.001 | -0.293 |
| UW Site | -0.848 | 1.336 | -0.635 | 534 | 0.526 | -0.042 |
| WU Site | -5.190 | 1.307 | -3.972 | 534 | < 0.001 | -0.261 |
| UNC Site | 1.801 | 1.291 | 1.395 | 534 | 0.164 | 0.121 |
| Visit x FXS | -7.244 | 2.472 | -2.930 | 928 | 0.003 | -0.254 |
| Visit x FH-ASD | -7.095 | 1.523 | -4.658 | 928 | < 0.001 | -0.403 |
| Visit x FH-nonASD | -0.715 | 1.075 | -0.665 | 928 | 0.506 | -0.044 |
| **VDQ** | | | | | | |
| Intercept | 93.562 | 1.759 | 53.176 | 926 | < 0.001 | -- |
| Visit (Ordinal) | 8.428 | 0.896 | 9.403 | 926 | < 0.001 | 0.618 |
| FXS | -18.147 | 3.717 | -4.882 | 534 | < 0.001 | -0.422 |
| FH-ASD | -4.864 | 2.376 | -2.047 | 534 | 0.041 | -0.177 |
| FH-nonASD | -5.102 | 1.663 | -3.068 | 534 | 0.002 | -0.266 |
| Male | -3.350 | 1.052 | -3.185 | 534 | 0.002 | -0.209 |
| UW Site | -2.483 | 1.495 | -1.661 | 534 | 0.097 | -0.109 |
| WU Site | -5.378 | 1.462 | -3.679 | 534 | < 0.001 | -0.242 |
| UNC Site | 5.919 | 1.444 | 4.099 | 534 | < 0.001 | 0.355 |
| Visit x FXS | -18.471 | 2.619 | -7.052 | 926 | < 0.001 | -0.610 |
| Visit x FH-ASD | -14.141 | 1.606 | -8.808 | 926 | < 0.001 | -0.762 |
| Visit x FH-nonASD | -1.410 | 1.131 | -1.247 | 926 | 0.213 | -0.082 |
| **RL** | | | | | | |
| Intercept | 7.270 | 0.313 | 23.241 | 926 | < 0.001 | -- |
| Visit (Ordinal) | 9.273 | 0.186 | 49.780 | 926 | < 0.001 | 3.272 |
| FXS | -0.061 | 0.694 | -0.089 | 534 | 0.929 | -0.008 |
| FH-ASD | 0.292 | 0.446 | 0.656 | 534 | 0.512 | 0.057 |
| FH-nonASD | -0.433 | 0.311 | -1.390 | 534 | 0.165 | -0.120 |
| Male | -0.500 | 0.174 | -2.866 | 534 | 0.004 | -0.188 |
| UW Site | -0.041 | 0.247 | -0.164 | 534 | 0.869 | -0.011 |
| WU Site | -0.819 | 0.243 | -3.374 | 534 | < 0.001 | -0.222 |
| UNC Site | 0.831 | 0.240 | 3.470 | 534 | < 0.001 | 0.300 |
| Visit x FXS | -5.779 | 0.528 | -10.949 | 926 | < 0.001 | -0.948 |
| Visit x FH-ASD | -3.787 | 0.332 | -11.395 | 926 | < 0.001 | -0.986 |
| Visit x FH-nonASD | -0.533 | 0.235 | -2.272 | 926 | 0.023 | -0.149 |
| **EL** | | | | | | |
| Intercept | 6.391 | 0.326 | 19.602 | 926 | < 0.001 | -- |
| Visit (Ordinal) | 8.568 | 0.184 | 46.589 | 926 | < 0.001 | 3.062 |
| FXS | -0.719 | 0.711 | -1.011 | 534 | 0.312 | -0.088 |
| FH-ASD | -0.046 | 0.456 | -0.102 | 534 | 0.919 | -0.009 |
| FH-nonASD | -0.422 | 0.318 | -1.325 | 534 | 0.186 | -0.115 |
| Male | -0.660 | 0.187 | -3.531 | 534 | < 0.001 | -0.232 |
| UW Site | -0.324 | 0.265 | -1.221 | 534 | 0.223 | -0.080 |
| WU Site | -0.512 | 0.260 | -1.970 | 534 | 0.049 | -0.129 |
| UNC Site | 1.081 | 0.257 | 4.212 | 534 | < 0.001 | 0.365 |
| Visit x FXS | -4.776 | 0.527 | -9.063 | 926 | < 0.001 | -0.784 |
| Visit x FH-ASD | -2.543 | 0.329 | -7.738 | 926 | < 0.001 | -0.670 |
| Visit x FH-nonASD | -0.478 | 0.232 | -2.064 | 926 | 0.039 | -0.136 |
| **FM** | | | | | | |
| Intercept | 9.055 | 0.218 | 41.510 | 928 | < 0.001 | -- |
| Visit (Ordinal) | 8.530 | 0.112 | 76.050 | 928 | < 0.001 | 4.993 |
| FXS | -1.949 | 0.462 | -4.216 | 534 | < 0.001 | -0.365 |
| FH-ASD | -0.227 | 0.295 | -0.768 | 534 | 0.443 | -0.066 |
| FH-nonASD | -0.271 | 0.207 | -1.312 | 534 | 0.190 | -0.114 |
| Male | -0.515 | 0.130 | -3.960 | 534 | < 0.001 | -0.260 |
| UW Site | 0.284 | 0.185 | 1.538 | 534 | 0.125 | 0.101 |
| WU Site | -0.410 | 0.181 | -2.273 | 534 | 0.023 | -0.149 |
| UNC Site | 1.142 | 0.178 | 6.401 | 534 | < 0.001 | 0.554 |
| Visit x FXS | -2.654 | 0.327 | -8.108 | 928 | < 0.001 | -0.702 |
| Visit x FH-ASD | -1.386 | 0.200 | -6.919 | 928 | < 0.001 | -0.599 |
| Visit x FH-nonASD | -0.458 | 0.141 | -3.236 | 928 | 0.001 | -0.212 |
| **GM** | | | | | | |
| Intercept | 9.177 | 0.263 | 34.927 | 911 | < 0.001 | -- |
| Visit (Ordinal) | 8.461 | 0.130 | 64.908 | 911 | < 0.001 | 4.301 |
| FXS | -2.641 | 0.549 | -4.814 | 534 | < 0.001 | -0.417 |
| FH-ASD | -0.501 | 0.351 | -1.428 | 534 | 0.154 | -0.124 |
| FH-nonASD | -0.495 | 0.246 | -2.017 | 534 | 0.044 | -0.175 |
| Male | -0.170 | 0.159 | -1.069 | 534 | 0.285 | -0.071 |
| UW Site | 0.360 | 0.227 | 1.587 | 534 | 0.113 | 0.105 |
| WU Site | -0.178 | 0.222 | -0.804 | 534 | 0.422 | -0.053 |
| UNC Site | 1.494 | 0.219 | 6.828 | 534 | < 0.001 | 0.591 |
| Visit x FXS | -1.867 | 0.381 | -4.901 | 911 | < 0.001 | -0.424 |
| Visit x FH-ASD | -1.000 | 0.235 | -4.260 | 911 | < 0.001 | -0.369 |
| Visit x FH-nonASD | -0.110 | 0.165 | -0.671 | 911 | 0.503 | -0.044 |
| **VR** | | | | | | |
| Intercept | 9.611 | 0.293 | 32.796 | 928 | < 0.001 | -- |
| Visit (Ordinal) | 9.706 | 0.159 | 61.050 | 928 | < 0.001 | 4.008 |
| FXS | -1.383 | 0.632 | -2.189 | 534 | 0.029 | -0.189 |
| FH-ASD | -0.187 | 0.404 | -0.462 | 534 | 0.644 | -0.040 |
| FH-nonASD | -0.455 | 0.283 | -1.611 | 534 | 0.108 | -0.139 |
| Male | -0.629 | 0.171 | -3.677 | 534 | < 0.001 | -0.241 |
| UW Site | 0.090 | 0.243 | 0.372 | 534 | 0.710 | 0.024 |
| WU Site | -0.959 | 0.238 | -4.035 | 534 | < 0.001 | -0.265 |
| UNC Site | 0.288 | 0.235 | 1.227 | 534 | 0.220 | 0.106 |
| Visit x FXS | -4.011 | 0.459 | -8.736 | 928 | < 0.001 | -0.756 |
| Visit x FH-ASD | -2.187 | 0.284 | -7.711 | 928 | < 0.001 | -0.667 |
| Visit x FH-nonASD | -0.361 | 0.200 | -1.802 | 928 | 0.072 | -0.118 |

Table S3. Regression Model Estimates of Group on MSEL Age Equivalent Scores

|  | Estimate | SE | t-statistic | *df* | *p*-value | Cohen's *d* |
| --- | --- | --- | --- | --- | --- | --- |
| NVDQ | | | | | | |
| FXS | -16.976 | 3.446 | -4.927 | 52.642 | < 0.001 | -0.499 |
| FH-ASD | -3.711 | 2.299 | -1.614 | 106.451 | 0.11 | -0.1548 |
| FH-nonASD | -3.291 | 1.525 | -2.158 | 836.362 | 0.031 | -0.1912 |
| Visit x FXS | -7.204 | 2.357 | -3.056 | 120.012 | 0.003 | -0.2912 |
| Visit x FH-ASD | -7.862 | 1.612 | -4.878 | 260.386 | < 0.001 | -0.4484 |
| Visit x FH-nonASD | -1.117 | 1.11 | -1.006 | 1084.541 | 0.315 | -0.0623 |
| VDQ | | | | | | |
| FXS | -10.672 | 3.37 | -3.167 | 498.394 | 0.002 | -0.2848 |
| FH-ASD | -2.97 | 2.396 | -1.239 | 364.619 | 0.216 | -0.1125 |
| FH-nonASD | -3.175 | 1.689 | -1.88 | 677.655 | 0.061 | -0.1676 |
| Visit x FXS | -21.113 | 2.482 | -8.508 | 155.22 | < 0.001 | -0.7993 |
| Visit x FH-ASD | -14.976 | 1.725 | -8.682 | 242.928 | < 0.001 | -0.7996 |
| Visit x FH-nonASD | -2.292 | 1.185 | -1.935 | 1105.263 | 0.053 | -0.1198 |
| RL | | | | | | |
| FXS | 0.232 | 0.756 | 0.307 | 556.185 | 0.759 | 0.0275 |
| FH-ASD | 0.786 | 0.545 | 1.442 | 279.464 | 0.15 | 0.132 |
| FH-nonASD | -0.098 | 0.398 | -0.247 | 183.318 | 0.805 | -0.0231 |
| Visit x FXS | -5.784 | 0.612 | -9.454 | 135.437 | < 0.001 | -0.8945 |
| Visit x FH-ASD | -4.803 | 0.42 | -11.429 | 277.155 | < 0.001 | -1.0473 |
| Visit x FH-nonASD | -0.963 | 0.299 | -3.218 | 366.347 | 0.001 | -0.2054 |
| EL | | | | | | |
| FXS | -0.35 | 0.765 | -0.458 | 207.861 | 0.648 | -0.0426 |
| FH-ASD | 0.339 | 0.515 | 0.659 | 872.648 | 0.51 | 0.0583 |
| FH-nonASD | -0.141 | 0.371 | -0.38 | 730.885 | 0.704 | -0.0339 |
| Visit x FXS | -4.843 | 0.59 | -8.21 | 179.026 | < 0.001 | -0.766 |
| Visit x FH-ASD | -3.254 | 0.392 | -8.307 | 1382.646 | < 0.001 | -0.726 |
| Visit x FH-nonASD | -0.766 | 0.285 | -2.687 | 875.34 | 0.007 | -0.1673 |
| FM | | | | | | |
| FXS | -1.264 | 0.465 | -2.718 | 1279.802 | 0.007 | -0.2381 |
| FH-ASD | 0.076 | 0.337 | 0.224 | 471.383 | 0.823 | 0.02 |
| FH-nonASD | -0.067 | 0.247 | -0.27 | 244.559 | 0.787 | -0.0249 |
| Visit x FXS | -2.773 | 0.384 | -7.231 | 80.632 | < 0.001 | -0.7077 |
| Visit x FH-ASD | -1.871 | 0.244 | -7.668 | 1241.422 | < 0.001 | -0.6728 |
| Visit x FH-nonASD | -0.687 | 0.179 | -3.848 | 630.551 | < 0.001 | -0.2418 |
| GM | | | | | | |
| FXS | -1.641 | 0.554 | -2.964 | 521.764 | 0.003 | -0.2661 |
| FH-ASD | -0.224 | 0.396 | -0.565 | 333.27 | 0.572 | -0.0516 |
| FH-nonASD | -0.216 | 0.281 | -0.771 | 494.052 | 0.441 | -0.0693 |
| Visit x FXS | -2.143 | 0.428 | -5.002 | 198.928 | < 0.001 | -0.4649 |
| Visit x FH-ASD | -1.507 | 0.289 | -5.208 | 895.075 | < 0.001 | -0.4608 |
| Visit x FH-nonASD | -0.37 | 0.211 | -1.757 | 560.685 | 0.079 | -0.1107 |
| VR | | | | | | |
| FXS | -0.328 | 0.65 | -0.505 | 987.532 | 0.614 | -0.0445 |
| FH-ASD | 0.46 | 0.471 | 0.978 | 370.574 | 0.329 | 0.0886 |
| FH-nonASD | -0.04 | 0.334 | -0.12 | 528.291 | 0.904 | -0.0109 |
| Visit x FXS | -4.058 | 0.582 | -6.97 | 43.777 | < 0.001 | -0.72 |
| Visit x FH-ASD | -2.968 | 0.353 | -8.417 | 1044.246 | < 0.001 | -0.742 |
| Visit x FH-nonASD | -0.723 | 0.254 | -2.841 | 900.592 | 0.005 | -0.1768 |

| Group Contrast Estimates (Standard Error) | | | | | | |
| --- | --- | --- | --- | --- | --- | --- |
|  | **FXS – FH-ASD** | **FXS – FH-nonASD** | **FXS – Control** | **FH-ASD – FH-nonASD** | **FH-ASD – Control** | **FH-nonASD – Control** |
| **6-month Timepoint** | | | | | | |
| RL | -0.55 (0.83) | 0.33 (0.72) | 0.23 (0.76) | 0.88 (0.50) | 0.79 (0.55) | -0.10 (0.40) |
| EL | -0.69 (0.80) | -0.21 (0.74) | -0.35 (0.77) | 0.48 (0.49) | 0.34 (0.52) | -0.14 (0.37) |
| FM | -1.34 (0.52) | -1.20 (0.45) | -1.26 (0.47) | 0.14 (0.33) | 0.08 (0.34) | -0.07 (0.25) |
| GM | -1.42 (0.59) | -1.43 (0.53) | -1.64 (0.55) | -0.01 (0.37) | -0.22 (0.40) | -0.22 (0.28) |
| VR | -0.79 (0.72) | -0.29 (0.64) | -0.33 (0.65) | 0.50 (0.42) | 0.46 (0.47) | -0.04 (0.33) |
| **12-month Timepoint** | | | | | | |
| RL | -1.54 (0.54) | -4.49 (0.49) | -5.55 (0.52) | -2.96 (0.32) | -4.02 (0.34) | -1.06 (0.25) |
| EL | -2.28 (0.51) | -4.29 (0.45) | -5.19 (0.47) | -2.01 (0.31) | -2.92 (0.33) | -0.91 (0.23) |
| FM | -2.24 (0.37) | -3.28 (0.35) | -4.04 (0.36) | -1.04 (0.21) | -1.80 (0.22) | -0.75 (0.16) |
| GM | -2.05 (0.40) | -3.20 (0.37) | -3.78 (0.39) | -1.15 (0.24) | -1.73 (0.26) | -0.59 (0.18) |
| VR | -1.88 (0.49) | -3.62 (0.46) | -4.39 (0.47) | -1.74 (0.27) | -2.51 (0.30) | -0.76 (0.21) |
| **24-month Timepoint** | | | | | | |
| RL | -2.52 (0.95) | -9.31 (0.85) | -11.34 (0.85) | -6.80 (0.48) | -8.82 (0.54) | -2.02 (0.38) |
| EL | -3.87 (0.84) | -8.36 (0.74) | -10.04 (0.75) | -4.50 (0.47) | -6.17 (0.52) | -1.67 (0.37) |
| FM | -3.14 (0.60) | -5.37 (0.56) | -6.81 (0.58) | -2.23 (0.30) | -3.67 (0.33) | -1.44 (0.23) |
| GM | -2.69 (0.63) | -4.97 (0.58) | -5.93 (0.60) | -2.29 (0.35) | -3.24 (0.38) | -0.96 (0.27) |
| VR | -2.97 (0.86) | -6.96 (0.83) | -8.44 (0.85) | -3.99 (0.42) | -5.48 (0.46) | -1.49 (0.32) |

Table S4. Estimated Marginal Means Contrasts for MSEL Age Equivalent Scores

Table S5. Estimated Marginal Means Contrasts for MSEL Raw Scores, Males Only

| Group Contrast Estimates (Standard Error) | | | | | | |
| --- | --- | --- | --- | --- | --- | --- |
|  | **FXS –FH-ASD** | **FXS –**  **FH-nonASD** | **FXS – Control** | **FH-ASD –**  **FH-nonASD** | **FH-ASD – Control** | **FH-nonASD – Control** |
| **6-month Timepoint** | | | | | | |
| RL | -0.36 (0.77) | -0.02 (0.70) | 0.01 (0.71) | 0.35 (0.49) | 0.38 (0.53) | 0.03 (0.42) |
| EL | -0.41 (0.73) | -0.43 (0.68) | -0.45 (0.70) | -0.02 (0.48) | -0.04 (0.51) | -0.01 (0.43) |
| FM | -1.28 (0.55) | -1.32 (0.50) | -1.32 (0.51) | -0.04 (0.31) | -0.04 (0.33) | -0.00 (0.27) |
| GM | -1.78 (0.64) | -1.70 (0.59) | -1.79 (0.61) | 0.08 (0.41) | -0.02 (0.42) | -0.09 (0.31) |
| VR | -0.70 (0.65) | -0.60 (0.60) | -0.82 (0.63) | 0.10 (0.40) | -0.12 (0.44) | -0.22 (0.36) |
| **12-month Timepoint** | | | | | | |
| RL | -2.28 (0.62) | -4.96 (0.56) | -5.56 (0.56) | -2.68 (0.31) | -3.29 (0.34) | -0.60 (0.27) |
| EL | -2.89 (0.65) | -4.70 (0.59) | -5.32 (0.61) | -1.81 (0.30) | -2.42 (0.32) | -0.62 (0.28) |
| FM | -2.66 (0.36) | -3.51 (0.33) | -4.11 (0.34) | -0.84 (0.21) | -1.45 (0.23) | -0.60 (0.19) |
| GM | -2.83 (0.51) | -3.68 (0.48) | -3.92 (0.49) | -0.85 (0.25) | -1.09 (0.27) | -0.24 (0.21) |
| VR | -2.63 (0.58) | -4.19 (0.54) | -4.61 (0.55) | -1.55 (0.26) | -1.97 (0.29) | -0.42 (0.24) |
| **24-month Timepoint** | | | | | | |
| RL | -4.19 (1.23) | -9.90 (1.11) | -11.14 (1.07) | -5.71 (0.48) | -6.95 (0.54) | -1.24 (0.42) |
| EL | -5.38 (1.17) | -8.97 (1.10) | -10.19 (1.05) | -3.59 (0.47) | -4.81 (0.52) | -1.22 (0.42) |
| FM | -4.05 (0.62) | -5.70 (0.58) | -6.90 (0.56) | -1.65 (0.30) | -2.85 (0.34) | -1.20 (0.27) |
| GM | -3.88 (0.73) | -5.66 (0.68) | -6.05 (0.67) | -1.78 (0.35) | -2.17 (0.40) | -0.39 (0.31) |
| VR | -4.57 (1.01) | -7.77 (0.95) | -8.40 (0.94) | -3.20 (0.40) | -3.83 (0.50) | -0.63 (0.37) |

| Group Contrast Estimates (Standard Error) | | | | | | |
| --- | --- | --- | --- | --- | --- | --- |
|  | **FXS –FH-ASD** | **FXS –**  **FH-nonASD** | **FXS – Control** | **FH-ASD –**  **FH-nonASD** | **FH-ASD – Control** | **FH-nonASD – Control** |
| **6-month Timepoint** | | | | | | |
| NVDQ | -14.78 (3.89) | -17.84 (3.37) | -18.95 (3.77) | -3.06 (2.67) | -4.17 (2.60) | -1.11 (2.27) |
| VDQ | -9.46 (4.01) | -12.39 (3.93) | -13.89 (3.86) | -2.93 (2.83) | -4.44 (2.86) | -1.50 (2.31) |
| RL | -0.57 (0.91) | -0.02 (0.86) | -0.04 (0.92) | 0.55 (0.58) | 0.53 (0.64) | -0.02 (0.51) |
| EL | -0.71 (0.93) | -0.59 (0.88) | -0.68 (0.92) | 0.12 (0.55) | 0.03 (0.62) | -0.09 (0.49) |
| FM | -1.27 (0.59) | -1.38 (0.51) | -1.30 (0.55) | -0.11 (0.39) | -0.03 (0.38) | 0.08 (0.33) |
| GM | -1.68 (0.75) | -1.69 (0.69) | -1.67 (0.70) | -0.01 (0.40) | 0.01 (0.44) | 0.02 (0.37) |
| VR | -0.50 (0.80) | -0.36 (0.74) | -0.42 (0.78) | 0.14 (0.48) | 0.08 (0.52) | -0.06 (0.43) |
| **12-month Timepoint** | | | | | | |
| NVDQ | -15.80 (2.62) | -24.23 (2.33) | -27.29 (2.48) | -8.43 (1.76) | -11.49 (1.77) | -3.06 (1.49) |
| VDQ | -16.00 (3.68) | -30.20 (3.37) | -34.25 (3.32) | -14.20 (1.90) | -18.25 (1.98) | -4.06 (1.59) |
| RL | -1.76 (0.69) | -4.86 (0.63) | -5.73 (0.66) | -3.11 (0.38) | -3.97 (0.43) | -0.86 (0.33) |
| EL | -2.58 (0.66) | -4.60 (0.63) | -5.47 (0.66) | -2.01 (0.36) | -2.89 (0.41) | -0.88 (0.32) |
| FM | -2.52 (0.39) | -3.59 (0.35) | -4.34 (0.37) | -1.07 (0.24) | -1.82 (0.25) | -0.75 (0.22) |
| GM | -2.61 (0.50) | -3.81 (0.44) | -4.09 (0.47) | -1.19 (0.26) | -1.47 (0.29) | -0.28 (0.24) |
| VR | -2.39 (0.58) | -4.18 (0.52) | -4.63 (0.55) | -1.80 (0.31) | -2.25 (0.33) | -0.45 (0.28) |
| **24-month Timepoint** | | | | | | |
| NVDQ | -16.82 (3.64) | -30.62 (3.38) | -35.63 (3.51) | -13.80 (2.32) | -18.81 (2.53) | -5.01 (1.99) |
| VDQ | -22.54 (6.57) | -48.00 (5.94) | -54.61 (5.67) | -25.46 (2.59) | -32.07 (2.91) | -6.61 (2.30) |
| RL | -2.95 (1.19) | -9.71 (1.05) | -11.41 (1.09) | -6.76 (0.58) | -8.47 (0.64) | -1.70 (0.52) |
| EL | -4.46 (1.26) | -8.60 (1.15) | -10.27 (1.14) | -4.15 (0.57) | -5.81 (0.64) | -1.66 (0.49) |
| FM | -3.77 (0.58) | -5.80 (0.53) | -7.38 (0.54) | -2.03 (0.35) | -3.60 (0.38) | -1.57 (0.31) |
| GM | -3.55 (0.86) | -5.92 (0.76) | -6.50 (0.78) | -2.38 (0.41) | -2.96 (0.46) | -0.58 (0.35) |
| VR | -4.27 (0.95) | -8.01 (0.85) | -8.84 (0.87) | -3.74 (0.49) | -4.57 (0.53) | -0.83 (0.42) |

Table S6. Estimated Marginal Means Contrasts for MSEL Age Equivalent Scores, Males Only

Table S7. Maternal Education of FXS and FH-ASD Groups

| Maternal Education, n (%) | FXS | FH-ASD | Test Statistic | P-value |
| --- | --- | --- | --- | --- |
| Less than college degree | 11 (36%) | 32 (42%) | X^2^= 3.7602 | .1526 |
| College degree | 14 (45%) | 27 (35%) |  |  |
| Graduate degree | 2 (6%) | 17 (22%) |  |  |
| Not available | 4 (13%) | 1 (1%) |  |  |

Table S8. FH-ASD Cognitive Level Group MSEL Raw Score Regression Results

|  | Estimate | SE | t-statistic | *df* | *p*-value |
| --- | --- | --- | --- | --- | --- |
| RL | | | | | |
| FXS | -0.17 | 0.57 | -0.30 | 1204.87 | 0.761 |
| FH-ASD-low | 1.29 | 0.65 | 1.98 | 679.65 | 0.048 |
| FH-ASD-avg/high | -0.16 | 0.48 | -0.33 | 173.27 | 0.745 |
| FH-nonASD | -0.22 | 0.29 | -0.77 | 1554.10 | 0.442 |
| Visit x FXS | -5.48 | 0.51 | -10.65 | 41.94 | < 0.0001 |
| Visit x FH-ASD-low | -7.33 | 0.50 | -14.72 | 985.49 | < 0.0001 |
| Visit x FH-ASD-avg/high | -2.22 | 0.38 | -5.85 | 121.37 | < 0.0001 |
| Visit x FH-nonASD | -0.64 | 0.22 | -2.93 | 1586.70 | 0.003 |
| EL | | | | | |
| FXS | -0.61 | 0.60 | -1.01 | 533.52 | 0.314 |
| FH-ASD-low | 0.52 | 0.68 | 0.75 | 359.26 | 0.451 |
| FH-ASD-avg/high | -0.24 | 0.47 | -0.51 | 1357.00 | 0.609 |
| FH-nonASD | -0.23 | 0.29 | -0.79 | 1578.63 | 0.432 |
| Visit x FXS | -4.72 | 0.55 | -8.56 | 27.20 | < 0.0001 |
| Visit x FH-ASD-low | -5.04 | 0.50 | -10.01 | 996.11 | < 0.0001 |
| Visit x FH-ASD-avg/high | -1.52 | 0.36 | -4.24 | 831.50 | < 0.0001 |
| Visit x FH-nonASD | -0.57 | 0.22 | -2.58 | 1495.76 | 0.010 |
| FM | | | | | |
| FXS | -2.00 | 0.44 | -4.53 | 71.24 | < 0.0001 |
| FH-ASD-low | -0.26 | 0.46 | -0.57 | 228.03 | 0.572 |
| FH-ASD-avg/high | -0.38 | 0.31 | -1.24 | 1403.91 | 0.217 |
| FH-nonASD | -0.27 | 0.20 | -1.33 | 617.46 | 0.184 |
| Visit x FXS | -2.33 | 0.36 | -6.52 | 20.41 | < 0.0001 |
| Visit x FH-ASD-low | -1.92 | 0.32 | -6.03 | 373.51 | < 0.0001 |
| Visit x FH-ASD-avg/high | -1.03 | 0.22 | -4.61 | 605.46 | < 0.0001 |
| Visit x FH-nonASD | -0.46 | 0.14 | -3.31 | 1428.55 | 0.001 |
| GM | | | | | |
| FXS | -2.13 | 0.51 | -4.16 | 98.27 | < 0.0001 |
| FH-ASD-low | -0.94 | 0.57 | -1.64 | 112.28 | 0.103 |
| FH-ASD-avg/high | -0.43 | 0.38 | -1.16 | 852.37 | 0.248 |
| FH-nonASD | -0.38 | 0.24 | -1.60 | 666.23 | 0.109 |
| Visit x FXS | -1.87 | 0.34 | -5.45 | 239.35 | < 0.0001 |
| Visit x FH-ASD-low | -1.04 | 0.38 | -2.73 | 467.57 | 0.006 |
| Visit x FH-ASD-avg/high | -0.91 | 0.27 | -3.38 | 376.50 | 0.001 |
| Visit x FH-nonASD | -0.17 | 0.17 | -1.03 | 631.56 | 0.303 |
| VR | | | | | |
| FXS | -1.51 | 0.54 | -2.81 | 582.62 | 0.005 |
| FH-ASD-low | -0.10 | 0.61 | -0.16 | 358.69 | 0.871 |
| FH-ASD-avg/high | -0.52 | 0.42 | -1.24 | 898.52 | 0.217 |
| FH-nonASD | -0.39 | 0.27 | -1.43 | 490.20 | 0.155 |
| Visit x FXS | -3.57 | 0.45 | -7.99 | 43.63 | < 0.0001 |
| Visit x FH-ASD-low | -3.60 | 0.44 | -8.21 | 619.88 | < 0.0001 |
| Visit x FH-ASD-avg/high | -1.38 | 0.30 | -4.57 | 1480.81 | < 0.0001 |
| Visit x FH-nonASD | -0.39 | 0.19 | -2.02 | 1234.01 | 0.043 |

Table S9. FH-ASD Cognitive Level Group MSEL Raw Score Estimated Marginal Means Contrasts

| Group Contrast Estimates (Standard Error) | | | |
| --- | --- | --- | --- |
|  | **FH-ASD-Low –**  **FH-ASD-Avg/High** | **FH-ASD-Low – FXS** | **FH-ASD-Avg/High – FXS** |
| **6-month Timepoint** | | | |
| RL | 1.44 (0.74) | 1.46 (0.79) | 0.02 (0.65) |
| EL | 0.75 (0.74) | 1.12 (0.83) | 0.37 (0.68) |
| FM | 0.12 (0.51) | 1.74 (0.61) | 1.61 (0.49) |
| GM | -0.50 (0.63) | 1.19 (0.68) | 1.70 (0.57) |
| VR | 0.42 (0.67) | 1.41 (0.74) | 0.99 (0.61) |
| **12-month Timepoint** | | | |
| RL | -3.66 (0.46) | -0.38 (0.54) | 3.28 (0.48) |
| EL | -2.76 (0.49) | 0.80 (0.60) | 3.56 (0.48) |
| FM | -0.78 (0.36) | 2.14 (0.40) | 2.92 (0.32) |
| GM | -0.63 (0.44) | 2.02 (0.49) | 2.65 (0.39) |
| VR | -1.79 (0.45) | 1.38 (0.55) | 3.17 (0.45) |
| **24-month Timepoint** | | | |
| RL | -8.77 (0.73) | -2.23 (0.91) | 6.54 (0.78) |
| EL | -6.28 (0.73) | 0.48 (0.98) | 6.76 (0.82) |
| FM | -1.67 (0.49) | 2.55 (0.60) | 4.22 (0.48) |
| GM | -0.75 (0.59) | 2.85 (0.66) | 3.60 (0.53) |
| VR | -4.01 (0.65) | 1.35 (0.84) | 5.36 (0.72) |

Table S10. FH-ASD Cognitive Level Group MSEL Composite and Age Equivalent Score Regression Results

|  | Estimate | SE | t-statistic | *df* | *p*-value |
| --- | --- | --- | --- | --- | --- |
| NVDQ | | | | | |
| FXS | -24.18 | 3.28 | -7.38 | 76.12 | < 0.0001 |
| FH-ASD-low | -6.23 | 3.50 | -1.78 | 177.31 | 0.077 |
| FH-ASD-avg/high | -7.19 | 2.41 | -2.98 | 379.49 | 0.003 |
| FH-nonASD | -4.10 | 1.50 | -2.74 | 810.92 | 0.006 |
| Visit x FXS | -6.56 | 2.34 | -2.80 | 53.57 | 0.007 |
| Visit x FH-ASD-low | -10.77 | 2.43 | -4.43 | 235.37 | < 0.0001 |
| Visit x FH-ASD-avg/high | -4.18 | 1.67 | -2.50 | 530.58 | 0.013 |
| Visit x FH-nonASD | -0.91 | 1.05 | -0.86 | 664.93 | 0.390 |
| VDQ | | | | | |
| FXS | -16.83 | 3.11 | -5.41 | 1475.07 | < 0.0001 |
| FH-ASD-low | -0.62 | 3.83 | -0.16 | 111.20 | 0.872 |
| FH-ASD-avg/high | -6.38 | 2.82 | -2.26 | 65.57 | 0.027 |
| FH-nonASD | -3.91 | 1.58 | -2.48 | 1313.52 | 0.013 |
| Visit x FXS | -19.30 | 2.49 | -7.76 | 45.49 | < 0.0001 |
| Visit x FH-ASD-low | -28.97 | 2.50 | -11.58 | 336.51 | < 0.0001 |
| Visit x FH-ASD-avg/high | -7.87 | 1.85 | -4.25 | 119.06 | < 0.0001 |
| Visit x FH-nonASD | -2.06 | 1.08 | -1.90 | 1154.88 | 0.058 |
| RL | | | | | |
| FXS | -0.14 | 0.70 | -0.20 | 1009.71 | 0.844 |
| FH-ASD-low | 1.61 | 0.78 | 2.08 | 1472.34 | 0.038 |
| FH-ASD-avg/high | 0.16 | 0.56 | 0.28 | 924.77 | 0.777 |
| FH-nonASD | -0.16 | 0.35 | -0.44 | 1283.25 | 0.657 |
| Visit x FXS | -6.38 | 0.57 | -11.14 | 139.18 | < 0.0001 |
| Visit x FH-ASD-low | -8.78 | 0.60 | -14.56 | 1275.53 | < 0.0001 |
| Visit x FH-ASD-avg/high | -3.03 | 0.44 | -6.85 | 369.53 | < 0.0001 |
| Visit x FH-nonASD | -0.97 | 0.27 | -3.60 | 1437.64 | < 0.0001 |
| EL | | | | | |
| FXS | -0.82 | 0.70 | -1.17 | 809.82 | 0.242 |
| FH-ASD-low | 0.66 | 0.78 | 0.85 | 1381.88 | 0.394 |
| FH-ASD-avg/high | -0.16 | 0.56 | -0.29 | 914.76 | 0.772 |
| FH-nonASD | -0.26 | 0.35 | -0.74 | 1332.59 | 0.462 |
| Visit x FXS | -5.08 | 0.60 | -8.47 | 52.90 | < 0.0001 |
| Visit x FH-ASD-low | -6.04 | 0.58 | -10.35 | 1568.95 | < 0.0001 |
| Visit x FH-ASD-avg/high | -1.88 | 0.43 | -4.40 | 490.23 | < 0.0001 |
| Visit x FH-nonASD | -0.74 | 0.27 | -2.78 | 923.53 | 0.006 |
| FM | | | | | |
| FXS | -1.80 | 0.48 | -3.77 | 261.03 | < 0.0001 |
| FH-ASD-low | 0.05 | 0.53 | 0.10 | 340.27 | 0.917 |
| FH-ASD-avg/high | -0.28 | 0.37 | -0.77 | 621.79 | 0.444 |
| FH-nonASD | -0.12 | 0.23 | -0.52 | 774.48 | 0.601 |
| Visit x FXS | -2.89 | 0.35 | -8.19 | 108.81 | < 0.0001 |
| Visit x FH-ASD-low | -2.58 | 0.38 | -6.79 | 374.91 | < 0.0001 |
| Visit x FH-ASD-avg/high | -1.40 | 0.26 | -5.31 | 707.31 | < 0.0001 |
| Visit x FH-nonASD | -0.70 | 0.17 | -4.18 | 777.63 | < 0.0001 |
| GM | | | | | |
| FXS | -1.77 | 0.55 | -3.20 | 302.03 | 0.002 |
| FH-ASD-low | -0.50 | 0.60 | -0.83 | 1151.43 | 0.404 |
| FH-ASD-avg/high | -0.13 | 0.43 | -0.30 | 574.43 | 0.766 |
| FH-nonASD | -0.24 | 0.27 | -0.88 | 955.78 | 0.379 |
| Visit x FXS | -2.46 | 0.40 | -6.13 | 403.02 | < 0.0001 |
| Visit x FH-ASD-low | -1.86 | 0.44 | -4.24 | 1407.37 | < 0.0001 |
| Visit x FH-ASD-avg/high | -1.40 | 0.32 | -4.43 | 934.41 | < 0.0001 |
| Visit x FH-nonASD | -0.39 | 0.20 | -1.91 | 416.60 | 0.057 |
| VR | | | | | |
| FXS | -0.64 | 0.65 | -0.99 | 242.97 | 0.324 |
| FH-ASD-low | 0.73 | 0.70 | 1.03 | 1061.78 | 0.301 |
| FH-ASD-avg/high | 0.00 | 0.50 | 0.00 | 1225.08 | 0.996 |
| FH-nonASD | -0.11 | 0.31 | -0.36 | 1595.98 | 0.718 |
| Visit x FXS | -4.62 | 0.54 | -8.63 | 71.08 | < 0.0001 |
| Visit x FH-ASD-low | -4.69 | 0.54 | -8.67 | 1159.38 | < 0.0001 |
| Visit x FH-ASD-avg/high | -2.07 | 0.38 | -5.40 | 1098.97 | < 0.0001 |
| Visit x FH-nonASD | -0.71 | 0.24 | -2.95 | 1554.91 | < 0.0001 |

Table S11. FH-ASD Cognitive Level Group MSEL Composite and Age Equivalent Score Estimated Marginal Means Contrasts

| Group Contrast Estimates (Standard Error) | | | |
| --- | --- | --- | --- |
|  | **FH-ASD-Low –**  **FH-ASD-Avg/High** | **FH-ASD-Low – FXS** | **FH-ASD-Avg/High – FXS** |
| **6-month Timepoint** | | | |
| NVDQ | 0.96 (3.93) | 17.95 (4.75) | 16.99 (3.52) |
| VDQ | 5.77 (4.39) | 16.21 (4.49) | 10.44 (3.75) |
| RL | 1.46 (0.86) | 1.75 (0.96) | 0.3 (0.79) |
| EL | 0.83 (0.87) | 1.49 (0.97) | 0.66 (0.79) |
| FM | 0.34 (0.59) | 1.85 (0.66) | 1.51 (0.54) |
| GM | -0.37 (0.67) | 1.27 (0.74) | 1.64 (0.65) |
| VR | 0.73 (0.77) | 1.37 (0.89) | 0.64 (0.72) |
| **12-month Timepoint** | | | |
| NVDQ | -5.63 (2.63) | 13.75 (3.17) | 19.38 (2.57) |
| VDQ | -15.33 (2.99) | 6.54 (3.38) | 21.87 (3.16) |
| RL | -4.3 (0.55) | -0.65 (0.65) | 3.65 (0.53) |
| EL | -3.33 (0.57) | 0.52 (0.69) | 3.85 (0.57) |
| FM | -0.84 (0.39) | 2.16 (0.46) | 3.01 (0.39) |
| GM | -0.84 (0.45) | 1.86 (0.52) | 2.7 (0.46) |
| VR | -1.89 (0.49) | 1.3 (0.56) | 3.19 (0.47) |
| **24-month Timepoint** | | | |
| NVDQ | -12.22 (3.66) | 9.54 (4.32) | 21.76 (3.59) |
| VDQ | -36.42 (3.88) | -3.13 (4.96) | 33.29 (4.26) |
| RL | -10.05 (0.87) | -3.05 (1.06) | 7 (0.89) |
| EL | -7.49 (0.88) | -0.44 (1.11) | 7.05 (0.92) |
| FM | -2.02 (0.56) | 2.48 (0.66) | 4.5 (0.55) |
| GM | -1.31 (0.65) | 2.46 (0.77) | 3.77 (0.65) |
| VR | -4.52 (0.78) | 1.23 (0.94) | 5.74 (0.78) |

Table S12. 24-month ASD Data Availability for FXS Infants

| Participant Number | Sex | 24-month Visit Attended | ADOS Administered | ADOS Classification | DSM Administered | DSM Classification | MSEL ELC Score (at 24-months unless otherwise specified) |
| --- | --- | --- | --- | --- | --- | --- | --- |
| 1 | Male | YES | NO | NA | NO | NA | 52 |
| 2 | Female | YES | ados2_module1 | None | YES | NO (DSM_IV questions 4a/4b is No at V24) | 52 |
| 3 | Male | NO | NA | NA | NA | NA | 66 (12-month) |
| 4 | Female | YES | ados2_module1 | None | YES | NO (DSM_IV questions 4a/4b is No at V24) | 98 |
| 5 | Male | NO | NA | NA | NA | NA | 71 (6-month) |
| 6 | Female | YES | ados2_module1 | Autism Spectrum | NO | NA | 74 |
| 7 | Male | YES | ados2_module1 | None | YES | NO (DSM_IV questions 4a/4b is No at V24) | 61 |
| 8 | Female | YES | ados2_module1 | Autism | NO | NA | 56 |
| 9 | Male | YES | ados2_module1 | Autism Spectrum | NO | NA | 67 |
| 10 | Male | YES | ados_module1 | None | YES | YES (DSM_IV questions 4a/4b is Yes) | 54 |
| 11 | Male | YES | NO | NA | NO | NA | 49 |
| 12 | Male | YES | ados2_module2 | INVALID | NO | NA | 82 |
| 13 | Male | YES | ados2_module1 | Autism Spectrum | YES | YES (DSM_IV questions 4a/4b is Yes) | 60 |
| 14 | Male | YES | ados_module1 | Autism Spectrum | YES | YES (DSM_IV questions 4a/4b is Yes) | 51 |
| 15 | Male | NO | NA | NA | NA | NA | 80 (12-month) |
| 16 | Male | YES | ados2_module1 | None | NO | NA | 68 |
| 17 | Male | YES | ados2_module1 | Autism | YES | YES (DSM_IV questions 4a/4b is Yes) | 50 |
| 18 | Male | YES | NO | NA | NO | NA | 63 (12-month) |
| 19 | Male | YES | NO | NA | NO | NA | 49 |
| 20 | Male | YES | NO | NA | NO | NA | 50 |
| 21 | Male | YES | ados_module1 | None | YES | NO (DSM_IV questions 4a/4b is No at V24) | 73 |
| 22 | Male | NO | NA | NA | NA | NA | 76 (12-month) |
| 23 | Male | YES | ados2_module1 | None | YES | NO (DSM_IV questions 4a/4b is No at V24) | 54 |
| 24 | Male | NO | NA | NA | NA | NA | 64 (12-month) |
| 25 | Male | NO | NA | NA | NA | NA | 84 (12-month) |
| 26 | Male | NO | NA | NA | NA | NA | 92 (6-month) |
| 27 | Female | YES | ados_module1 | None | YES | NO (DSM_IV questions 4a/4b is No at V24) | 93 |
| 28 | Female | YES | ados2_module1 | None | NO | NA | 67 |
| 29 | Male | YES | ados2_module1 | None | NO | NA | 56 |
| 30 | Male | NO | NA | NA | NA | NA | 60 (12-month) |
| 31 | Male | YES | ados2_module1 | INVALID | NO | NA | 49 |

*Note*. The ADOS for participant 31 was considered invalid due to severe motor delays interfering with ability to carry out the assessment. Information on why the ADOS for participant 12 was considered invalid is not available.

**SUPPLEMENTAL RESULTS - FIGURES**

Figure S1. MSEL Receptive Language Age Equivalent Score Trajectory by Group and Timepoint


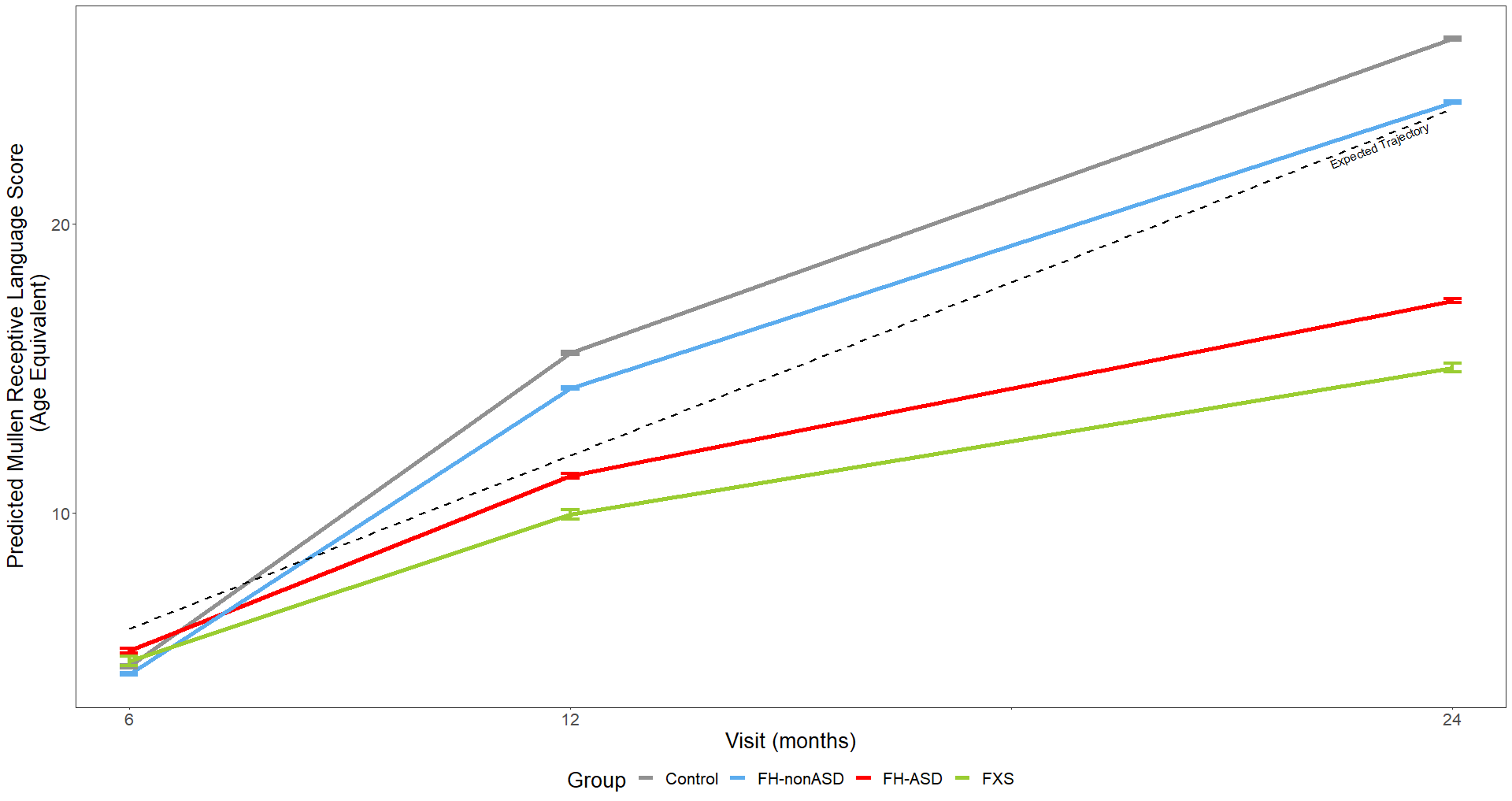


Model-generated differential trajectories of Receptive Language mean age equivalent score from the MSEL by group and timepoint (Visit). At 6 months, no group trajectories significantly differed from one another. By 12 months of age a pattern emerged between groups (Table S3) that persisted through 24 months, such that all groups except FXS and FH-ASD significantly differed from one another.

Figure S2. MSEL Expressive Language Age Equivalent Score Trajectory by Group and Timepoint


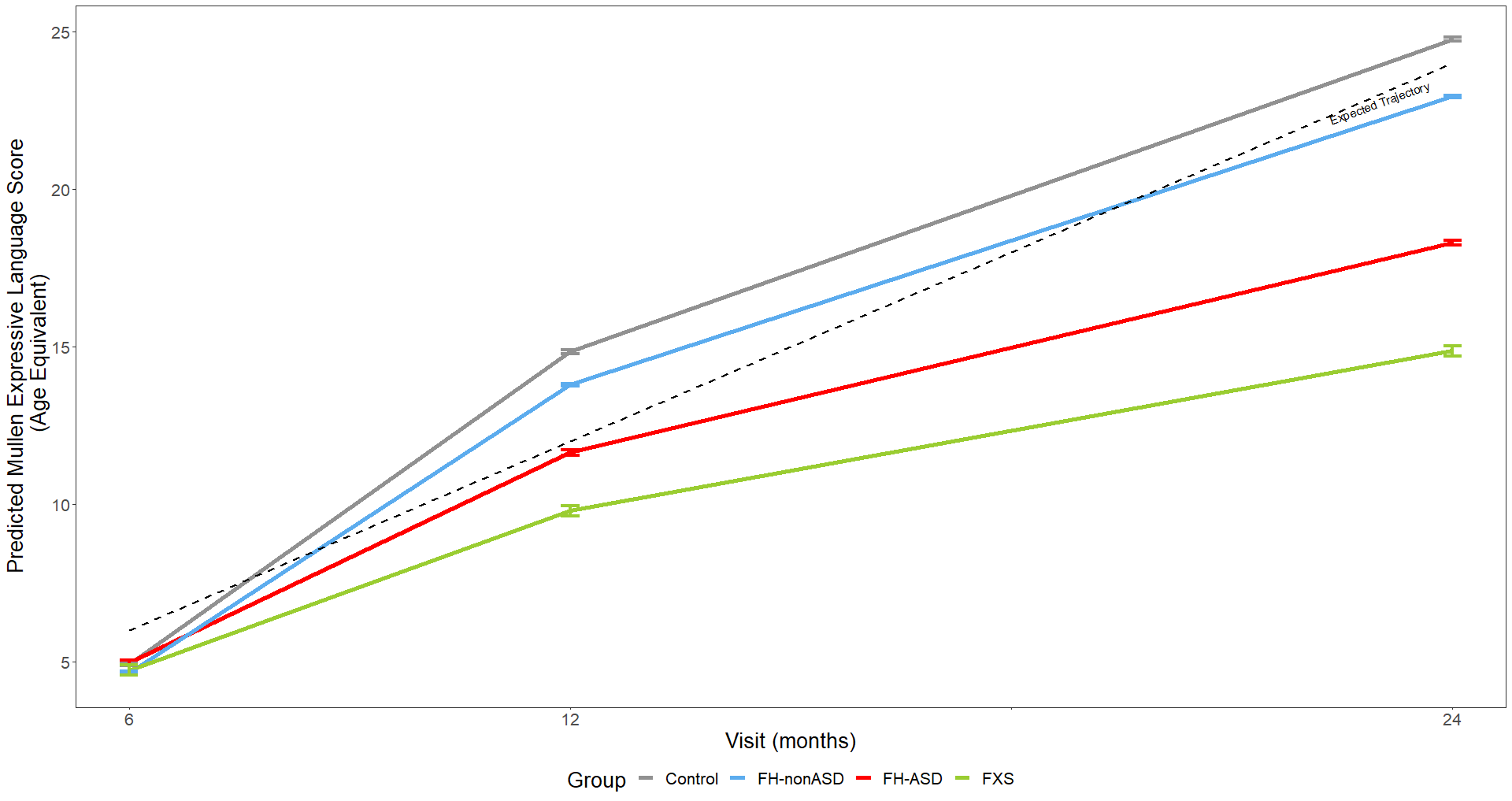


Model-generated differential trajectories of Expressive Language mean age equivalent score from the MSEL by group and timepoint (Visit). At 6 months, no group trajectories significantly differed from one another. By 12 months of age a pattern emerged between groups (Table S3) that persisted through 24 months, such that control > FH-nonASD > FH-ASD > FXS.

Figure S3. MSEL Fine Motor Age Equivalent Score Trajectory by Group and Timepoint


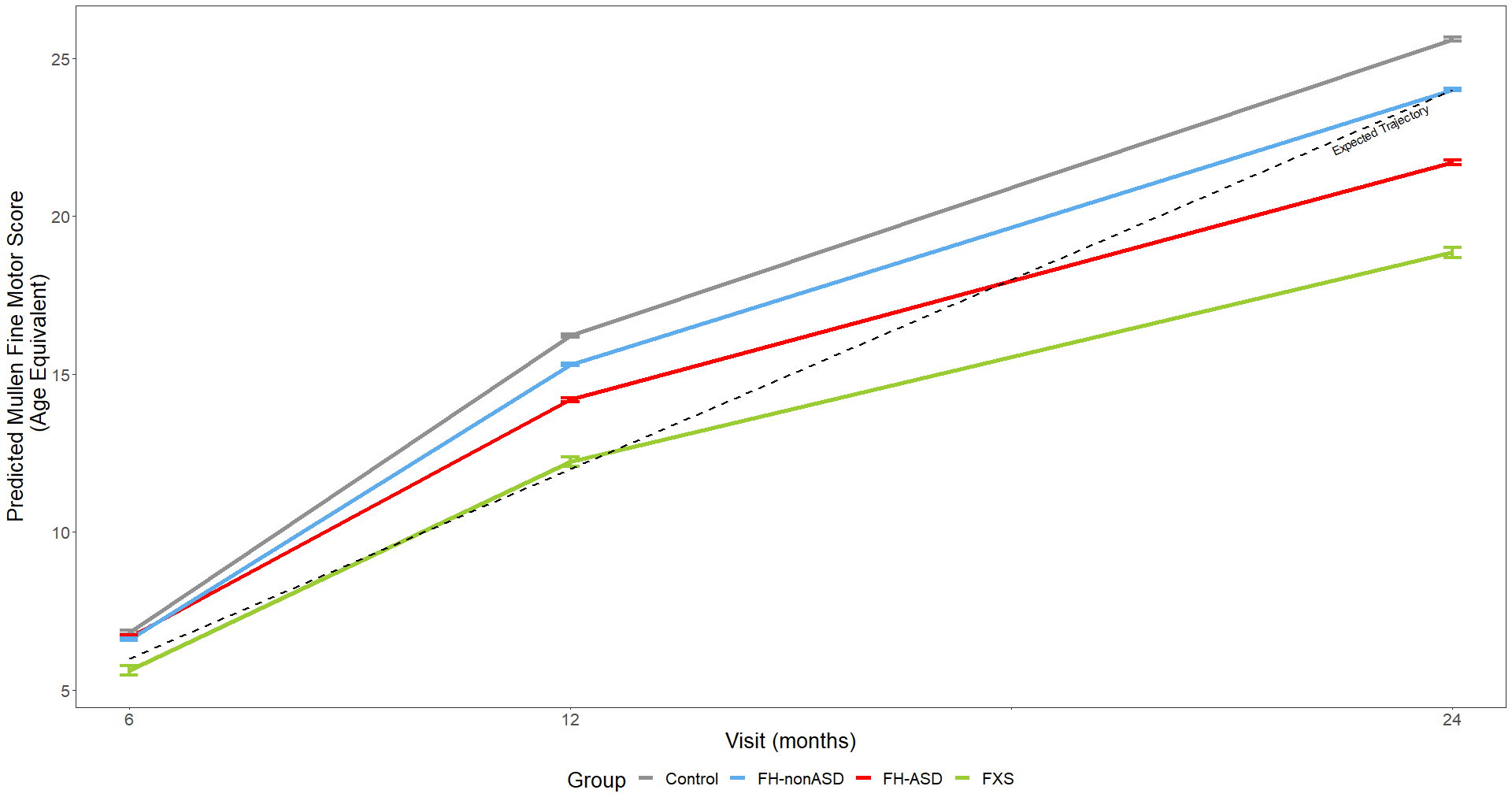


Model-generated differential trajectories of Fine Motor mean age equivalent score from the MSEL by group and timepoint (Visit). At 6 months, no group trajectories significantly differed from one another. By 12 months of age a pattern emerged between groups (Table S3) that persisted through 24 months, such that control > FH-nonASD > FH-ASD > FXS.

Figure S4. MSEL Gross Motor Age Equivalent Score Trajectory by Group and Timepoint


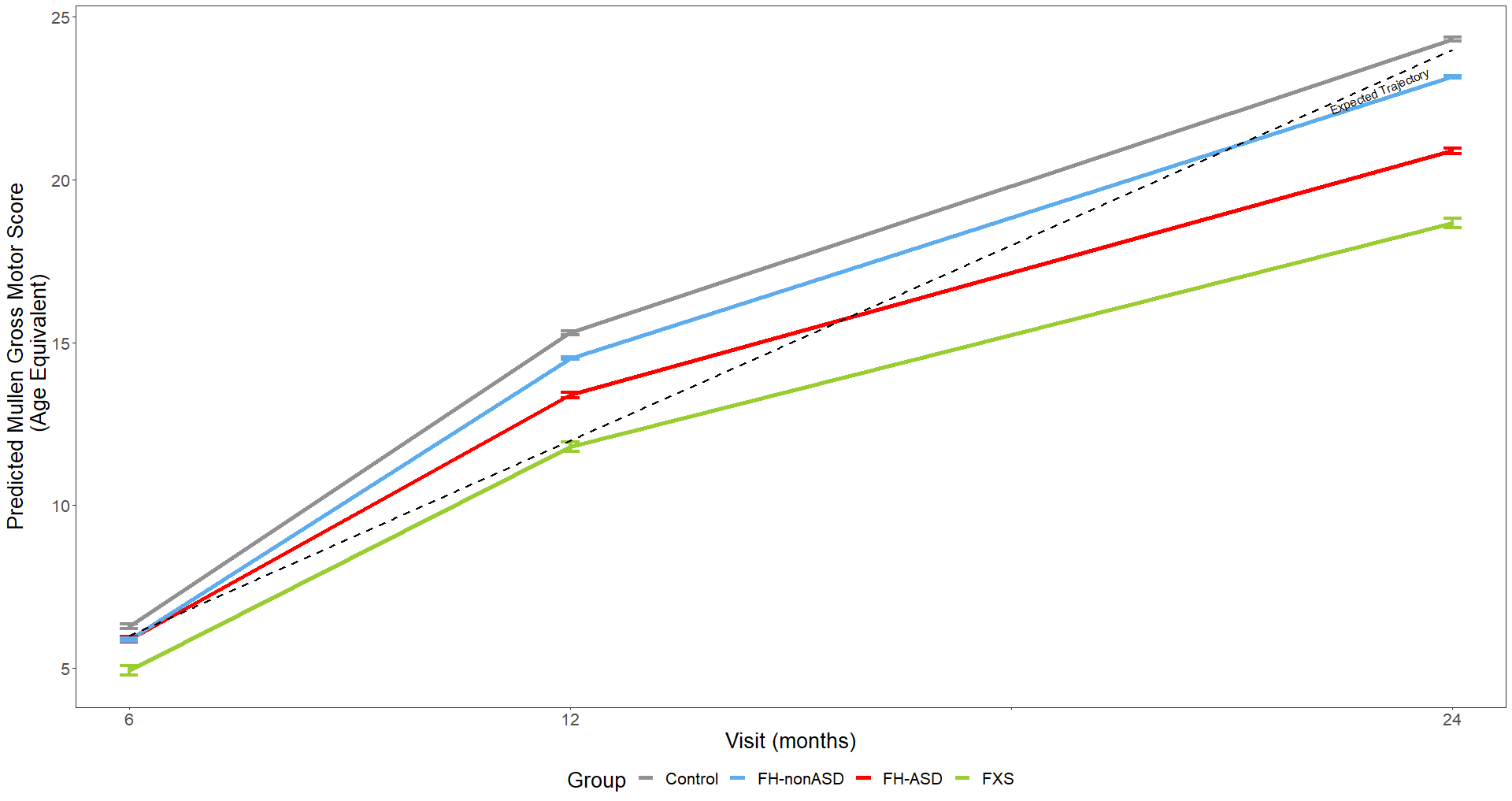


Model-generated differential trajectories of Gross Motor mean age equivalent score from the MSEL by group and timepoint (Visit). At 6 months, no group trajectories significantly differed from one another. At 12 months, all groups significantly differed from one another such that control > FH-nonASD > FH-ASD > FXS. At 24 months, a similar pattern persisted, except for FH-nonASD and controls not significantly differing (Table S3).

Figure S5. MSEL Visual Reception Age Equivalent Score Trajectory by Group and Timepoint


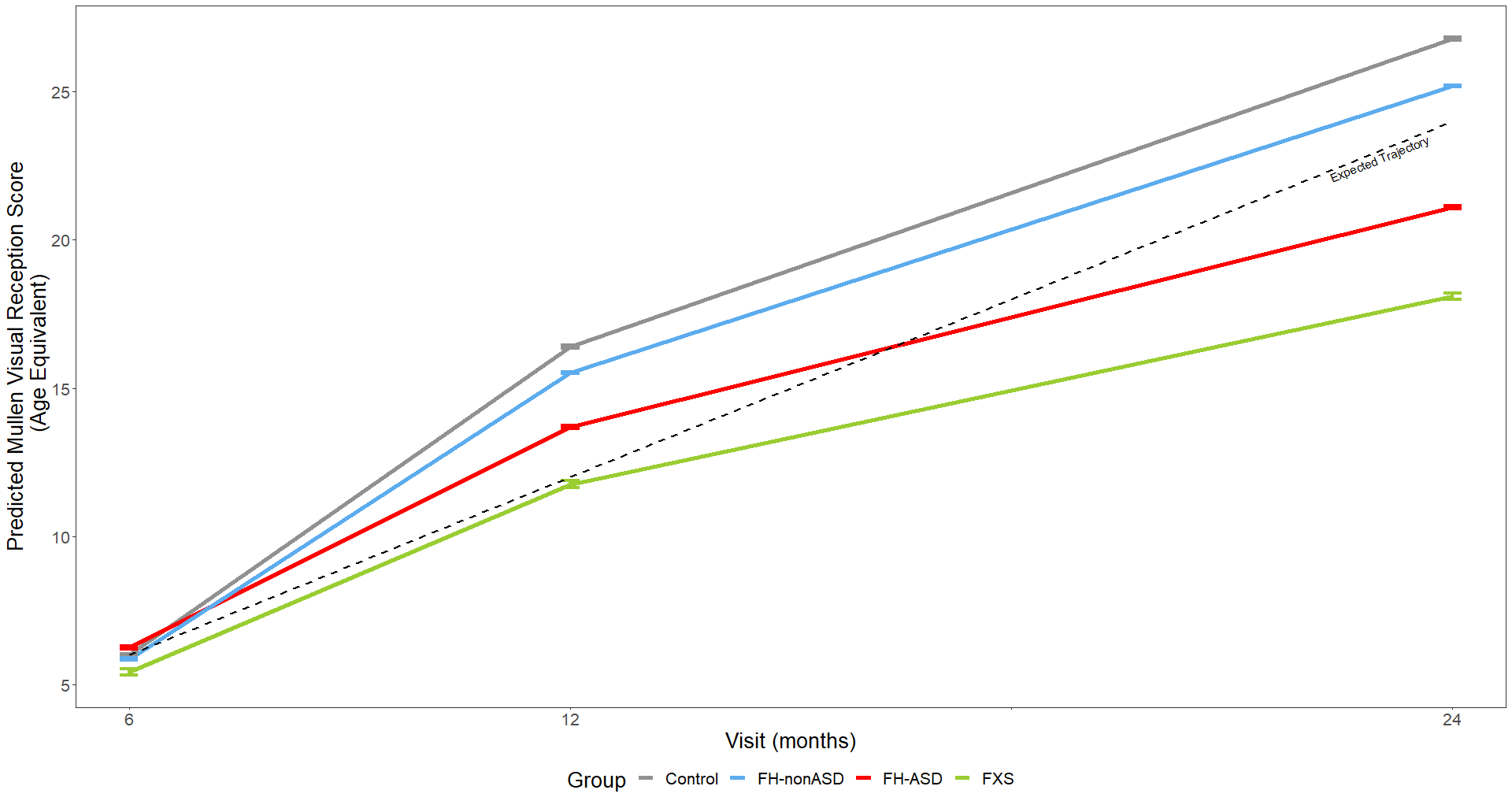


Model-generated differential trajectories of Visual Reception mean age equivalent score from the MSEL by group and timepoint (Visit). At 6 months, no group trajectories significantly differed from one another. By 12 months of age a pattern emerged between groups (Table S3) that persisted through 24 months, such that control > FH-nonASD > FH-ASD > FXS.

Figure S6. MSEL NVDQ Score Trajectory by Group and Timepoint, Males Only


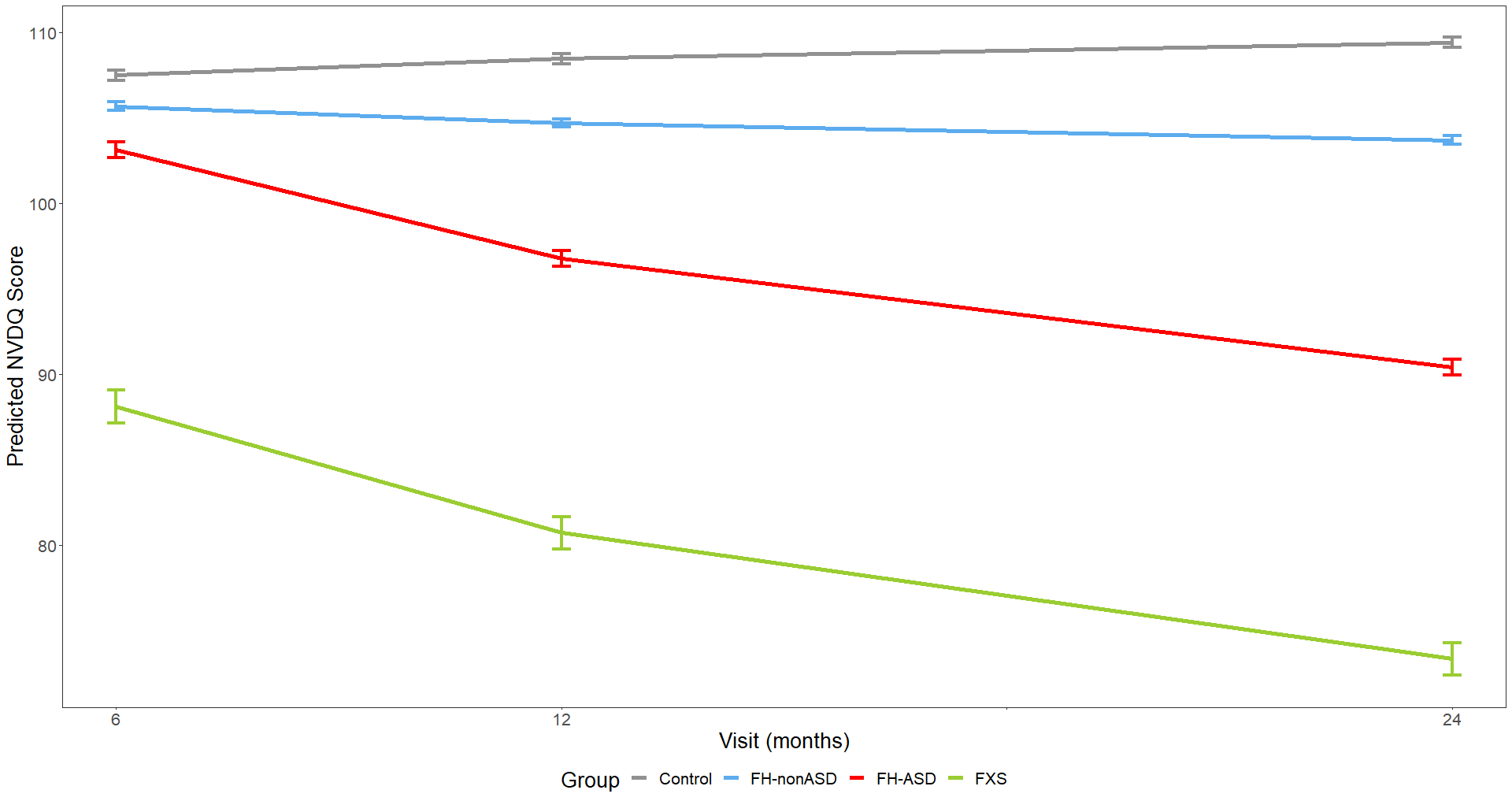


Model-generated differential trajectories of derived Nonverbal Developmental Quotient (NVDQ) from the MSEL by group and timepoint (Visit) for males only. The FXS group exhibited significantly delayed trajectories of NVDQ from 6-24 months compared to all other groups. By 12 months of age a pattern emerged between groups (Table S4) that persisted through 24 months, such that all groups significantly differed from one another except for FH-nonASD and control groups.

Figure S7. MSEL VDQ Score Trajectory by Group and Timepoint, Males Only


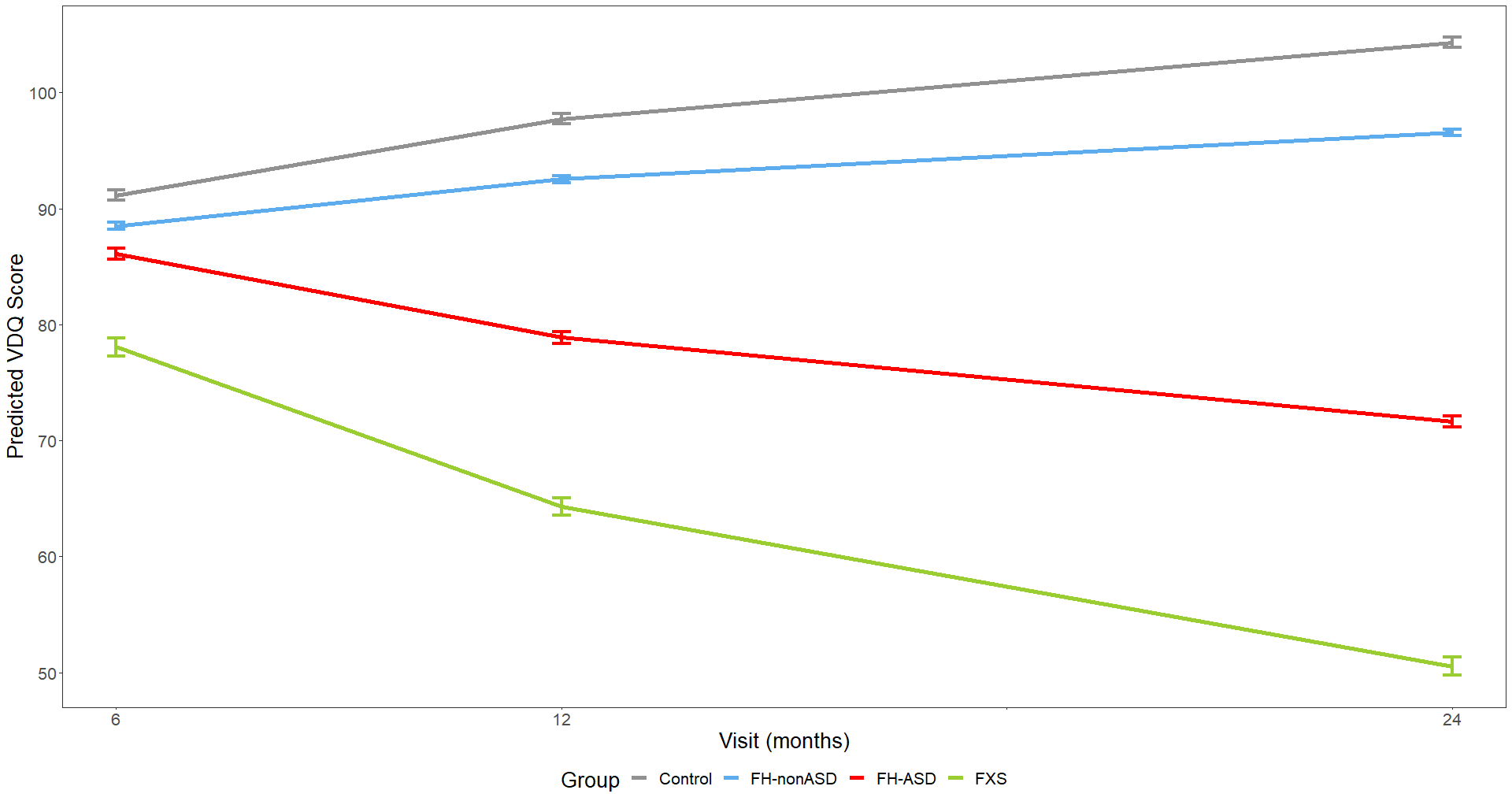


Model-generated differential trajectories of derived Verbal Developmental Quotient (VDQ) from the MSEL by group and timepoint (Visit) for males only. The FXS group exhibited significantly delayed trajectories of VDQ from 6-24 months compared to FH-nonASD and controls. By 12 months of age a pattern emerged between groups (Table S4) that persisted through 24 months, such that all groups significantly differed from one another except FH-nonASD and controls.

Figure S8. MSEL Receptive Language Age Equivalent Score Trajectory by Group and Timepoint, Males Only


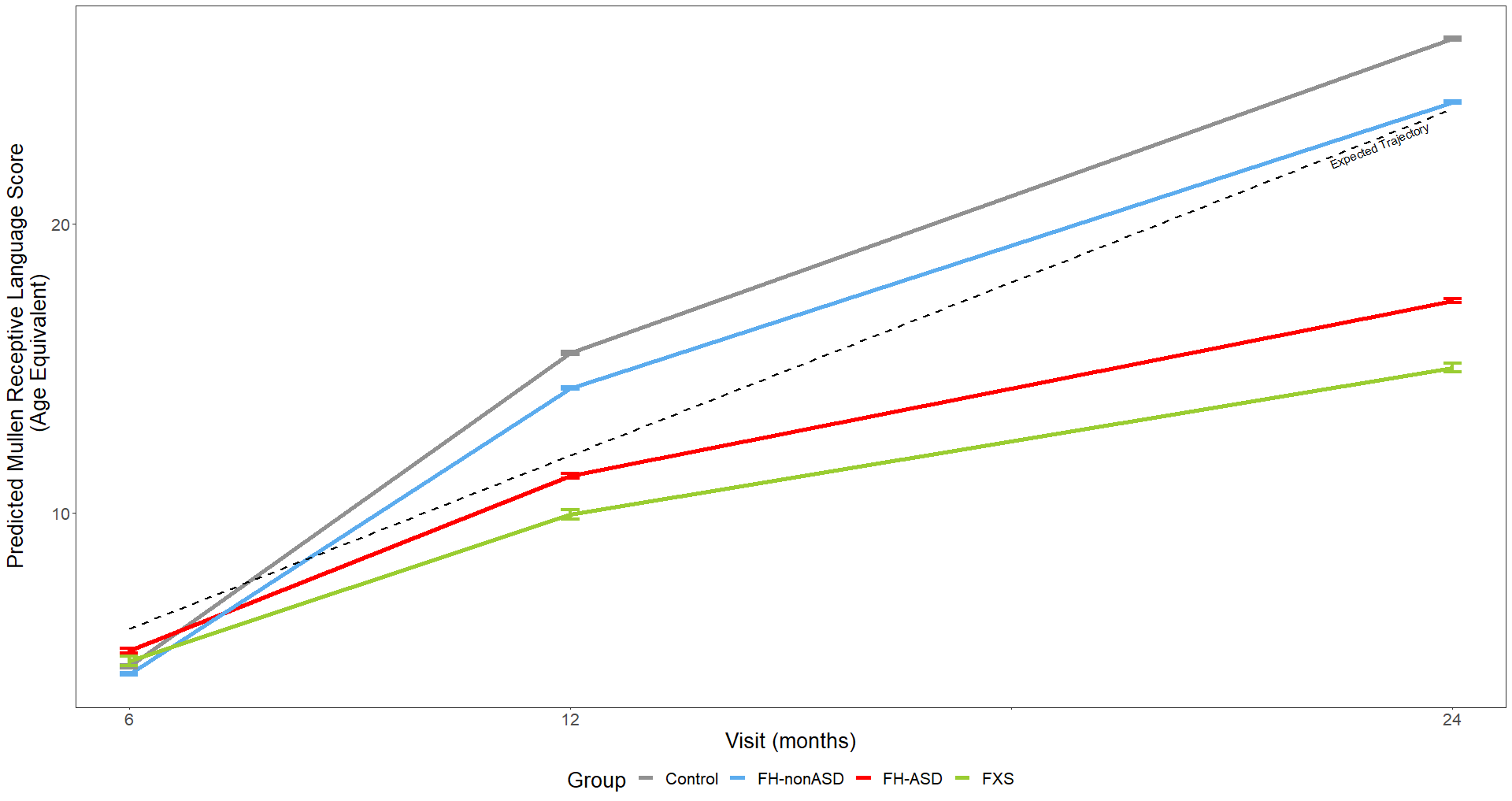


Model-generated differential trajectories of Receptive Language from the MSEL by group and timepoint (Visit) for males only. At 6 months, no group trajectories significantly differed from one another. At 12 months, all group trajectories except between FXS and FH-ASD, and between FH-nonASD and control significantly differed from one another. At 24 months, all groups except FXS and FH-ASD significantly differed from one another (Table S4).

Figure S9. MSEL Expressive Language Age Equivalent Score Trajectory by Group and Timepoint, Males Only


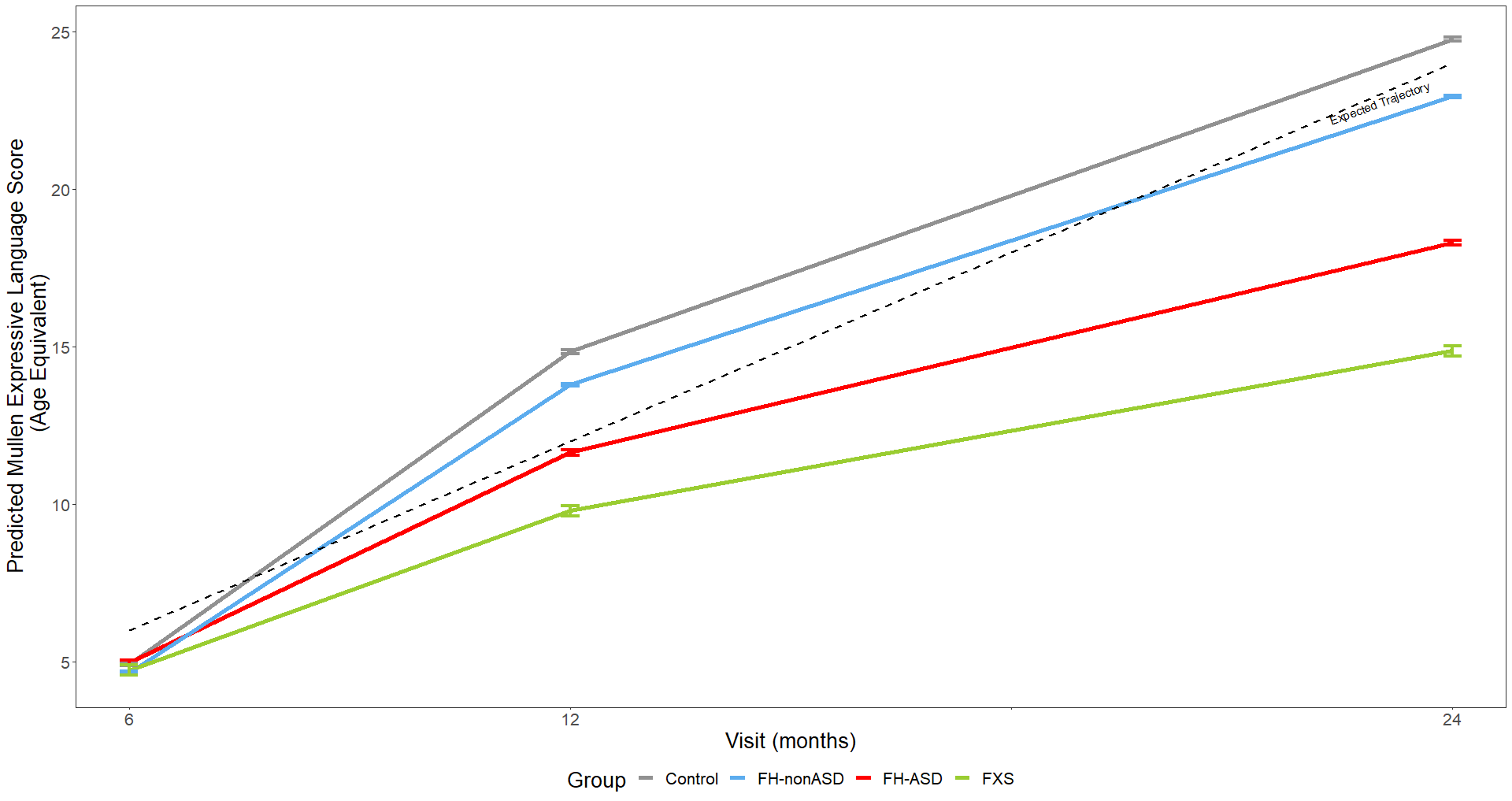


Model-generated differential trajectories of Expressive Language from the MSEL by group and timepoint (Visit) for males only. At 6 months, no group trajectories significantly differed from one another. At 12 months, all groups except FH-nonASD and control significantly differed from one another. At 24 months, all groups significantly differed from one another such that control > FH-nonASD > FH-ASD > FXS (Table S4).

Figure S10. MSEL Fine Motor Age Equivalent Score Trajectory by Group and Timepoint, Males Only


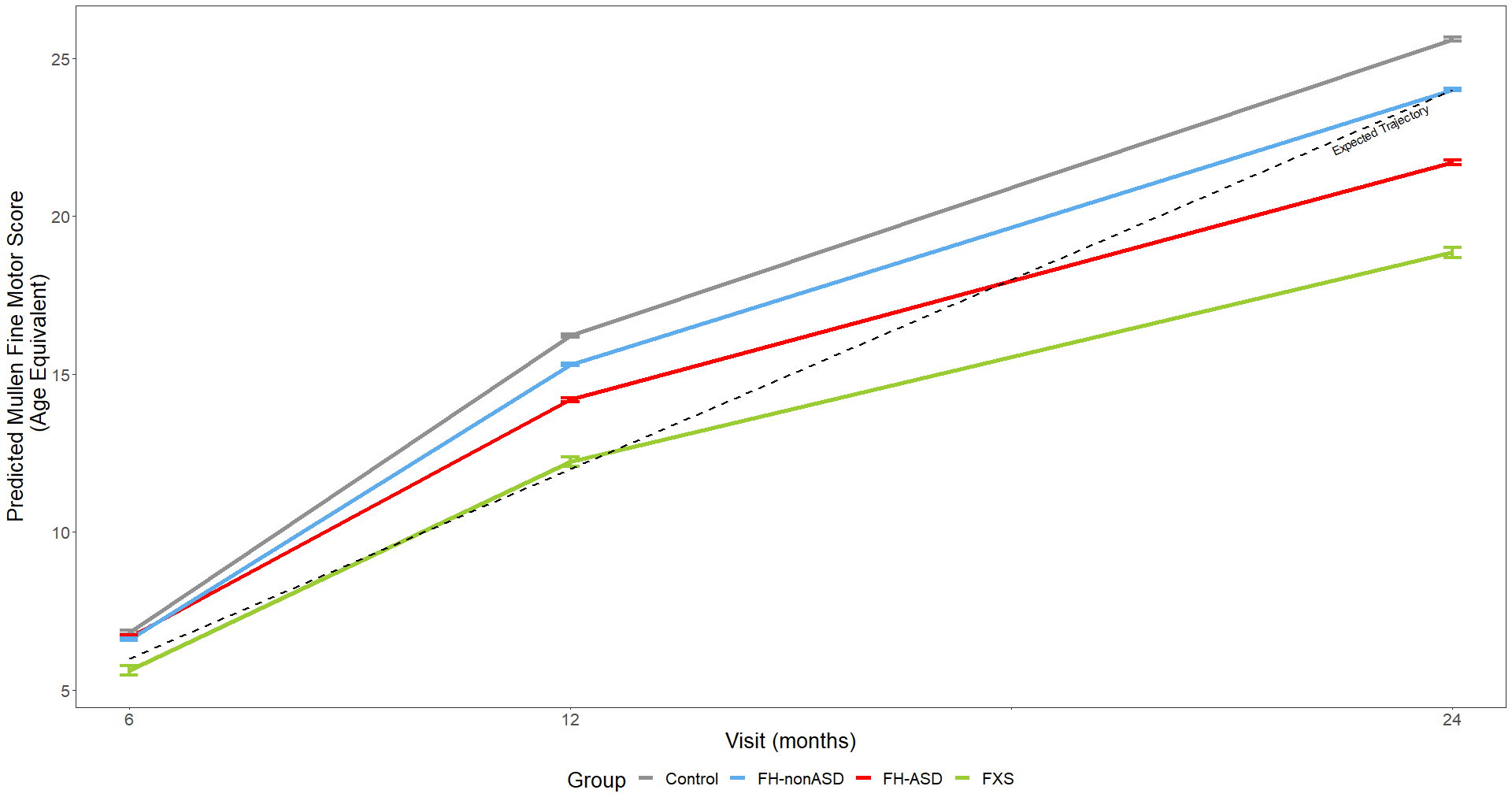


Model-generated differential trajectories of Fine Motor from the MSEL by group and timepoint (Visit) for males only. At 6 months, no group trajectories significantly differed from one another. By 12 months of age a pattern emerged between groups (Table S4) that persisted through 24 months, such that control > FH-nonASD > FH-ASD > FXS.

Figure S11. MSEL Gross Motor Age Equivalent Score Trajectory by Group and Timepoint, Males Only


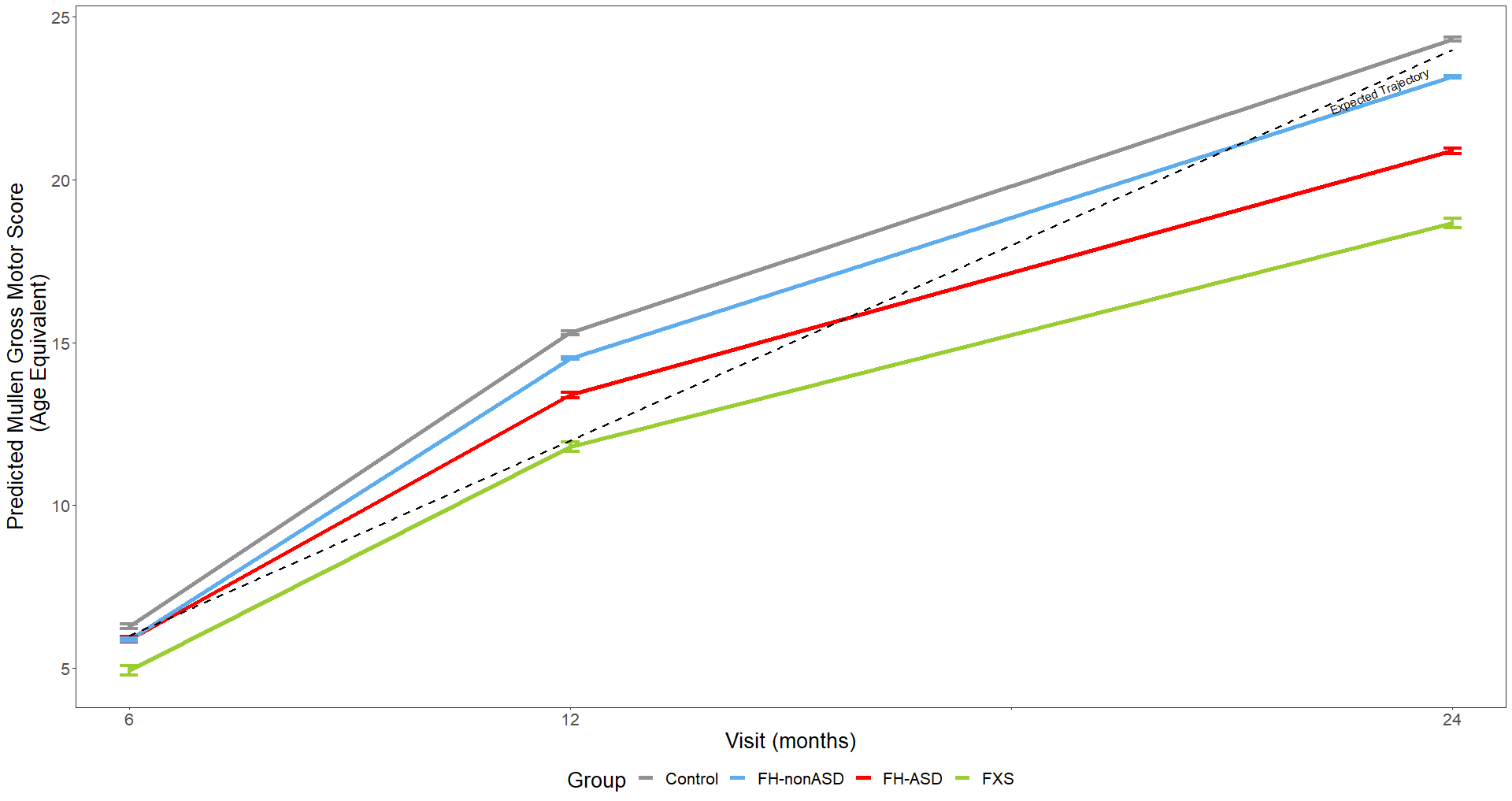


Model-generated differential trajectories of Gross Motor from the MSEL by group and timepoint (Visit) for males only. At 6 months, no group trajectories significantly differed from one another. At 12 and 24 months, all groups except FH-nonASD and control significantly differed from one another (Table S4).

Figure S12. MSEL Visual Reception Age Equivalent Score Trajectory by Group and Timepoint, Males Only


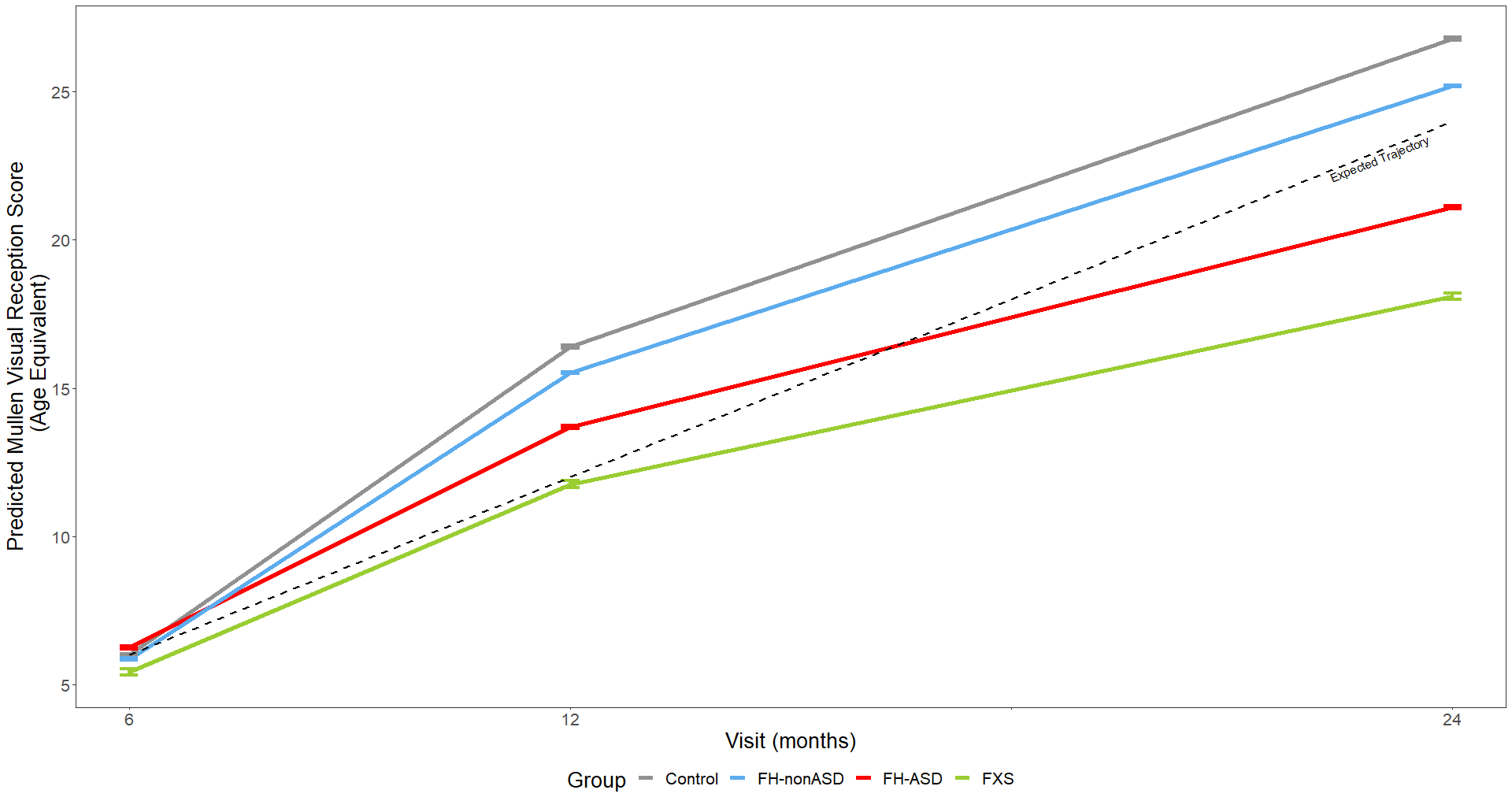


Model-generated differential trajectories of Visual Reception from the MSEL by group and timepoint (Visit) for males only. At 6 months, no group trajectories significantly differed from one another. At 12 and 24 months, all groups except FH-nonASD and control significantly differed from one another (Table S4).

Figure S13. MSEL Receptive Language Raw Score Trajectory by Group and Timepoint, Males Only


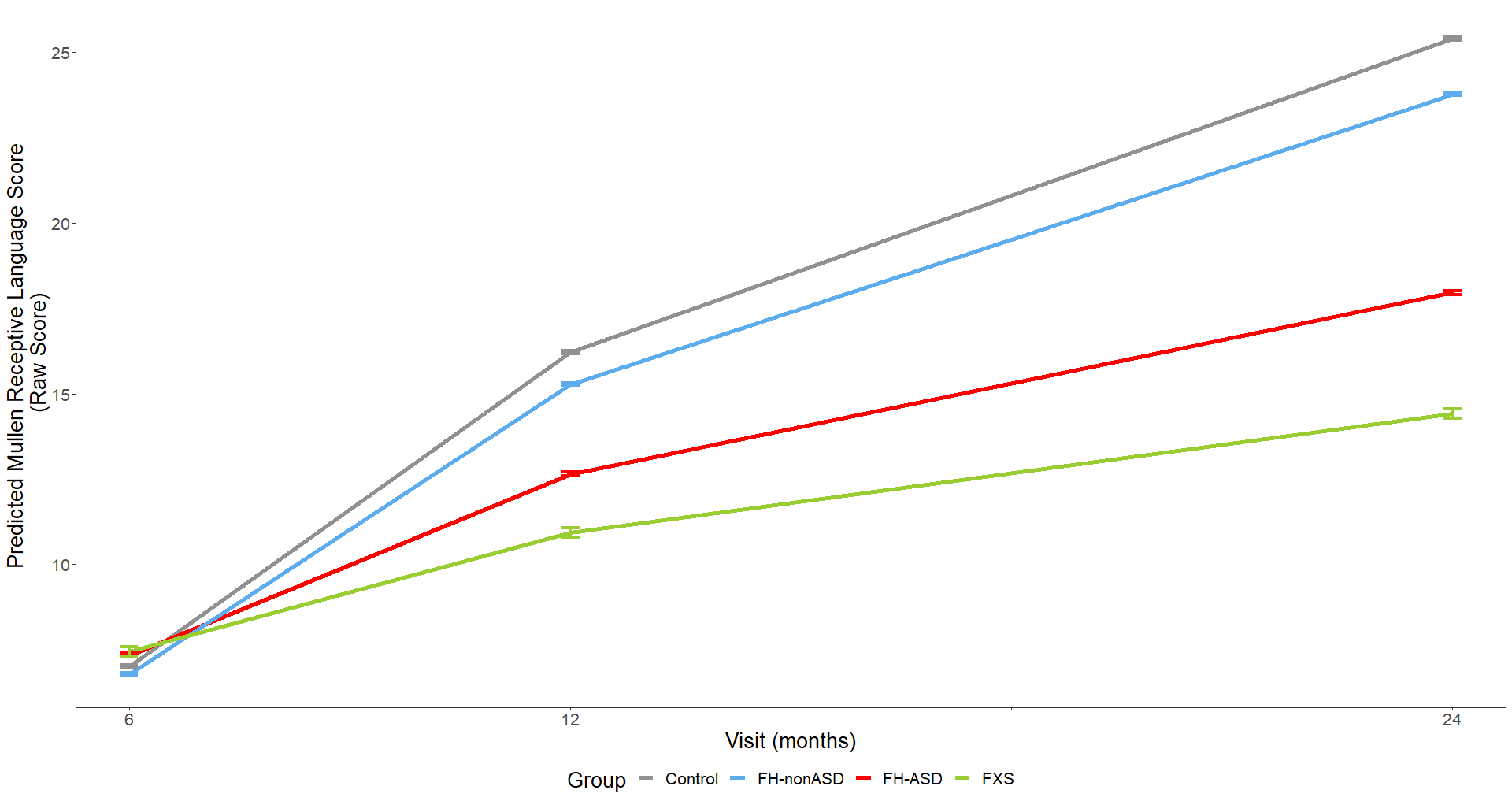


Model-generated differential trajectories of Receptive Language mean raw score from the MSEL by group and timepoint (Visit) for males only. At 6 months, no group trajectories significantly differed from one another. By 12 months of age a pattern emerged between groups (Table S5) that persisted through 24 months, such that all groups significantly differed from one another except for FH-nonASD and control groups.

Figure S14. MSEL Expressive Language Raw Score Trajectory by Group and Timepoint, Males Only


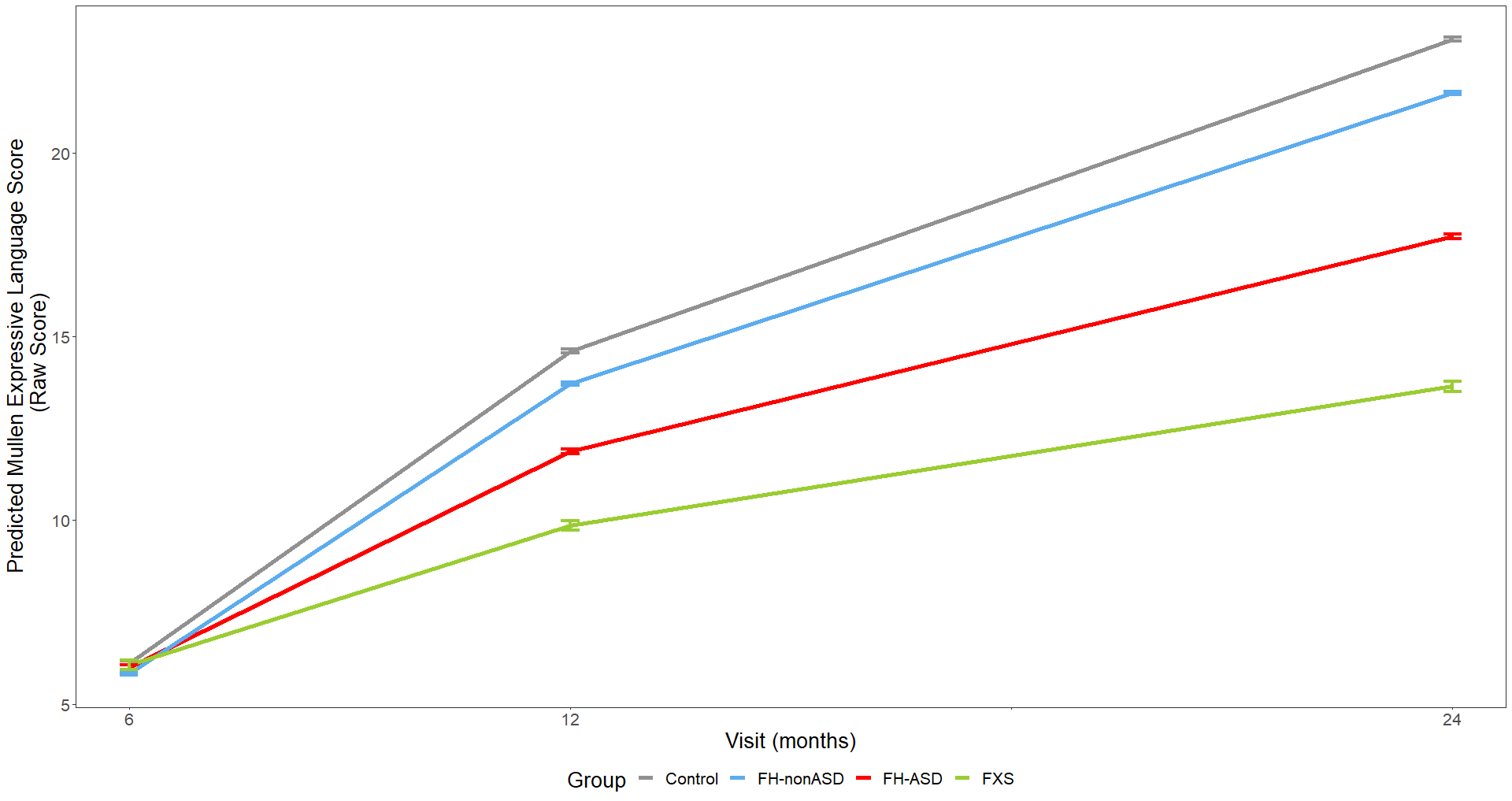


Model-generated differential trajectories of Expressive Language mean raw score from the MSEL by group and timepoint (Visit) for males only. At 6 months, no group trajectories significantly differed from one another. By 12 months of age a pattern emerged between groups (Table S5) that persisted through 24 months, such that all groups significantly differed from one another except for FH-nonASD and control groups.

Figure S15. MSEL Fine Motor Raw Score Trajectory by Group and Timepoint, Males Only


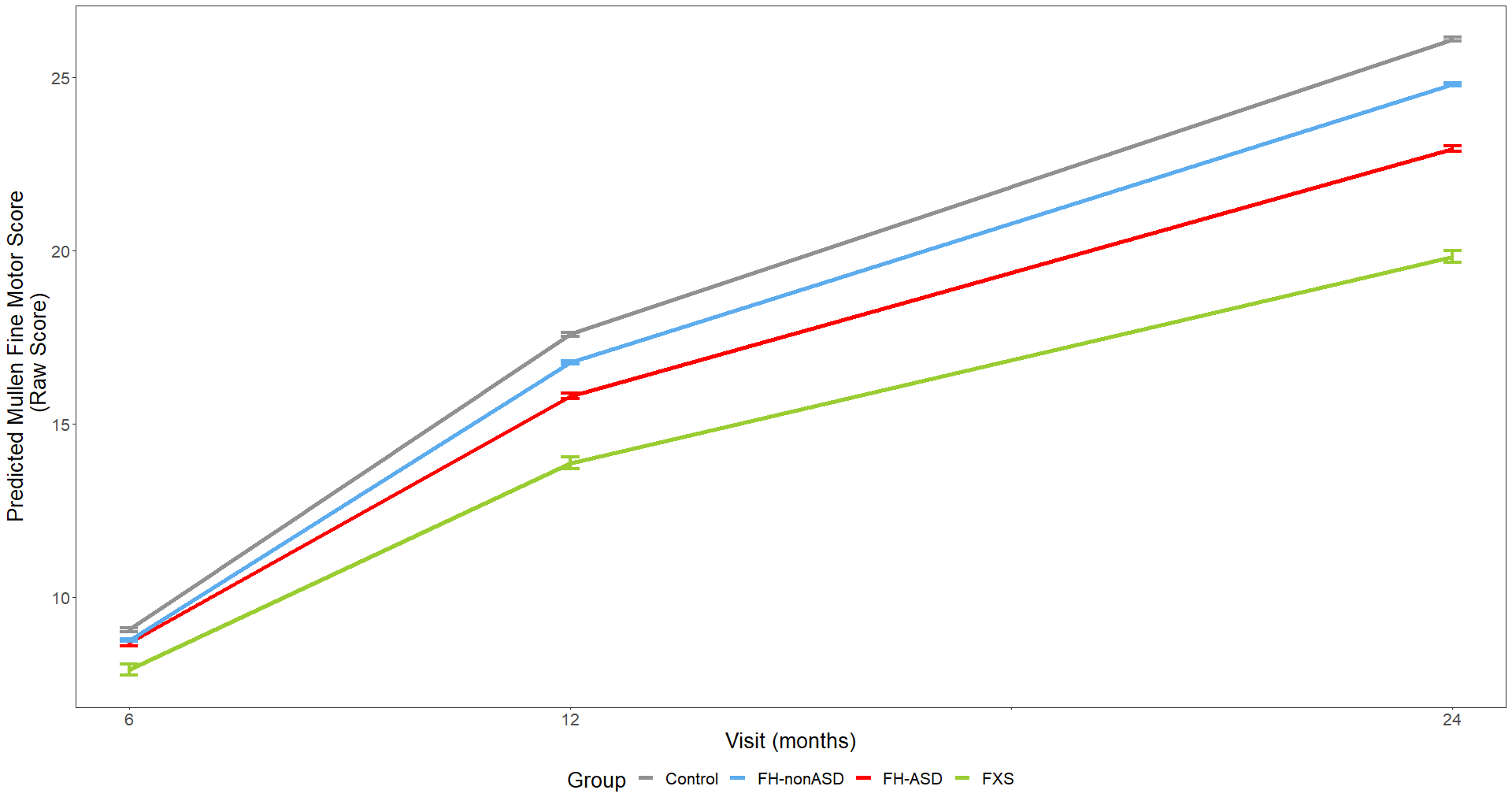


Model-generated differential trajectories of Fine Motor mean raw score from the MSEL by group and timepoint (Visit) for males only. At 6 months, no group trajectories significantly differed from one another. By 12 months of age a pattern emerged between groups (Table S5) that persisted through 24 months, such that control > FH-nonASD > FH-ASD > FXS.

Figure S16. MSEL Gross Motor Raw Score Trajectory by Group and Timepoint, Males Only


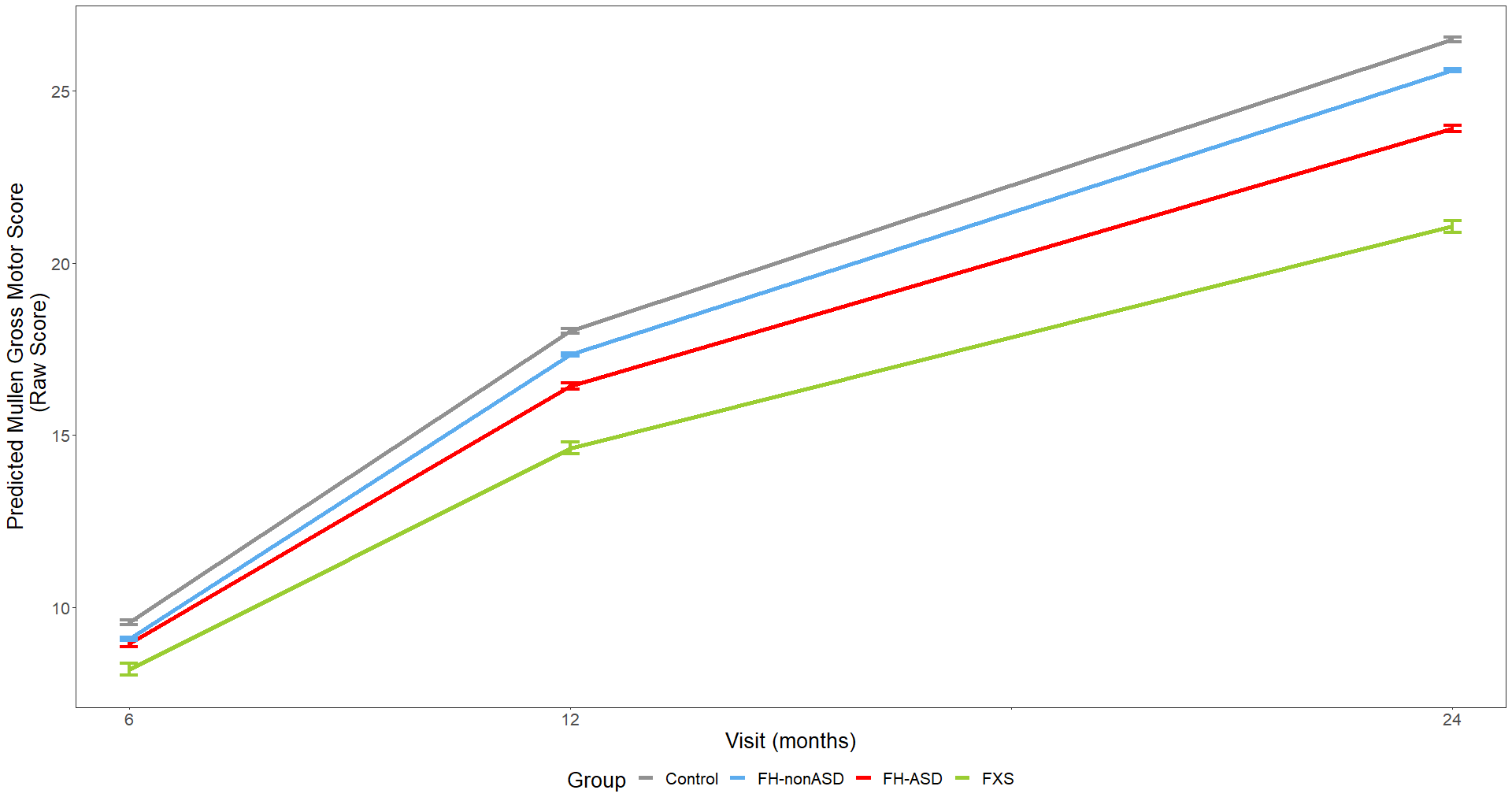


Model-generated differential trajectories of Gross Motor mean raw score from the MSEL by group and timepoint (Visit) for males only. At 6 months, no group trajectories significantly differed from one another. By 12 months of age a pattern emerged between groups (Table S5) that persisted through 24 months, such that all groups significantly differed from one another except for FH-nonASD and control groups.

Figure S17. MSEL Visual Reception Raw Score Trajectory by Group and Timepoint, Males Only


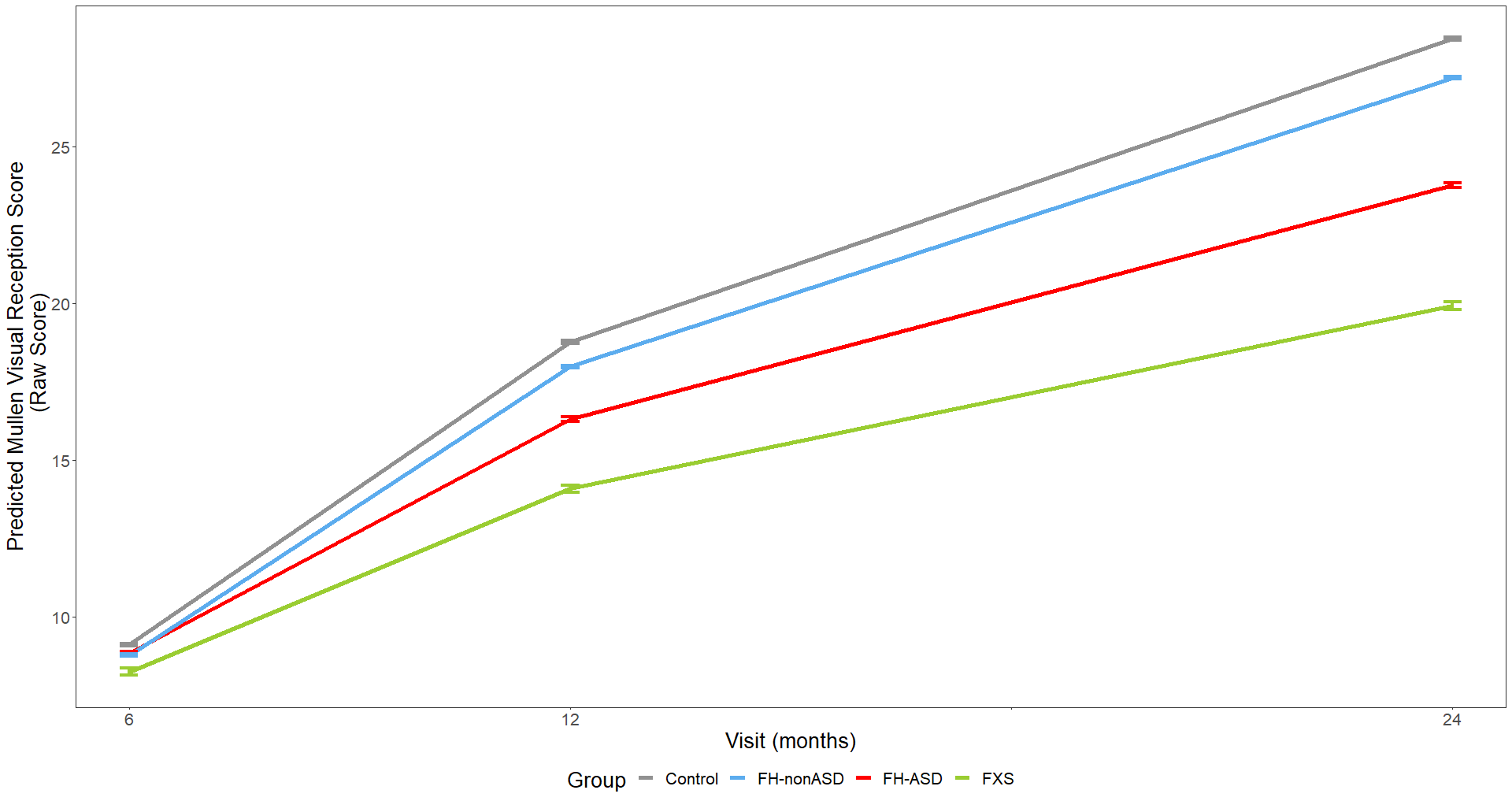


Model-generated differential trajectories of Visual Reception mean raw score from the MSEL by group and timepoint (Visit) for males only. At 6 months, no group trajectories significantly differed from one another. By 12 months of age a pattern emerged between groups (Table S5) that persisted through 24 months, such that all groups significantly differed from one another except for FH-nonASD and control groups.

Figure S18. NVDQ Trajectories by Cognitive Level Group and Timepoint


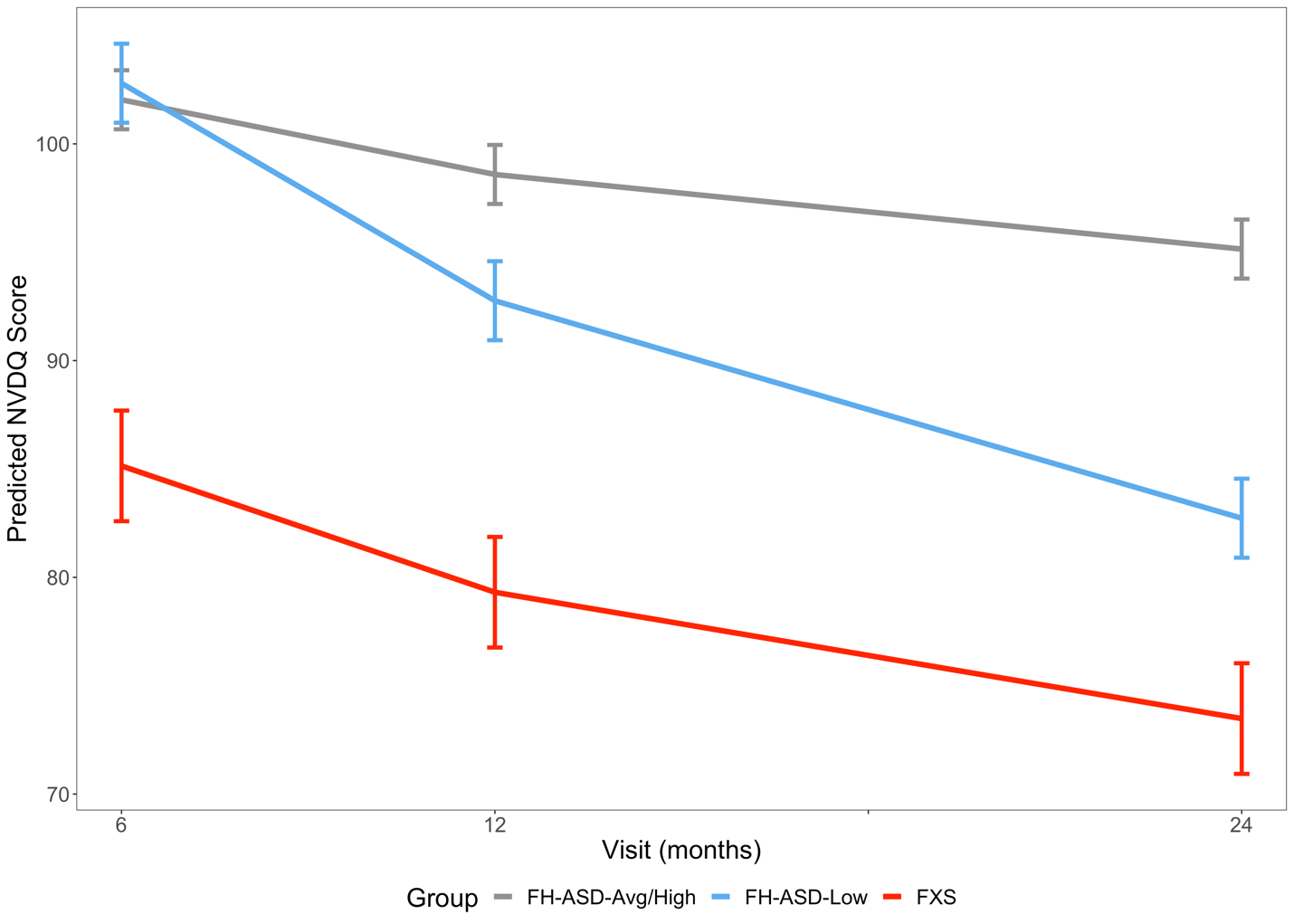


Model-generated differential trajectories of NVDQ score from the MSEL by cognitive level within the FH-ASD group and timepoint (Visit). FH-ASD infants with low cognitive level outperform FXS infants at 6 and 12 months, but do not statically differ from FXS at 24 months.

Figure S19. VDQ Trajectories by Cognitive Level Group and Timepoint


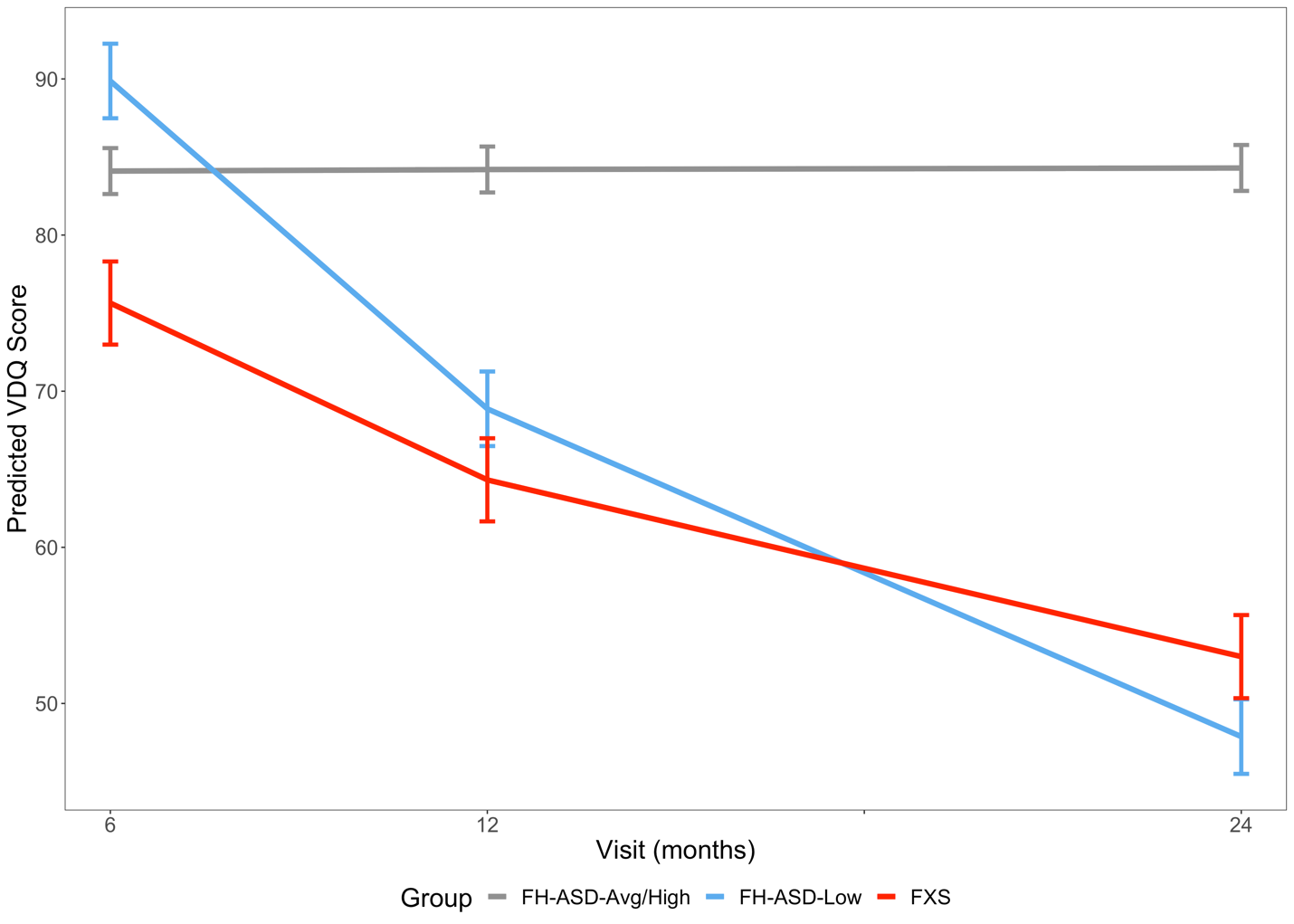


Model-generated differential trajectories of VDQ score from the MSEL by cognitive level within the FH-ASD group and timepoint (Visit). FH-ASD infants with low cognitive level outperform FXS infants at 6 months, but do not statically differ from FXS at 12 and 24 months.

Figure S20. Receptive Language Age Equivalent Trajectories by Cognitive Level Group and Timepoint


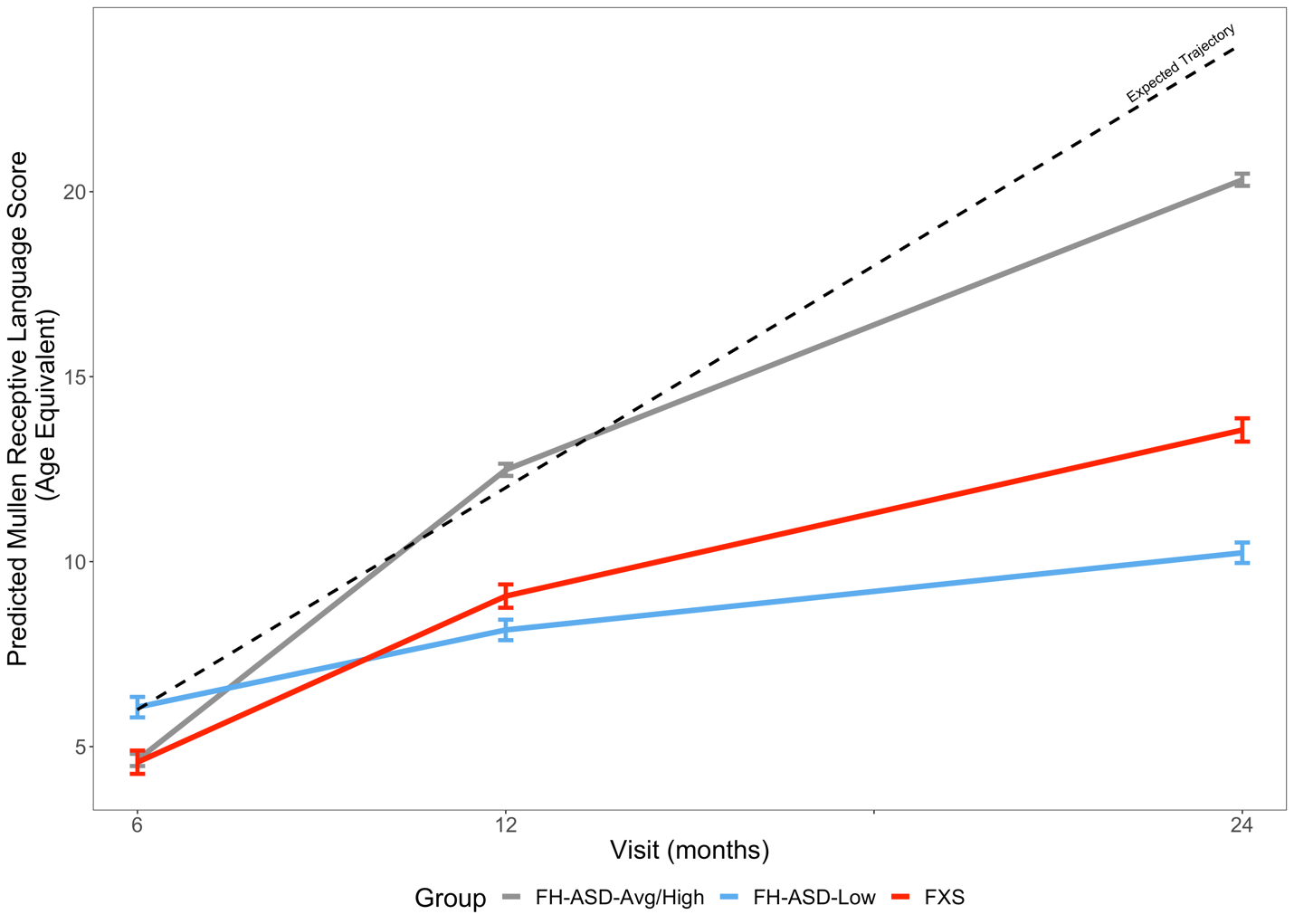


Model-generated differential trajectories of Receptive Language score from the MSEL by cognitive level within the FH-ASD group and timepoint (Visit). FH-ASD infants with low cognitive level perform similarly to FXS throughout infancy.

Figure S21. Expressive Language Age Equivalent Trajectories by Cognitive Level Group and Timepoint


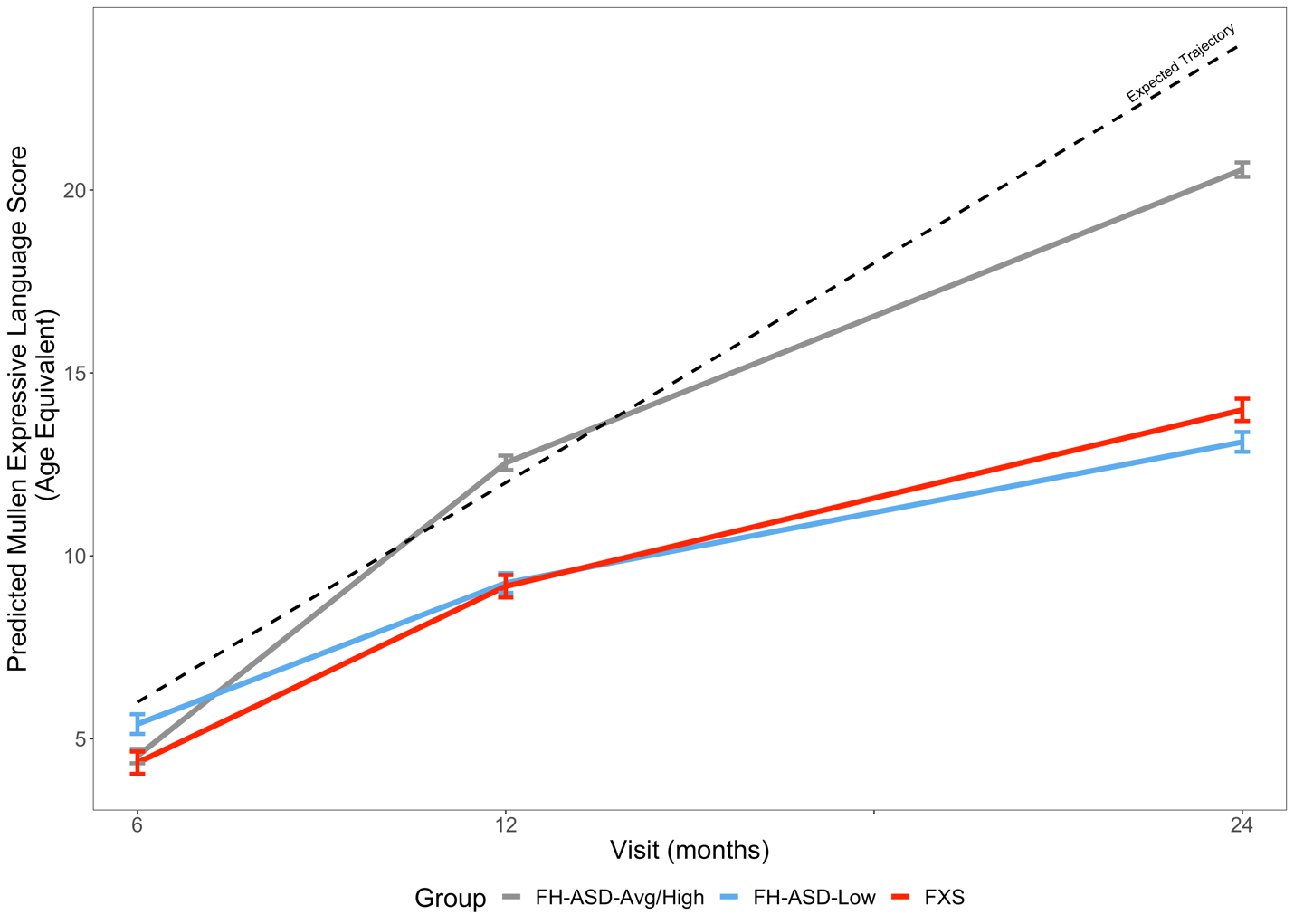


Model-generated differential trajectories of Expressive Language score from the MSEL by cognitive level within the FH-ASD group and timepoint (Visit). FH-ASD infants with low cognitive level perform similarly to FXS throughout infancy.

Figure S22. Fine Motor Age Equivalent Trajectories by Cognitive Level Group and Timepoint


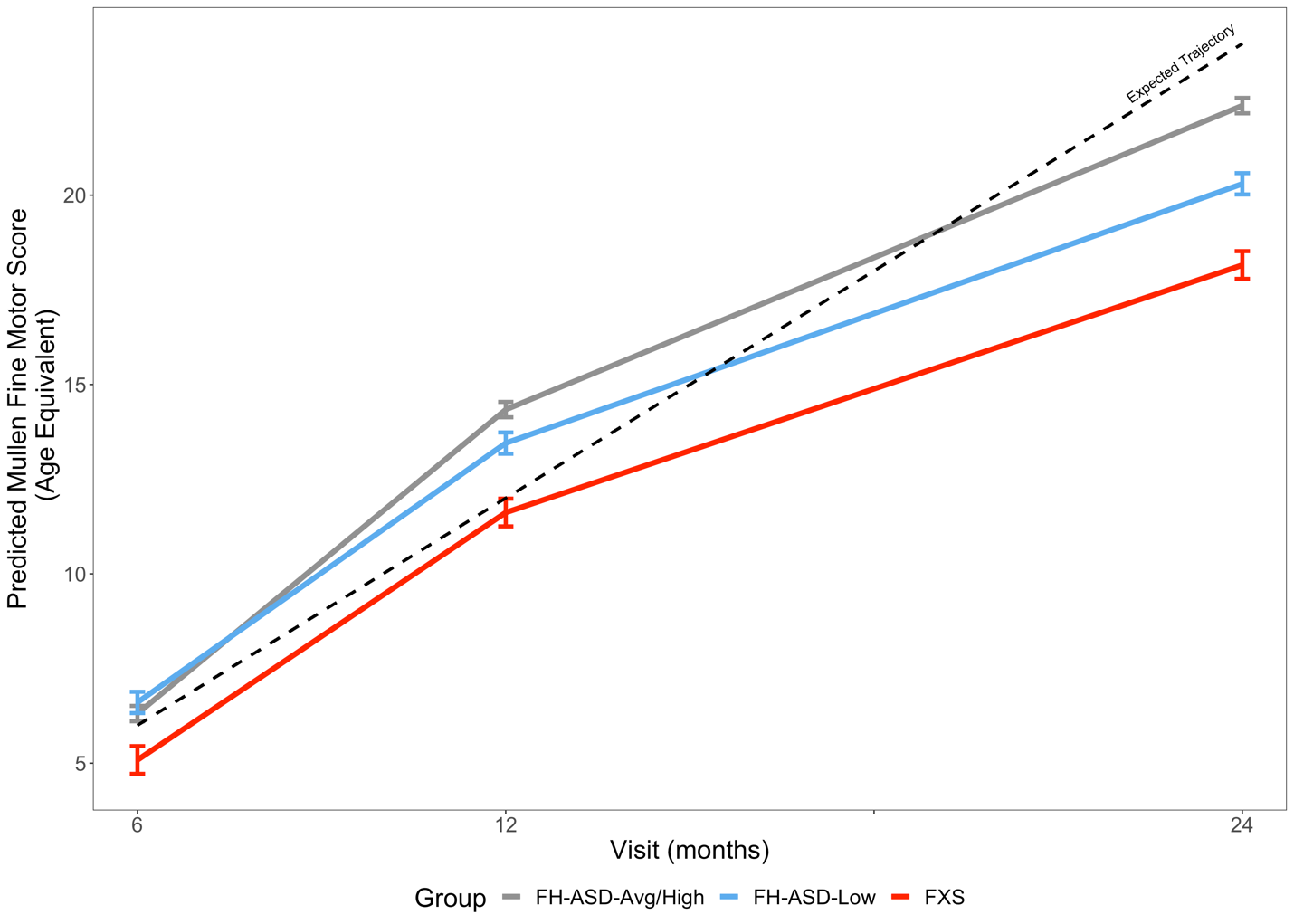


Model-generated differential trajectories of Fine Motor score from the MSEL by cognitive level within the FH-ASD group and timepoint (Visit). FH-ASD infants with low cognitive level do not differ from FXS at 6 months, but outperform FXS at 12 and 24 months.

Figure S23. Gross Motor Age Equivalent Trajectories by Cognitive Level Group and Timepoint


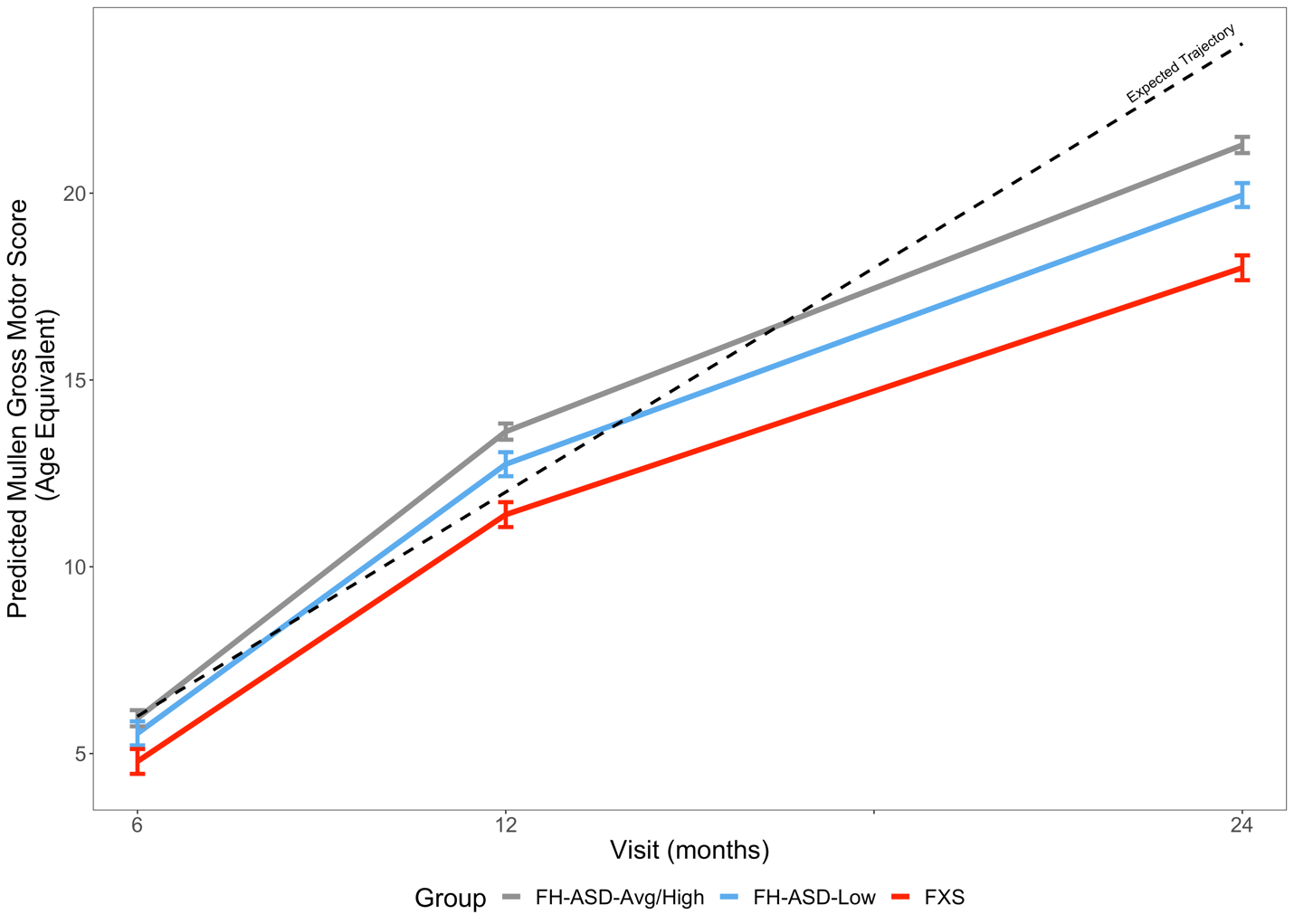


Model-generated differential trajectories of Gross Motor score from the MSEL by cognitive level within the FH-ASD group and timepoint (Visit). FH-ASD infants with low cognitive level do not differ from FXS at 6 months, but outperform FXS at 12 and 24 months.

Figure S24. Visual Reception Age Equivalent Trajectories by Cognitive Level Group and Timepoint


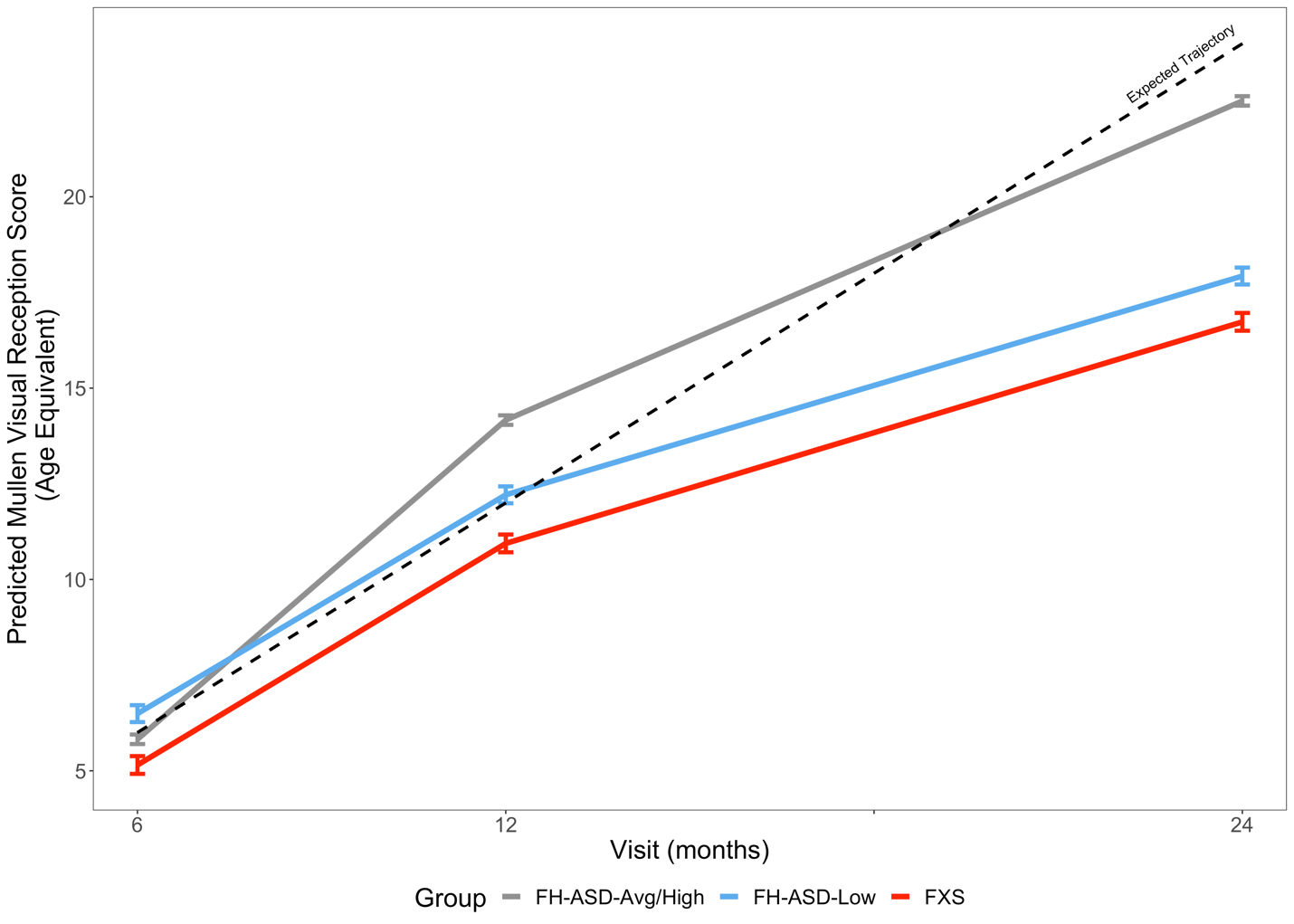


Model-generated differential trajectories of Visual Reception score from the MSEL by cognitive level within the FH-ASD group and timepoint (Visit). FH-ASD infants with low cognitive level perform similarly to FXS throughout infancy.
